# Inhibition of NPC1L1 disrupts adaptive responses of drug‐tolerant persister cells to chemotherapy

**DOI:** 10.15252/emmm.202114903

**Published:** 2022-01-13

**Authors:** Zhe Zhang, Siyuan Qin, Yan Chen, Li Zhou, Mei Yang, Yongquan Tang, Jing Zuo, Jian Zhang, Atsushi Mizokami, Edouard C Nice, Hai‐Ning Chen, Canhua Huang, Xiawei Wei

**Affiliations:** ^1^ Laboratory of Aging Research and Cancer Drug Target State Key Laboratory of Biotherapy and Cancer Center National Clinical Research Center for Geriatrics, West China Hospital Sichuan University Chengdu China; ^2^ State Key Laboratory of Biotherapy and Cancer Center West China Hospital, and West China School of Basic Medical Sciences & Forensic Medicine Sichuan University, and Collaborative Innovation Center for Biotherapy Chengdu China; ^3^ Department of Pediatric Surgery West China Hospital Sichuan University Chengdu China; ^4^ School of Medicine Southern University of Science and Technology Shenzhen Guangdong China; ^5^ Guangdong Provincial Key Laboratory of Cell Microenvironment and Disease Research Shenzhen China; ^6^ Department of Urology Graduate School of Medical Sciences Kanazawa University Kanazawa Japan; ^7^ Department of Biochemistry and Molecular Biology Monash University Clayton Vic Australia; ^8^ Department of Gastrointestinal Surgery State Key Laboratory of Biotherapy and Cancer Center West China Hospital Sichuan University, and Collaborative Innovation Center for Biotherapy Chengdu China

**Keywords:** cancer therapy, drug‐tolerant persister state, multidrug resistance, NPC1L1, oxidative stress, Cancer

## Abstract

Entering a drug‐tolerant persister (DTP) state of cancer cells is a transient self‐adaptive mechanism by which a residual cell subpopulation accelerates tumor progression. Here, we identified the acquisition of a DTP phenotype in multidrug‐resistant (MDR) cancer cells as a tolerance response to routine combination treatment. Characterization of MDR cancer cells with a DTP state by RNA‐seq revealed that these cells partially prevented chemotherapy‐triggered oxidative stress by promoting NPC1L1‐regulated uptake of vitamin E. Treatment with the NPC1L1 inhibitor ezetimibe further enhanced the therapeutic effect of combinatorial therapy by inducing methuosis. Mechanistically, we demonstrated that NRF2 was involved in transcriptional regulation of NPC1L1 by binding to the −205 to −215 bp site on its promoter. Decreased DNA methylation was also related partially to this process. Furthermore, we confirmed that a triple‐combination of chemotherapeutic agents, verapamil, and ezetimibe, had a significant anti‐tumor effect and prevented tumor recurrence in mice. Together, our study provides a novel insight into the role of DTP state and emphasizes the importance of disrupting redox homeostasis during cancer therapy.

## Graphical abstract

Drug‐tolerant persister (DTP) state is a driver of therapy failure and cancer relapse. This study identified a key role for NPC1L1 in multidrug‐resistant (MDR) cancer cells with the DTP state, where NPC1L1 orchestrated a redox signaling against the harsh environment caused by cancer therapy.

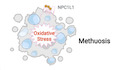

**The paper explained**

**Problem**

The failure of chemotherapy treatment is mostly due to the occurrence of multidrug resistance (MDR). A subset of residual cells exhibits a transient adaptive resistance mechanism by entering a drug‐tolerant persister (DTP) state after treatment. This emerging concept explains the generation of resistant clones with clinically relevant MDR. Accordingly, exploring the specific role and behavior of MDR cancer cells with DTP state in response to current therapy is important for delaying recurrence or even eradicating cancer by targeting specific vulnerabilities.

**Results**

We identified the acquisition of DTP phenotype in MDR1‐mediated MDR cancer cells as a tolerance response to the routine combination of chemotherapeutic agents and MDR1 inhibitor verapamil. MDR cancer cells with DTP state survived chemotherapy‐induced oxidative stress primarily by scavenging lipid ROS through NRF2‐NPC1L1 axis‐regulated vitamin E uptake. Based on nanoparticle‐related drug delivery systems to alleviate verapamil side effects *in vivo*, the combination of chemotherapeutic agents, verapamil, and NPC1L1 inhibitor ezetimibe demonstrated a significant anti‐tumor effect and prevented tumor recurrence in mice.

**Impact**

Our study provides key bearings on the relationship between DTP state and NPC1L1‐modulated oxidative stress defense by using MDR1‐mediated MDR cancer cells, which establishes a novel therapeutic strategy for treating MDR cancer cells and preventing tumor recurrence.

## Introduction

Drug resistance, either intrinsic or acquired, is omnipresent in the clinical treatment of cancer, limiting durable therapeutic benefits and even accelerating tumor recurrence or metastasis (Szakacs *et al,* 2006; Andrei *et al,* 2020; Dallavalle *et al,* 2020). Multidrug resistance, one of the most difficult problems occurring during chemotherapy, is commonly related to the expression of ATP‐binding cassette (ABC) transporters, especially transporter—multidrug resistance protein 1 (MDR1) encoded by *ABCB1* (Hall *et al,* 2009). Along with the emergence of appropriate analytical tools, advanced medicinal techniques, and multidisciplinary research, ample evidence not only identifies high expression of MDR1 portending a poor response to chemotherapy and adverse outcomes in patients with cancers but also confirms the necessity of applying new drug or drug delivery protocols to prevent cancer MDR in the clinic (Fan *et al,* 2017).

Unfortunately, the growing realization that cancer cells, rather than being either sensitive or resistant, can be dynamic and transient in nature within the context of treatment is detracting from such studies (Qin *et al,* 2020). There is actually, a particular state lying between sensitivity and resistance, termed the “drug‐tolerant persister (DTP) state,” in which a cell population is endowed with a dormant, slow‐cycling state and a stem‐like signature (Shen *et al*, 2019, 2020). The concept of DTP originates from an early observation of bacterial response to antibiotics, where the existence of residual bacteria exposed to antibiotics is due to non‐genetic variations and resumption of their initial characteristics upon interruption of treatment (Balaban *et al,* 2019). In the context of cancer, tumor cells share a similar situation in which drug resistance is, in a similar fashion to antibiotic resistance, driven by epigenetic inheritance of variant gene expression patterns. This could result in not only inhibiting the therapeutic efficacy but also providing a reservoir for further evolution (Shen *et al*, 2019, 2020). Given that this DTP state has been hypothesized to be part of an alternative approach toward a *bona fide* drug resistance mechanism, this line of research is attracting considerable attention. In addition to the recognition that DTP could serve as a target for therapy (*i.e.,* incorporation of an epigenetic modulator), studies focusing on DTP have deepened our understanding of the mechanisms driving DTP. This encourages the development of methods aimed at disposing of these cells, including sustaining a harmless dormant state, and reactivating proliferation to enhance response to anti‐proliferative drugs to eradicate them (Recasens & Munoz, 2019). However, there remains some confusion on how best to target DTP cells, indicating the requirement for a further understanding of both DTP itself and DTP‐mediated drug tolerance in order to devise countermeasures against these persisters.

To date, multiple studies have emphasized the impact of stress response on persister generation, and several teams have highlighted the capacity to adapt to oxidative stress as a common characteristic of cancer cells in a DTP state (Raha *et al,* 2014; Sahu *et al,* 2016; Hangauer *et al,* 2017; Anand *et al,* 2019; Dhimolea *et al,* 2021). Based on this characteristic, increasing numbers of drug targets involved in oxidative stress defense of DTP have been investigated. Among them the phospholipid glutathione peroxidase 4 (GPX4) is crucial for the survival of cancer cells in a therapy‐resistant state. In this context, subsequent work has shown that targeting GPX4‐dependent oxidative stress defense can almost completely eradicate persister cells by induction of ferroptosis, an oxidative cell death, suggesting a potential treatment strategy by disturbing the redox homeostasis (Hangauer *et al,* 2017). Additionally, activated NF‐E2‐related factor 2 (NRF2), detected in HER2‐inhibited persistent breast cancer, has been found to drive the re‐establishment of redox homeostasis in a glutathione metabolism‐dependent manner, thereby promoting the reactivation of dormant tumor cells. This reactivation triggered by NRF2 signaling can be prevented by glutaminase inhibition. This has also been shown to impair the growth of recurrent tumors with high levels of NRF2, suggesting a novel approach to treat NRF2 high dormant and recurrent cancer (Fox *et al,* 2020). Such findings confirm persister population adaption to therapy‐induced oxidative stress. Furthermore, MDR cancer cells might raise a robust antioxidant system for resisting oxidative stress caused by chemotherapeutic agents (Trachootham *et al,* 2009). We therefore questioned whether persister cancer cells arising from MDR cancer cells prefer to orchestrate the evolutionarily conserved antioxidant system against treatment.

In this study, we investigated how MDR cancer cells characterized by overexpression of MDR1 underwent a combinatorial therapy (chemotherapeutic agent with MDR1 inhibitor verapamil)‐induced DTP state. To gain insight into the alterations of gene expression profile in the course of non‐mutationally acquired resistance, we performed RNA‐seq comparing MDR persister cells to MDR cancer cells. We also investigated the function of screened genes in MDR cancer cells with a DTP status. This revealed that NPC1L1, an important regulator for redox homeostasis promoting uptake of vitamin E which can interact directly with lipid peroxyl radicals, thus preventing oxidative stress, was highly expressed in DTP. By adding the NPC1L1 inhibitor ezetimibe into the combinatorial therapy, the function of NPC1L1 on vitamin E absorption was compromised and additional cell death was observed as a consequence of macropinocytosis induction. Mechanistically, our results demonstrated a link between both NRF2 transcriptional activation and decreased DNA methylation with NPC1L1 expression. Using a nanomedicine approach to alleviate side effects of verapamil *in vivo*, we further confirmed the anti‐tumor effect of a triple‐combinatorial therapy strategy (a combination of chemotherapeutic agents, verapamil, and ezetimibe) that prevented tumor recurrence *in vivo*. Together, our study progresses our understanding of MDR persistence from a redox perspective, repositioning the potential of MDR therapy for treating cancer cells.

## Results

### Oncotherapy induces the transformation of MDR cancer cells into a DTP state

To elucidate the molecular mechanisms underlying the established drug‐resistant cancer cell models (Du145^TXR^ resistant to taxol; MCF‐7^ADR^ resistant to adriamycin) (Appendix Fig S1A and B), we first examined the expression of MDR1 (also known as p‐glycoprotein), which has been extensively studied in the context of drug‐resistant phenotype (Gouaze *et al,* 2005; Takeda *et al,* 2007; Robey *et al,* 2018) and confirmed the marked upregulation of its protein expression (Appendix Fig S1C). We further determined the role of MDR1 played in regulating the MDR phenotype using rhodamine123 (a substrate of MDR1) and MDR1 inhibitor verapamil. Fluorescence analysis indicated that MDR1 high expressing cancer cells showed increased efflux of rhodamine123 compared to control cancer cells, and verapamil could partially reverse this phenotype (Appendix Fig S1D). To investigate which combination of chemotherapeutic agent and verapamil contributed most to the anti‐cancer ability and synergistic effects, we examined cell viability using the Chou–Talalay method (combination index (CI) < 1 representing synergism) in different drug combination‐treated MDR cancer cells. Our results showed that 50 μM verapamil combined with 20 nM taxol or 200 nM adriamycin exhibited beneficial anti‐cancer effects in Du145^TXR^ cells or MCF‐7^ADR^ cells (Appendix Fig S1E and F). Furthermore, the proliferation of MDR cancer cells was significantly inhibited under the combination treatment, as evidenced by reduced colony formation (Appendix Fig S2A and B). Consistently, knockdown of *ABCB1* clearly decreased both cell viability and clonogenic growth capacity in chemotherapy‐treated MDR cancer cells (Appendix Fig S2C–G). Together with previous reports, our data support MDR1 as the key factor driving the MDR phenotype of these cancer cells and establish that targeting MDR1, at least in part, enables MDR cancer cells to recover chemosensitivity.

**Figure 1:**
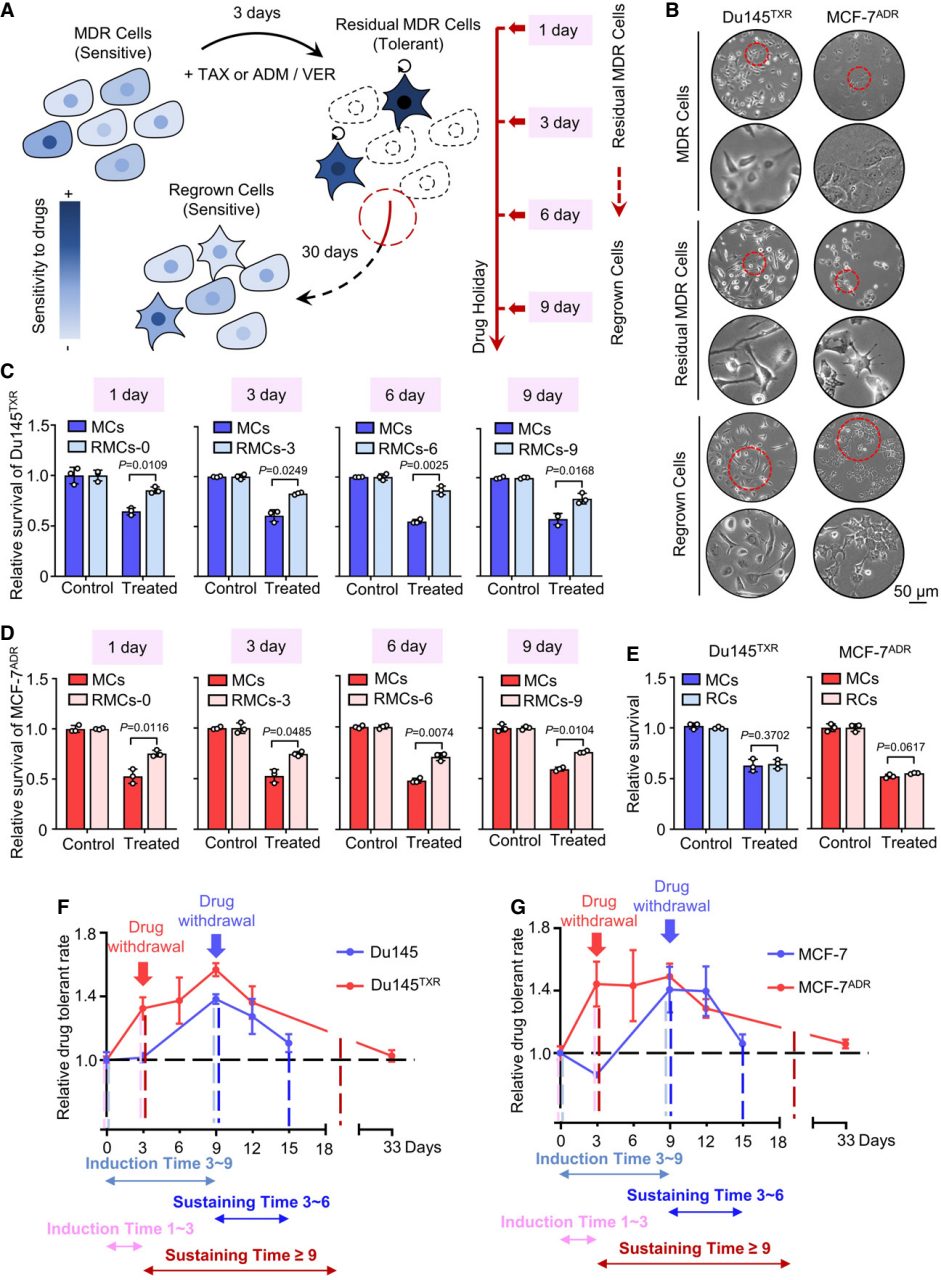
Oncotherapy induces the transformation of MDR cancer cells into a DTP state ASchematic of residual MDR cell generation and subsequent analyses. The circular arrow represents the DTP state.BPhase contrast images of the morphological changes in MDR cells, residual MDR cells, and regrown cells for Du145^TXR^ or MCF‐7^ADR^. Red circles mark magnified areas (bottom). Scale bars, 50 μm (low magnification images).C, DCell viability of residual MDR cells (RMCs) and MDR cells (MCs) of (C) Du145^TXR^ or (D) MCF‐7^ADR^ cells using the same combination treatment for 24 h on the indicated days. Student’s *t*‐test was used to analyze statistical differences. Mean with ± SD.ECell viability of regrown cells (RCs) and Du145^TXR^ (left) or MCF‐7^ADR^ (right) cells with the same combination treatment for 24 h. Student’s *t*‐test was used to analyze statistical differences. Mean with ± SD.F, GDrug‐tolerant rate of (F) Du145/Du145^TXR^ (20 nM taxol/20 nM taxol plus 50 μM verapamil) or (G) MCF‐7/MCF‐7^ADR^ (200 nM adriamycin/200 nM adriamycin plus 50 μM verapamil) cells treated with drugs on the indicated days. Drug‐tolerant rate defined by comparing cell viability followed treatment, RMCs vs. MCs or CCs vs. PeCs, > 1 represents drug tolerant. Induction time indicates the time of entering DTP state; and sustaining time indicates the time of maintaining DTP state. Blue colors represent control groups; Red colors represent MDR cancer cells. Mean with ± SD. Data information: Results are representative of three independent experiments. Source data are available online for this figure.

**Figure EV1:**
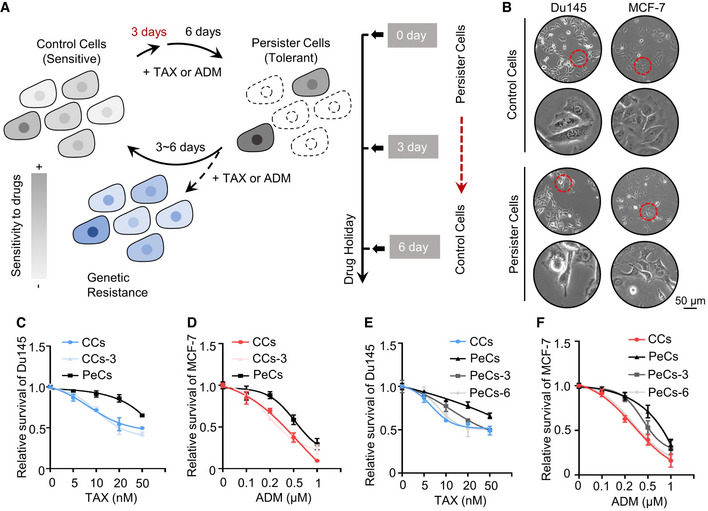
Control cancer cells transform into DTP states in response to chemotherapy ASchematic of drug persister cancer cell generation and subsequent analyses.BPhase contrast images of the morphological change in Du145 or MCF‐7 cells followed by chemotherapy treatment for 72 h. Red circles mark magnified areas (bottom). Scale bar, 50 μm (low‐magnification images).C, DCell viability of control cancer cells (CCs), 3 days treated control cancer cells (CCs‐3), and persister cancer cells (PeCs) of (C) Du145 or (D) MCF‐7 cells treated with the indicated concentrations of taxol (TAX) or adriamycin (ADM) for 24 h. Mean with ± SD.E, FCell viability of CCs, PeCs, 3 days drug withdrawn PeCs (PeCs‐3), and 6 days drug withdrawn PeCs (PeCs‐6) of (E) Du145 or (F) MCF‐7 cells treated with the indicated concentrations of taxol (TAX) or adriamycin (ADM) for 24 h. Mean with ± SD. Data information: Results are representative of three independent experiments. Source data are available online for this figure.

Notably, we observed the occurrence of residual MDR cancer cells following combination treatment. These cells were similar to those described for drug‐tolerant persister (DTP) cells, which survive cytotoxic exposure via reversible and non‐mutational mechanisms. To evaluate whether these residual MDR cancer cells were associated with the DTP state, we examined the cell viability and observed morphological changes in Du145^TXR^ cells and MCF‐7^ADR^ cells following combination treatment (50 μM verapamil/20 nM taxol or 200 nM adriamycin). Surprisingly, compared with MDR cancer cells (MCs), which had not been treated with the combinatorial therapy, residual MDR cancer cells (RMCs) showed dramatic morphological alterations following combination treatment for 3 days and obvious tolerance to a second exposure of treatment applied 1, 3, 6, and 9 days after treatment withdrawal as shown by a cell viability assay (Fig 1A–D). Subsequently, a long‐term “drug holiday” (30 days) allowed residual MDR cancer cells to regrow (regrown cells, RCs) and resulted in re‐acquisition of sensitivity to combination treatment (Fig 1E). The reversibility of further drug resistance in residual MDR cancer cells implies that oncotherapy drives MDR cancer cells into a DTP state, termed as MDR persister cells (MPCs). To compare the differences between MPCs and persister cancer cells (PeCs), we used a similar protocol to generate DTP cells in control cells (CCs) (Fig EV1A). Our results indicated that prolonged drug exposure in control cells for > 3 days could generate a small population of DTP cells (Fig EV1B–D). In addition, DTP cells progressively re‐acquired drug sensitivity following withdrawal of chemotherapy and eventually became non‐differential compared with control cells by Day 6 as evidenced by cell viability (Fig EV1E and F). Accordingly, we converted the above data obtained by cell viability assays (Figs 1C–E and EV1C–F) into a drug‐tolerant rate. As shown in Fig 1F and G, MDR cancer cells can further evolve upon treatment compared with control cells as evidenced by faster induction and a longer duration of the DTP state. Taken together, our results demonstrate that MDR cancer cells can transform into a DTP state resembling control cells, possibly leading to a worse outcome such as modest MDR reversal and tumor recurrence.

### Upregulation of NPC1L1 supports cell survival in response to cytotoxic stress in MPCs

Given the well‐known characteristics of DTP cells, such as induction of cell cycle arrest, increased expression of stemness markers, as well as activation of epithelial–mesenchymal transition (EMT) (Shen *et al*, 2019, 2020), we investigated whether MPCs had similar phenotypes. Our data indicated that MPCs seemed to be in a state of cell cycle arrest, as evidenced by significant increases in p27 and p21 expression (Appendix Fig S3A). In addition, we further performed flow cytometry analysis which demonstrated that cell cycle arrest at G0/G1 phase was induced in MPCs, showing a non‐proliferative or slowly proliferative state (Appendix Fig S3B–D). We also observed the upregulation of stemness markers ALDH1A1, Oct‐4A, Sox2, KLF4, and CD44 in MPCs (Appendix Fig S3A). Consistently, sphere formation assays showed that MPCs enhanced self‐renewal ability due to the increased stemness, as evidenced by a marked increase in both the number and size of MPCs spheres (Appendix Fig S3E–G). Interestingly, cell viability and colony formation assays revealed that ALDH1A1 inhibitor CM10 was most selectively lethal to MPCs cells and sensitized MCs to the combination treatment consistent with previous persister cell studies (Raha *et al,* 2014; Appendix Fig S3H–J). In addition to these characteristics, downregulation of epithelial marker E‐cadherin and upregulation of mesenchymal marker Vimentin were detected by Western blotting, implying induction of EMT (Appendix Fig S3A). Taken together, these data support our model for a similar phenotype to persister cells.

**Figure 2:**
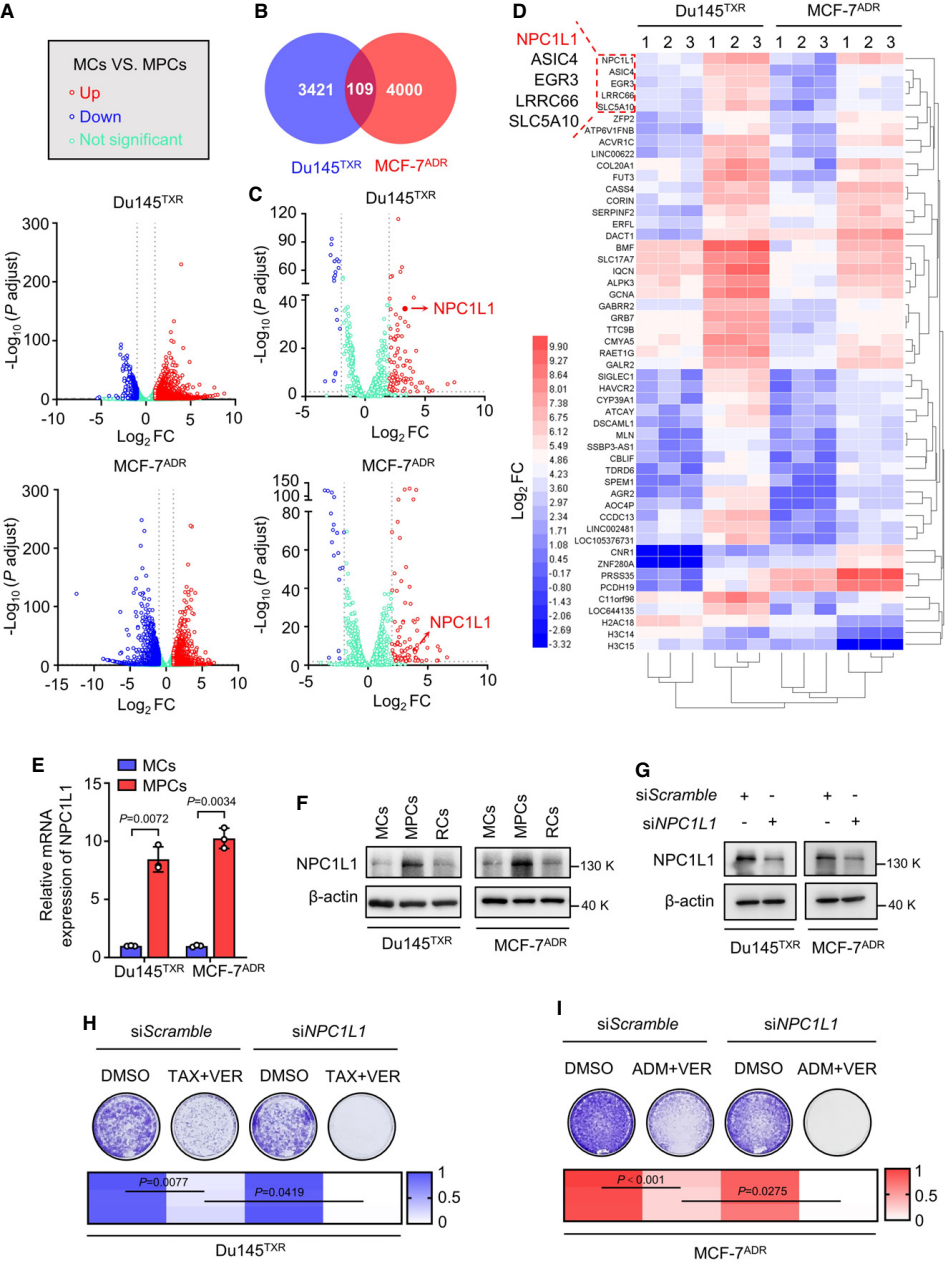
Upregulation of NPC1L1 supports cell survival in response to cytotoxic stress in MPCs AVolcano plot showing expressed genes identified by RNA‐seq of MCs vs. MPCs in Du145^TXR^ or MCF‐7^ADR^. Dotted lines represent the screening criteria (|fold change| ≥ 2, *P* ≤ 0.05).BVenn diagram indicating the overlaps of expressed genes in (A).CVolcano plot revealed 109 consistently expressed genes identified by RNA‐seq of MCs vs. MPCs in Du145^TXR^ and MCF‐7^ADR^. Dotted lines represent the screening criteria (|fold change| ≥ 2, *P* ≤ 0.05).DHeat map based on scoring rank indicated consistently expressed genes identified by RNA‐seq of MCs vs. MPCs in Du145^TXR^ and MCF‐7^ADR^.EqRT‐PCR analysis of NPC1L1 in MCs and MPCs of Du145^TXR^ or MCF‐7^ADR^. Student’s *t*‐test was used to analyze statistical differences. Mean with ± SD.FImmunoblotting of NPC1L1 in MCs, MPCs, and RCs of Du145^TXR^ or MCF‐7^ADR^.GImmunoblotting of NPC1L1 in Du145^TXR^ or MCF‐7^ADR^ cells transfected with si*NPC1L1* or si*Scramble*.H, IColony formation assay and quantification of (H) Du145^TXR^ or (I) MCF‐7^ADR^ cells transfected with si*NPC1L1* or si*Scramble* followed by treatment with indicated agents. One‐way ANOVA was used to analyze statistical differences. Data information: Results are representative of three independent experiments. Source data are available online for this figure.

**Figure EV2:**
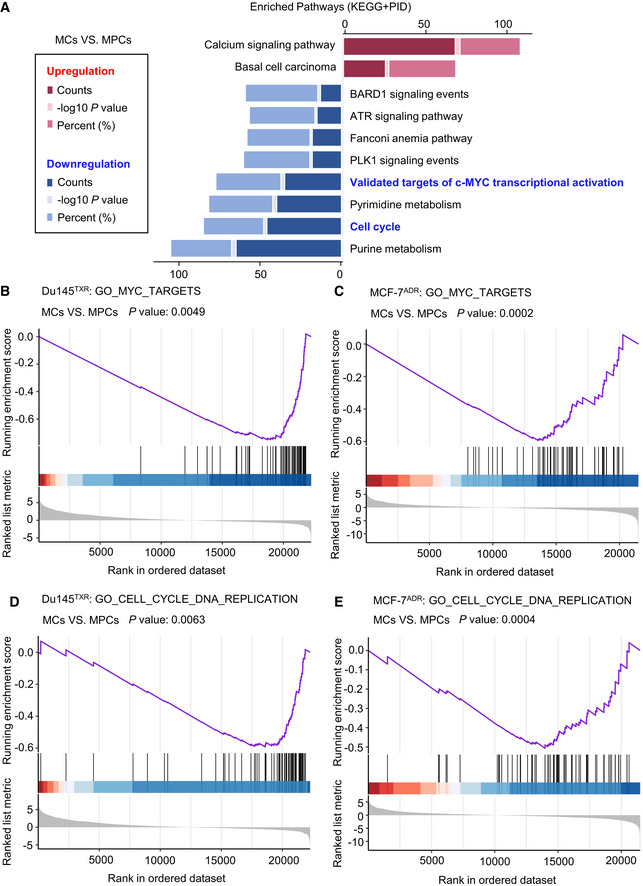
Signaling pathways identified in MPCs by performing RNA‐seq AKEGG/PID pathway analysis showed the changed signaling pathways of MCs vs. MPCs in Du145^TXR^ and MCF‐7^ADR^.B, CGSEA indicated GO Myc targets of MCs vs. MPCs in (B) Du145^TXR^ and (C) MCF‐7^ADR^.D, EGSEA indicated GO cell cycle and DNA replication of MCs vs. MPCs in (D) Du145^TXR^ and (E) MCF‐7^ADR^.

The attempt to target persister cells, especially MDR persister cells, as a therapeutic approach for overcoming the clinical bottleneck has long been enigmatic. To identify cellular processes or key effectors that may play a role in MPCs survival, RNA sequencing (RNA‐Seq) was conducted by comparing the gene expression profiles between MCs and MPCs, through which 3421 and 4000 differentially expressed genes (DEGs) were filtered for statistical significance (|fold change| ≥ 2, *P* ≤ 0.05) in Du145^TXR^ and MCF‐7^ADR^ cell lines, respectively (Fig 2A). These upregulated or downregulated gene sets were then grouped by pathway enrichment analysis using the ConsensusPathDB Bioinformatic tool (http://cpdb.molgen.mpg.de/). The results suggested that various genes, for example, those involved in cell cycle arrest and Myc pathway inactivation, which had been widely reported in previous studies (Dhimolea *et al,* 2021; Rehman *et al,* 2021), were significantly enriched in Du145^TXR^ or MCF‐7^ADR^ cells (Figs 2A and EV2A–E). In order to further identify the shared patterns of expression across two cell lines, 109 consistently upregulated or downregulated genes in MPCs compared with their corresponding MCs (between Du145^TXR^ vs. persister Du145^TXR^ and MCF‐7^ADR^ vs. persister MCF‐7^ADR^) were identified (Fig 2B–D). Given that cancer cells in the DTP state are dormant with a necessarily global reduction in protein neosynthesis, we mainly focused on translationally upregulated mRNAs that might be involved in persistence. As shown in Fig 2D, Niemann‐Pick C1 Like 1 (NPC1L1) was the top scoring candidate of the upregulated genes. Notably, NPC1L1 is a direct target of anti‐lipemic agent ezetimibe, and moreover, a promising target for clinical treatment (Bays, 2002).

To validate the RNA‐seq results presented above, we confirmed the upregulated expression of *NPC1L1* in MPCs compared with their corresponding MCs by quantitative real‐time PCR analysis (Fig 2E). In addition, NPC1L1 was examined in MCs, MPCs, and RCs by Western blotting, which demonstrated that protein levels of NPC1L1 were significantly increased in MPCs and returned back to their basal levels in RCs, consistent with the reversible drug‐resistant capacity of MDR cancer cells during the treatment (Fig 2F). We hypothesized that NPC1L1 inhibition in combination with chemotherapy agents/verapamil might usefully improve outcomes of current drug‐resistant reverse therapy in MDR cancer cells by directly targeting MDR persister cells. We therefore evaluated the efficacy of this strategy using a colony formation assay and found that siRNA‐mediated silencing of *NPC1L1* resulted in a further decrease in colony formation in chemotherapy agents/verapamil‐treated MDR cancer cells (Fig 2G–I). Collectively, these results reveal that NPC1L1 is a crucial regulator of DTP state and a potentially rational target for tumor patients with MDR disease in the clinic.

### NPC1L1 compromises oxidative stress in MDR cancer cells by promoting vitamin E uptake during chemotherapeutic agents/verapamil treatment

**Figure 3:**
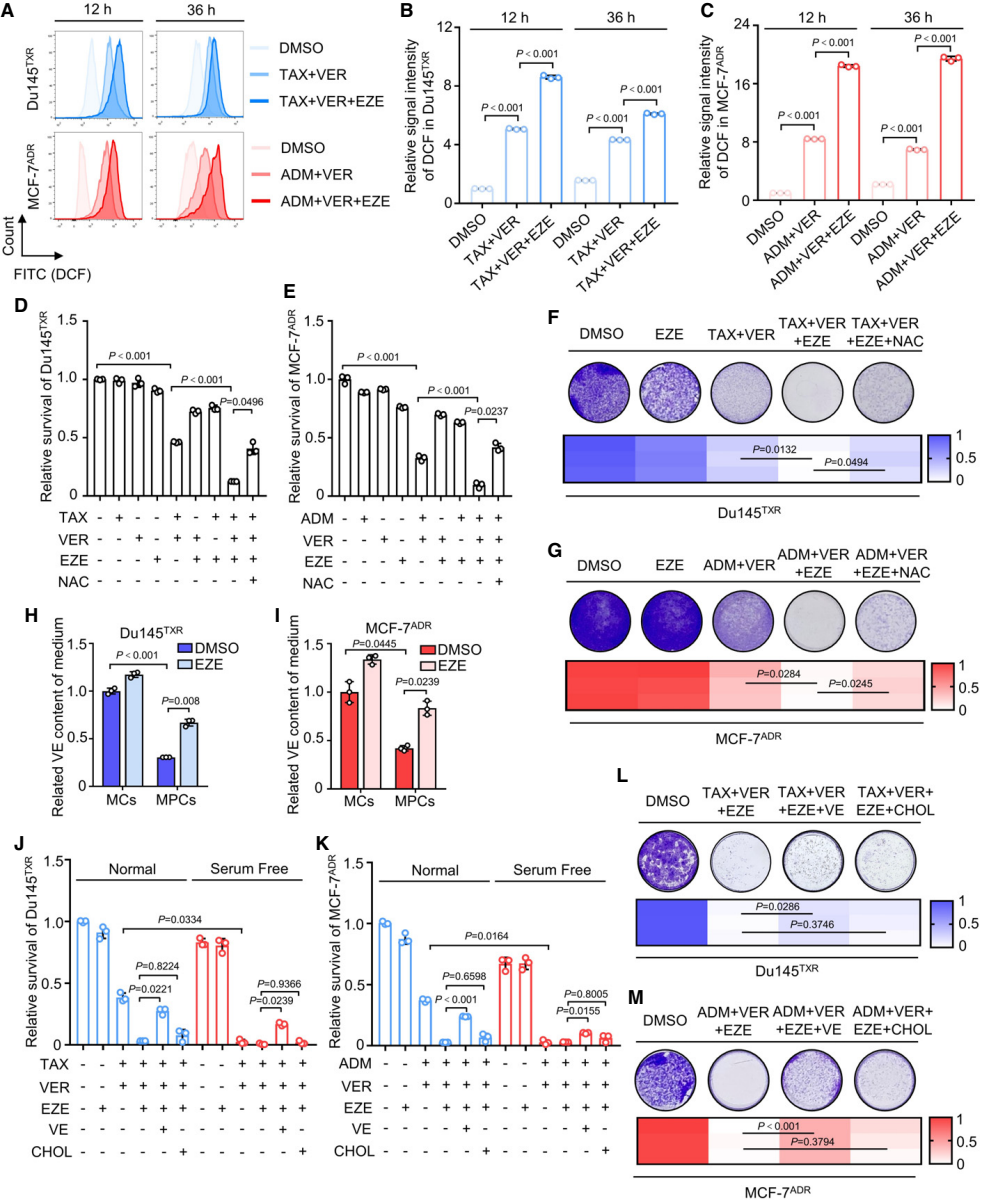
NPC1L1 compromises oxidative stress in MDR cancer cells by promoting vitamin E uptake during chemotherapeutic agents/verapamil treatment A–CFlow cytometric analysis of ROS levels in Du145^TXR^ or MCF‐7^ADR^ cells treated with indicated agents for 12 and 36 h. (A) Representative images and quantification of ROS in (B) Du145^TXR^ or (C) MCF‐7^ADR^ cells are shown. One‐way ANOVA was used to analyze statistical differences. Mean with ± SD.D, ECell viability of (D) Du145^TXR^ or (E) MCF‐7^ADR^ treated with indicated agents for 72 h. One‐way ANOVA was used to analyze statistical differences. Mean with ± SD.F, GColony formation assay and quantification of (F) Du145^TXR^ or (G) MCF‐7^ADR^ cells treated with indicated agents. One‐way ANOVA was used to analyze statistical differences.H, ICellular vitamin E analysis of medium supernatant in MCs and MPCs of (H) Du145^TXR^ or (I) MCF‐7^ADR^ cells treated with 50 μM vitamin E (VE) followed by treatment with or without 25 μM ezetimibe for 12 h. Student’s *t*‐test was used to analyze statistical differences. Mean with ±SD.J, KCell viability of (J) Du145^TXR^ or (K) MCF‐7^ADR^ cells cultured in normal or serum‐free medium followed by treatment with indicated agents for 72 h. One‐way ANOVA was used to analyze statistical differences. Mean with ± SD.L, MColony formation assay and quantification of (L) Du145^TXR^ or (M) MCF‐7^ADR^ cells treated with indicated agents. One‐way ANOVA was used to analyze statistical differences. Data information: Results are representative of three independent experiments.

**Figure EV3:**
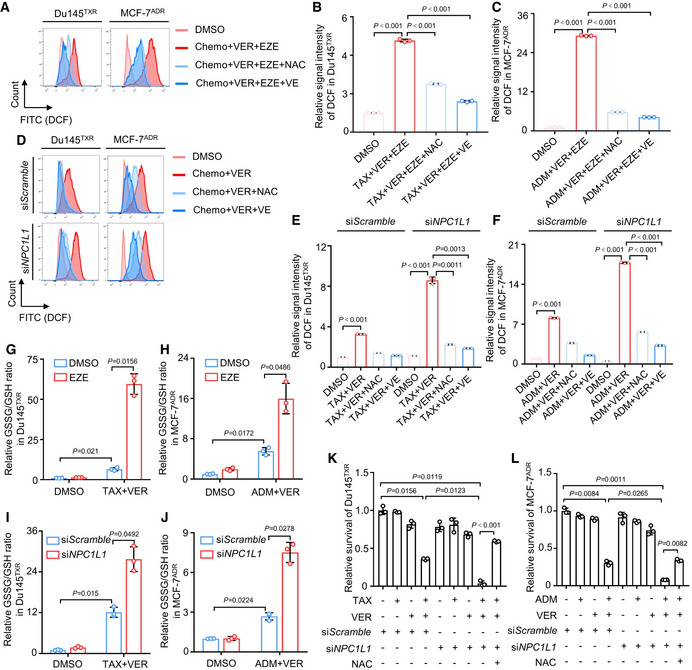
Inhibition of NPC1L1 induces oxidative stress‐mediated cell death A–CFlow cytometric analysis of ROS levels in Du145^TXR^ or MCF‐7^ADR^ cells treated with indicated agents for 24 h. Chemo represents 20 nM taxol or 200 nM adriamycin for Du145^TXR^ or MCF‐7^ADR^, respectively. (A) Representative images and quantification of ROS in (B) Du145^TXR^ or (C) MCF‐7^ADR^ cells are shown. One‐way ANOVA was used to analyze statistical differences. Mean with ± SD.D–FFlow cytometric analysis of ROS levels in Du145^TXR^ or MCF‐7^ADR^ cells transfected with si*NPC1L1* or si*Scramble* followed by indicated treatment for 24 h. Chemo represents 20 nM taxol or 200 nM adriamycin for Du145^TXR^ or MCF‐7^ADR^, respectively. (D) Representative images and quantification of ROS in (E) Du145^TXR^ or (F) MCF‐7^ADR^ cells are shown. One‐way ANOVA was used to analyze statistical differences. Mean with ± SD.G, HGSSG/GSH ratio measurement of (G) Du145^TXR^ or (H) MCF‐7^ADR^ cells treated with indicated agents for 24 h. One‐way ANOVA was used to analyze statistical differences. Mean with ± SD.I, JGSSG/GSH ratio measurement of (I) Du145^TXR^ or (J) MCF‐7^ADR^ transfected with si*NPC1L1* or si*Scramble* followed by indicated treatment for 24 hours. One‐way ANOVA was used to analyze statistical differences. Mean with ± SD.K, LCell viability of (K) Du145^TXR^ or (L) MCF‐7^ADR^ cells transfected with si*NPC1L1* or si*Scramble* followed by treatment with the indicated agents for 72 h. One‐way ANOVA was used to analyze statistical differences. Mean with ± SD. Data information: Results are representative of three independent experiments.

Based on the current knowledge of MDR cancer cells, these special populations are acknowledged to have specific dependency on the antioxidant system (Trachootham *et al,* 2009; Viswanathan *et al,* 2017; Wang *et al,* 2018a,b). In addition, a number of studies have recently reported targeting persistence based on redox modulation (Sahu *et al,* 2016; Hangauer *et al,* 2017; Takahashi *et al,* 2018; Fox *et al,* 2020). Accordingly, these findings directed our attention toward whether NPC1L1 was involved in oxidative stress defense mechanisms in MPCs. To test whether MDR cancer cells underwent oxidative stress during the combination treatment, reactive oxygen species (ROS) analysis using the fluorescence probe DCFH‐DA was performed. The results revealed that the intracellular ROS levels rapidly accumulated in 12 and 36 h following combination treatment, while pharmacological inhibition of NPC1L1 using ezetimibe (25 μM) further enhanced combination treatment‐induced ROS accumulation. This tended to be sustained at a high level for a relatively long time (Fig 3A–C). We next examined ROS levels in Du145^TXR^ and MCF‐7^ADR^ cells following triple‐combinatorial treatment with or without the antioxidants (5 mM N‐Acetyl‐L‐cysteine (NAC) and 50 μM vitamin E (VE)) by investigating DCF fluorescence intensity with flow cytometry. As shown in Fig EV3A–C, triple‐combinatorial treatment induced marked accumulation of ROS levels, while this accumulation could be compromised by NAC or vitamin E. Consistently, knockdown of NPC1L1 by si*NPC1L1* significantly enhanced combination treatment‐induced ROS accumulation. In contrast, either NAC or vitamin E visibly decreased ROS levels following combination treatment in si*NPC1L1*‐transfected cancer cells (Fig EV3D–F). Moreover, we also observed that ezetimibe induced an obvious upregulation of the ratio of oxidized glutathione (GSSG) to reduced glutathione (GSH) in combination treatment‐stimulated cancer cells (Fig EV3G and H). As expected, NPC1L1 knockdown induced a similar outcome to ezetimibe (Fig EV3I and J). Importantly, either ezetimibe or si*NPC1L1*‐mediated knockdown led to both remissive cell viability and colony formation in combination treatment. In contrast, NAC compromised triple‐combinatorial treatment‐induced growth inhibition (Figs 3D–G and EV3K and L). These results indicated that NPC1L1 might contribute to ROS neutralization which was necessary for MDR cancer cell survival.

**Figure EV4:**
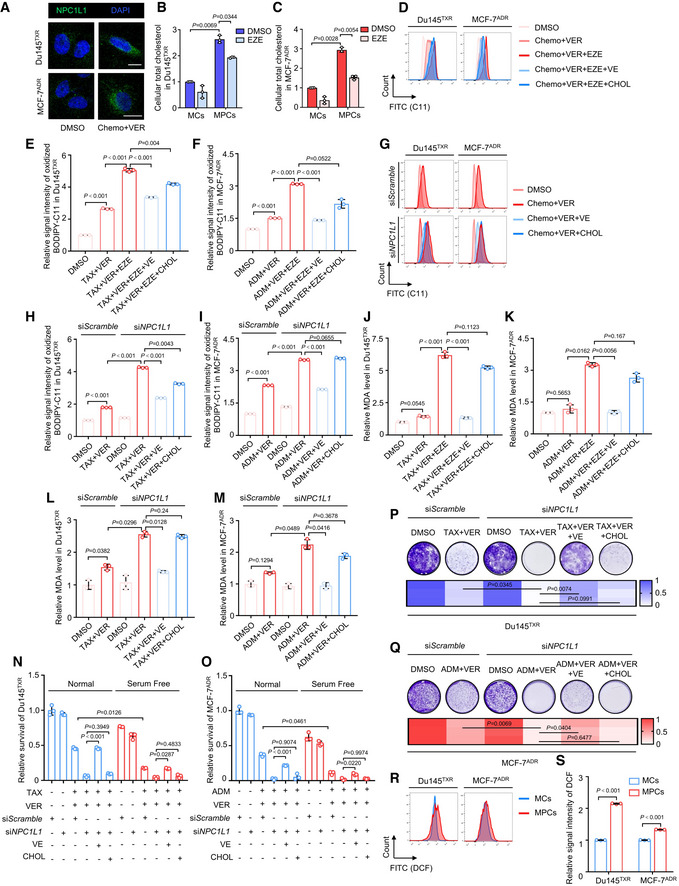
Lipotoxicity from NPC1L1 inhibition‐induced oxidative stress may partially contribute to cell death of MPCs AImmunofluorescence analysis of NPC1L1 in Du145^TXR^ and MCF‐7^ADR^ treated with indicated agents for 12 h. Scale bar, 10 μm.B, CCellular total cholesterol analysis in MCs and MPCs of (B) Du145^TXR^ or (C) MCF‐7^ADR^ cells treated with 25 μM ezetimibe for 12 h. One‐way ANOVA was used to analyze statistical differences. Mean with ± SD.D–FFlow cytometric analysis of lipid ROS using BODIPY 581/591 C11 reagent in Du145^TXR^ or MCF‐7^ADR^ cells treated with indicated agents for 24 h. Chemo represents 20 nM taxol or 200 nM adriamycin for Du145^TXR^ or MCF‐7^ADR^, respectively. (D) Representative images and quantification of lipid ROS in (E) Du145^TXR^ or (F) MCF‐7^ADR^ cells are shown. One‐way ANOVA was used to analyze statistical differences. Mean with ± SD.G–IFlow cytometric analysis of lipid ROS using BODIPY 581/591 C11 reagent in Du145^TXR^ or MCF‐7^ADR^ cells transfected with si*NPC1L1* or si*Scramble* followed by indicated treatment for 24 h. Chemo represents 20 nM taxol or 200 nM adriamycin for Du145^TXR^ or MCF‐7^ADR^, respectively. (G) Representative images and quantification of lipid ROS in (H) Du145^TXR^ or (I) MCF‐7^ADR^ cells are shown. One‐way ANOVA was used to analyze statistical differences. Mean with ± SD.J–KMDA levels measurement of (J) Du145^TXR^ or (K) MCF‐7^ADR^ cells treated with indicated agents for 24 h. One‐way ANOVA was used to analyze statistical differences. Mean with ± SD.L, MMDA levels in (L) Du145^TXR^ or (M) MCF‐7^ADR^ cells transfected with si*NPC1L1* or si*Scramble* followed by indicated treatment for 24 h. One‐way ANOVA was used to analyze statistical differences. Mean with ± SD.N, OCell viability of (N) Du145^TXR^ or (O) MCF‐7^ADR^ cells transfected with si*NPC1L1* or si*Scramble* followed by treatment with the indicated agents cultured in normal or serum‐free medium for 72 h. One‐way ANOVA was used to analyze statistical differences. Mean with ± SD.P, QColony formation assay and quantification of (P) Du145^TXR^ or (Q) MCF‐7^ADR^ cells transfected with si*NPC1L1* or si*Scramble* followed by treatment with indicated agents. One‐way ANOVA was used to analyze statistical differences.R, SFlow cytometric analysis of ROS levels in MCs and MPCs of Du145^TXR^ and MCF‐7^ADR^ cells. (R) Representative images and (S) quantification of ROS are shown. Student’s *t*‐test was used to analyze statistical differences. Mean with ± SD. Data information: Results are representative of three independent experiments. Source data are available online for this figure.

Previous studies have demonstrated that clathrin‐mediated endocytosis induced internalization of NPC1L1 when it functioned as a transporter (Ge *et al,* 2011; Li *et al,* 2014). To confirm this observation, immunofluorescence analysis revealed that combination treatment indeed significantly triggered the expression and internalization of NPC1L1 (Fig EV4A). Next, we examined total cellular cholesterol (CHOL) (a well‐known substrate of NPC1L1), and found marked accumulation of cholesterol in MPCs compared with MCs. By contrast, ezetimibe significantly induced the reduction in cholesterol in MPCs (Fig EV4B and C). We also performed vitamin E (a key substrate of NPC1L1; Narushima *et al,* 2008) analysis to evaluate whether vitamin E was increasingly absorbed by NPC1L1. Our data demonstrated that the content of vitamin E in MPCs medium was prominently decreased following exogenous addition of 50 μM vitamin E. In turn, ezetimibe blocked the uptake of vitamin E in MPCs (Fig 3H and I). Cholesterol and especially vitamin E which has a powerful anti‐oxidative ability by scavenging lipid ROS are inextricably linked with redox homeostasis (Singh *et al,* 2005; Kopecka *et al,* 2020). Thus, we hypothesized that NPC1L1 was involved in redox regulation in MPCs due to substrate absorption. To investigate our hypothesis, we treated MDR cancer cells with cholesterol or vitamin E to examine which substrate could rescue MDR cancer cells from triple‐combinatorial treatment‐elicited oxidative stress and cell death. Using BODIPY 581/591 C11 reagent, we detected the generation of lipid ROS in Du145^TXR^ and MCF‐7^ADR^ cells following triple‐combinatorial treatment with or without 50 μM cholesterol or 50 μM vitamin E by flow cytometry. We observed that ezetimibe treatment further elevated lipid ROS accumulation induced by combination therapy. Both vitamin E and cholesterol combined with triple‐combinatorial therapy alleviated lipid ROS in Du145^TXR^. In MCF‐7^ADR^ cells, vitamin E but not cholesterol led to decreased lipid ROS (Fig EV4D–F). In line with the results from ezetimibe, knockdown of NPC1L1 by si*NPC1L1* also resulted in a similar outcome (Fig EV4G–I). Furthermore, utilization of either ezetimibe or si*NPC1L1* significantly promoted malondialdehyde (MDA) levels (a specific product of lipid peroxidation) in combination therapy‐treated cells, while vitamin E alone compromised triple‐combinatorial treatment‐induced upregulation of MDA levels (Fig EV4J–M). In addition, cell viability and colony formation assays revealed that 50 μM vitamin E significantly rescued tri‐combinatorial treatment‐induced cell death. In contrast, 50 μM cholesterol showed only a modest effect (Fig 3J–M). Notably, we found chemotherapy agents/verapamil treatment further induced cell death of MDR cancer cells that were cultured in serum‐free medium where cholesterol and vitamin E were not present, compared with normal culture conditions (Fig 3J–K), suggesting that MDR cancer cells survive treatment with chemotherapy agents/verapamil due to absorption of vitamin E from serum by activating NPC1L1. Expectedly, *NPC1L1* knockdown induced a similar outcome to ezetimibe (Fig EV4N–Q). Moreover, we also measured ROS levels of MPCs and MCs, and found that the intracellular ROS levels of MPCs were slightly higher than those of MCs in both Du145^TXR^ and MCF‐7^ADR^ (Fig EV4R and S), implying that elevated expression of NPC1L1 likely protected from “damaging” species such as lipid peroxides, while the observation of higher ROS levels might support ROS‐mediated survival signaling in MPCs. Taken together, these data demonstrate that NPC1L1 plays a key role in maintaining redox homeostasis mainly by enhancing vitamin E absorption, preventing cell death from chemotherapy agents/verapamil treatment‐induced oxidative stress in a timely manner.

### NPC1L1 inhibitor ezetimibe induces lethal macropinocytosis in MPCs

Specific therapy targeting of MDR persister cells in the clinic may be applied by two alternative strategies: inhibition of NPC1L1 function by using ezetimibe or complete deprivation of vitamin E in cancer cells. The former is a readily available option. Following triple‐combinatorial treatment, surprisingly we observed a striking cytoplasmic vacuolization, which is not likely caused by autophagy (Movie EV1). To further exclude the role of autophagy, we first examined the expression of autophagy‐related proteins (Atg5 and LC3B) after chloroquine (CQ) treatment. As shown in Appendix Fig S4A, *Atg5*‐deficient Du145^TXR^ cells displayed a failed conversion of LC3B‐I to lipidated LC3B‐II (an established autophagosome marker) and a lack of Atg5 expression after CQ treatment which is consistent with previous studies (Ouyang *et al*, 2013). Therefore, we further investigated whether triple‐combinatorial treatment induced autophagy in MCF‐7^ADR^ cells. As shown in Appendix Fig S4B, triple‐combinatorial therapy resulted in marked autophagy induction, as evidenced by increased LC3B turnover and levels of Atg5 and Atg7. In contrast, an early autophagy inhibitor (3‐Methyladenine (3‐MA), 1 mM) prominently decreased LC3B lipidation, Atg5, and Atg7 expression in triple‐combinatorial therapy‐treated MCF‐7^ADR^ cells. In addition, to investigate whether autophagy was involved in the anti‐cancer effect of triple‐combinatorial therapy, Du145^TXR^ and MCF‐7^ADR^ cells were treated with triple‐combinatorial therapy combined with 3‐MA. As shown in Appendix Fig S4C, 3‐MA showed no obvious influence on cell viability. Consistently, no obvious change in clonogenic survival was observed following 3‐MA treatment in triple‐combinatorial therapy‐treated cancer cells (Appendix Fig S4D and E). Taken together, our results suggest that triple‐combinatorial treatment‐induced cell death may not be dependent on autophagy induction.

**Figure 4:**
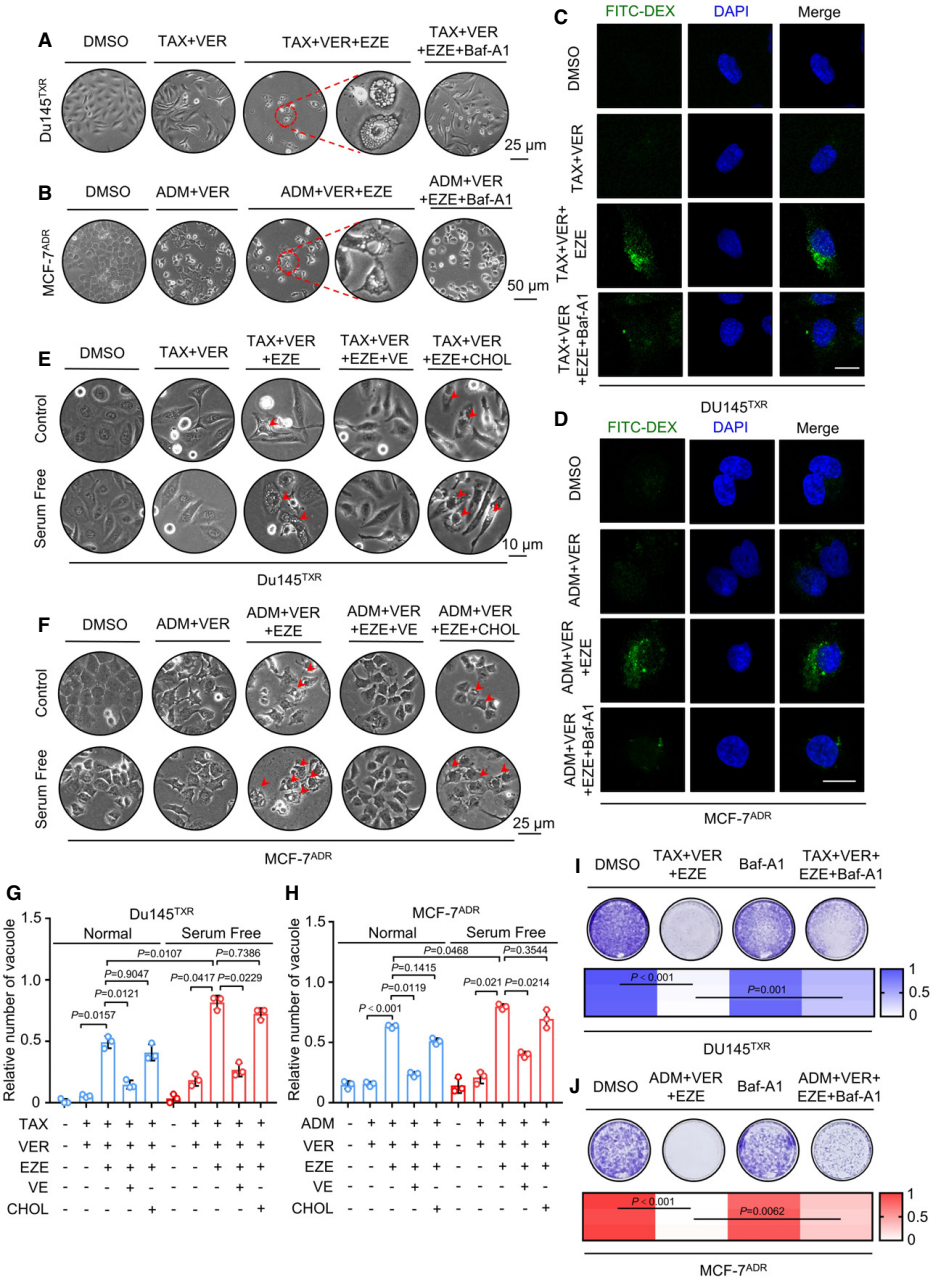
Utilization of NPC1L1 inhibitor ezetimibe induces lethal macropinocytosis in MPCs A, BPhase contrast images showing the morphological change in (A) Du145^TXR^ or (B) MCF‐7^ADR^ cells treated with indicated agents for 72 h. Scale bars, 25 or 50 μm (low‐magnification images).C, DEndocytosis analysis of (C) Du145^TXR^ or (D) MCF‐7^ADR^ cells treated with indicated agents for 8 h, followed by staining with 250 μg/ml FITC‐DEX for 4 h. Scale bars, 10 μm.E, FPhase contrast images of the morphological changes in (E) Du145^TXR^ and (F) MCF‐7^ADR^ cells cultured in normal or serum‐free medium followed by treatment with indicated agents for 12 h. Scale bars, 10 or 25 μm. The red arrows indicate vacuoles.G, HQuantification of vacuoles in E‐F. One‐way ANOVA was used to analyze statistical differences. Mean with ± SD.I, JColony formation assay and quantification of (I) Du145^TXR^ or (J) MCF‐7^ADR^ cells treated with indicated agents. One‐way ANOVA was used to analyze statistical differences. Data information: Results are representative of three independent experiments. Source data are available online for this figure.

Subsequently, we paid our attention to macropinocytosis, which has previously been reported as an endocytic adaptive process in the context of various stress situations (Marques *et al,* 2017). Bafilomycin A1 (Baf‐A1), which plays an important role in inhibiting early‐ and late‐phase macropinocytosis (Yoshimori *et al,* 1991), was used in a preliminary investigation to understand the nature of induction of these vacuoles. Phase‐contrast microscopy indicated that treatment induced multiple large single membrane bounded empty vesicles, which were almost completely abrogated by Baf‐A1 (100 nM; Fig 4A and B). In addition, we observed an increased uptake of fluorescently labeled high molecular weight dextran (FITC‐dextran, a specific substrate of macropinocytosis) by immunofluorescence. Expectedly, co‐treatment of Baf‐A1 compromised this effect (Fig 4C and D). These results indicate that the NPC1L1 inhibitor ezetimibe induces macropinocytosis in MPCs.

Indeed, macropinocytosis, as a metabolic adaptation to nutrient stress, usually plays a protective role in cancer by promoting nutrient scavenging and anabolism. However, excessive activation of macropinocytosis is associated with targeting drug sensitization, such as enhancing the delivery of anti‐cancer drugs or inducing a catastrophic and rapid fluid uptake that induces cell death (Hussein *et al,* 2021). Accordingly, we next tried to understand the meaning of triple‐combinatorial treatment‐induced macropinocytosis for MDR cancer cells. We cultured MDR cancer cells in serum‐free medium, performed triple‐combinatorial treatment, and observed premature cytoplasmic vacuolization compared with normal culture conditions. NPC1L1 substrate vitamin E, rather than cholesterol, effectively decreased triple‐combinatorial treatment‐induced vacuoles (Fig 4E–H). Consistently, inhibition of macropinocytosis by Baf‐A1 significantly rescued triple‐combinatorial treatment‐induced cell death, as evidenced by increased cell viability and colony formation (Appendix Fig S4F and Fig 4I and J). These data indicate that triple‐combinatorial treatment induces lethal macropinocytosis in MDR cancer cells. Notably, in contrast to the potent cytotoxicity induced by triple‐combinatorial treatment, utilization of ezetimibe alone seemed to have only a modest effect after treatment with chemotherapeutic agents/verapamil treatment (Appendix Fig S4G and H), suggesting triple‐combination therapy as an effective strategy for targeting persister cells from MDR‐expressing cancer cells. Collectively, our results indicate that the NPC1L1 inhibitor ezetimibe leads to lethal macropinocytosis in MPCs.

### NRF2‐mediated transcription and decreased DNA methylation contribute to upregulation of NPC1L1 in MPCs

**Figure 5:**
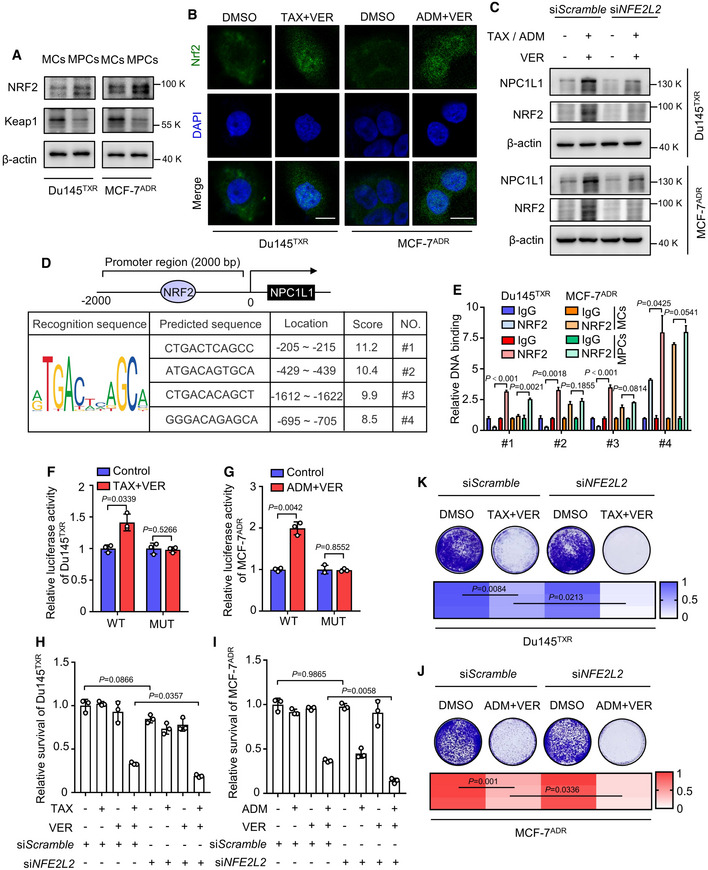
NRF2‐mediated transcription contributes to upregulation of NPC1L1 in MPCs AImmunoblotting of NRF2 and Keap1 in MCs and MPCs of Du145^TXR^ or MCF‐7^ADR^ cells.BImmunofluorescence analysis of NRF2 in Du145^TXR^ or MCF‐7^ADR^ cells treated with indicated agents for 12 h. Scale bar, 10 μm.CImmunoblot analysis of NPC1L1 and NRF2 in Du145^TXR^ or MCF‐7^ADR^ cells transfected with si*NFE2L2* or si*Scramble* followed by treatment with the indicated agents for 24 h.DThe profiles of predicted binding sequence of NRF2 in *NPC1L1* promotor by JASPAR.EChIP‐qPCR analysis of NRF2 binding on the *NPC1L1* proximal promoter in MCs and MPCs of Du145^TXR^ or MCF‐7^ADR^ cells. Student’s *t*‐test was used to analyze statistical differences. Mean with ± SD.F, GRelative luciferase activities of wild‐type (WT) or mutant (MUT) *NPC1L1* promoter (−205 to −215, #1) reporter constructs in (F) Du145^TXR^ or (G) MCF‐7^ADR^ cells treated with indicated agents. Student’s *t*‐test was used to analyze statistical differences. Mean with ± SD.H, ICell viability of (H) Du145^TXR^ or (I) MCF‐7^ADR^ cells transfected with si*NFE2L2* or si*Scramble* followed by treatment with the indicated agents for 72 h. One‐way ANOVA was used to analyze statistical differences. Mean with ± SD.J, KColony formation assay and quantification of (J) Du145^TXR^ or (K) MCF‐7^ADR^ cells transfected with si*NFE2L2* or si*Scramble* followed by treatment with the indicated agents. One‐way ANOVA was used to analyze statistical differences. Data information: Results are representative of three independent experiments. Source data are available online for this figure.

Given the massive involvement of oxidative stress, it was possible that NPC1L1 was transcriptionally regulated by antioxidant transcription factors (TFs). Nuclear factor‐erythroid 2 p45‐related factor 2 (NRF2), a principal TF contributing to oxidative stress defense, could provide an initial adaption to oxidative stress before the “flood gate” of ROS was overwhelmed (Hayes *et al,* 2020). To determine whether NRF2 contributed to transcriptional activation of NPC1L1 in MPCs, we firstly evaluated the expression of NRF2 and its negative regulator Keap1 by Western blotting and found increased NRF2 expression and decreased Keap1 in MPCs (Fig 5A). In addition, immunofluorescence analysis revealed nuclear NRF2 suggesting the activation of transcriptional function of NRF2 (Fig 5B). Knockdown of NRF2 by si*NFE2L2* significantly counteracted treatment‐induced upregulation of NPC1L1 (Fig 5C). To investigate the binding site of NRF2 interacting with the potential promoter region (2 kb upstream the transcriptional start site) of NPC1L1, the JASPAR database was used to predict transcription factor‐binding profiles. As shown in Fig 5D, scoring indicated that NRF2 could bind the promoter of NPC1L1 at four possible sites. ChIP assay revealed that predicted sequence #1 with the highest score was the binding region of NRF2 to NPC1L1 in both MPC cell lines (Fig 5E). This observation was further confirmed using luciferase reporter assays with either wild type (WT) or mutant (MUT), indicating that the predicted sequence #1 (−205 to −215 bp site on NPC1L1 promoter) was pivotal for the transcriptional activation of NPC1L1 (Fig 5F and G). As expected, siRNA‐mediated *NFE2L2* knockdown could significantly increase chemotherapeutic agents/verapamil‐induced growth suppression, as evidenced by decreased cell viability and colony formation (Fig 5H–K). Together, our data demonstrate that NRF2 is responsible for transcriptional upregulation of NPC1L1 in MPCs.

**Figure EV5:**
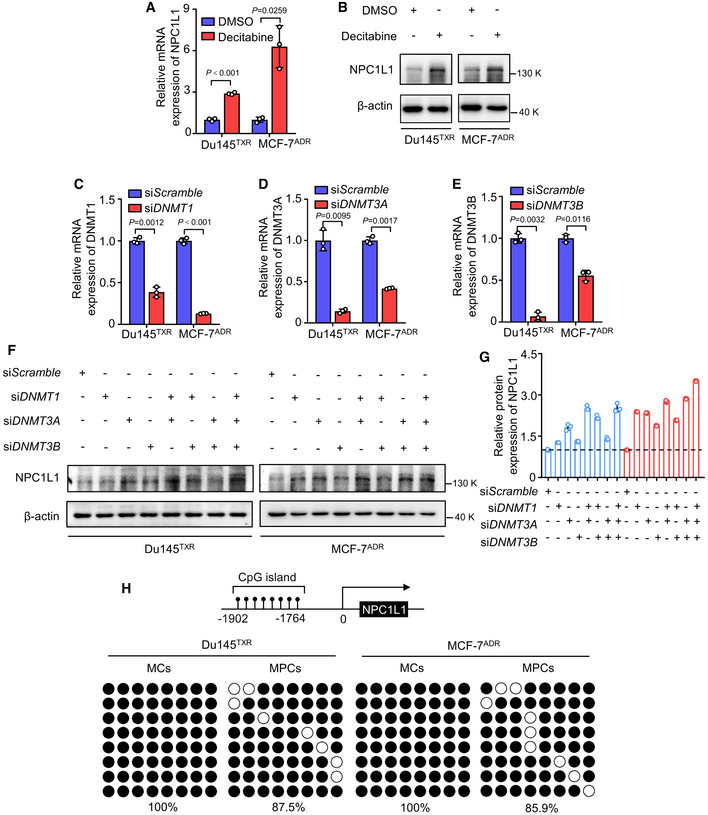
Decreased DNMTs‐mediated DNA methylation contributes to upregulation of NPC1L1 in MPCs AqRT‐PCR analysis of NPC1L1 in Du145^TXR^ or MCF‐7^ADR^ cells treated with 2 μM decitabine for 72 h. Student’s *t*‐test was used to analyze statistical differences. Mean with ± SD.BImmunoblotting of NPC1L1 in Du145^TXR^ or MCF‐7^ADR^ cells treated with 2 μM decitabine for 72 h.C–EqRT‐PCR analyses of (C) DNMT1, (D) DNMT3A, and (E) DNMT3B in Du145^TXR^ or MCF‐7^ADR^ cells transfected with si*DNMT1*/si*DNMT3A*/si*DNMT3B* or siScramble. Student’s *t*‐test was used to analyze statistical differences. Mean with ± SD.F‐G(F) Immunoblotting of NPC1L1 in Du145^TXR^ or MCF‐7^ADR^ cells transfected with si*DNMT1*/si*DNMT3A*/si*DNMT3B* or si*Scramble*. (G) The expression levels of NPC1L1 in (F) were quantified. Dotted lines represent value 1 (relative protein expression of NPC1L1). Mean with ± SD.HBisulfite genomic sequencing of the methylation level of eight CpGs within CpG island of NPC1L1 promotor in MCs and MPCs of Du145^TXR^ or MCF‐7^ADR^ cells. The black circles and empty circles indicate methylated and unmethylated CpG dinucleotides, respectively. Data information: Results are representative of three independent experiments. Source data are available online for this figure.

In addition, abnormal expression of NPC1L1 has been reported to be associated with extensive hypomethylation (Malhotra *et al,* 2014; Nicolle *et al,* 2017). Notably, epigenetic alterations are a common regulatory process for enabling persister cells to acquire the reversible phenotype (Shen *et al*, 2019, 2020). Accordingly, we further investigated whether the changes in DNA promoter methylation of NPC1L1 were involved in its upregulation. As shown in Fig EV5A and B, mRNA or protein levels of NPC1L1 were relatively lower in the MCs, and treatment with the DNA methyltransferase inhibitor decitabine clearly increased NPC1L1 mRNA or protein expression, suggesting that hypermethylation within the gene promoter of *NPC1L1* mediated its transcriptional inhibition in MCs. To further confirm this conclusion, siRNA‐mediated DNA methyltransferases silencing of *DNMT1*, *DNMT3A*, and *DNMT3B* was utilized to examine the effect of DNA methylation silencing on NPC1L1 expression (Fig EV5C–E). As expected, the protein expression of NPC1L1 was significantly increased following knockdown of DNA methyltransferases (Fig EV5F–G), indicating that NPC1L1 expression in MPCs resulted from decreased involvement of DNMT1, DNMT3A, and DNMT3B‐regulated DNA methylation. To identify whether CpG sites were methylated, we predicted the CpG island of the promoter region of NPC1L1 (2 kb upstream from the transcriptional start site) using MethPrimer, then sequenced the predicted CpG island at −1,764 to −1,902 bp site by bisulfite sequencing. We found that eight CpGs were hypermethylated in MCs, while in persister Du145^TXR^ cells or persister MCF‐7^ADR^ cells, the percentage of methylated CpGs decreased from 100% to 87.5% or to 85.9%, respectively (Fig EV5H). Taken together, these results demonstrate that decreased DNA methylation may contribute to the upregulation of NPC1L1, at least in part, in MPCs.

### Ezetimibe synergizes with nanocarrier‐based chemotherapy to eradicate MDR cancer cells and prevent recurrence *in vivo*

It seemed likely that application of the triple‐combinatorial treatment, chemotherapeutic agents/verapamil/ezetimibe, could potentially be performed in a clinical setting to eradicate MDR cancer cells and prevent tumor relapse. However, the limitations of verapamil, including adverse side effects and low bioavailability (Dong *et al,* 2020), have been widely noted. It was therefore necessary to solve this problem before testing this treatment regime in a preclinical model. We chose a zeolitic imidazolate framework ZIF‐8, a type of the biocompatible metal organic framework (MOF; Zhang *et al*, 2017, 2019, 2020), as a carrier for efficient delivery of verapamil, which was then decorated with electronegative hyaluronic acid (HA) shells to enhance the active targeting of VER@ZIF‐8 (Appendix Fig S5A). As shown in Appendix Fig S5B, transmission electron microscopy (TEM) imaging revealed that both VER@ZIF‐8 and HA/VER@ZIF‐8 showed a uniform spherical‐like morphology. Furthermore, the particle sizes were found to be 196.4 nm for VER@ZIF‐8 and 210.6 nm for HA/VER@ZIF‐8 with a narrow distribution, demonstrating a suitable nanoparticle size for passive tumor targeting (Appendix Fig S5C). The zeta potential of VER@ZIF‐8 and HA/VER@ZIF‐8 was characterized to be 21.3 mV and −24.1 mV, respectively, suggesting the successful adsorption of HA (Appendix Fig S5D).

**Figure 6:**
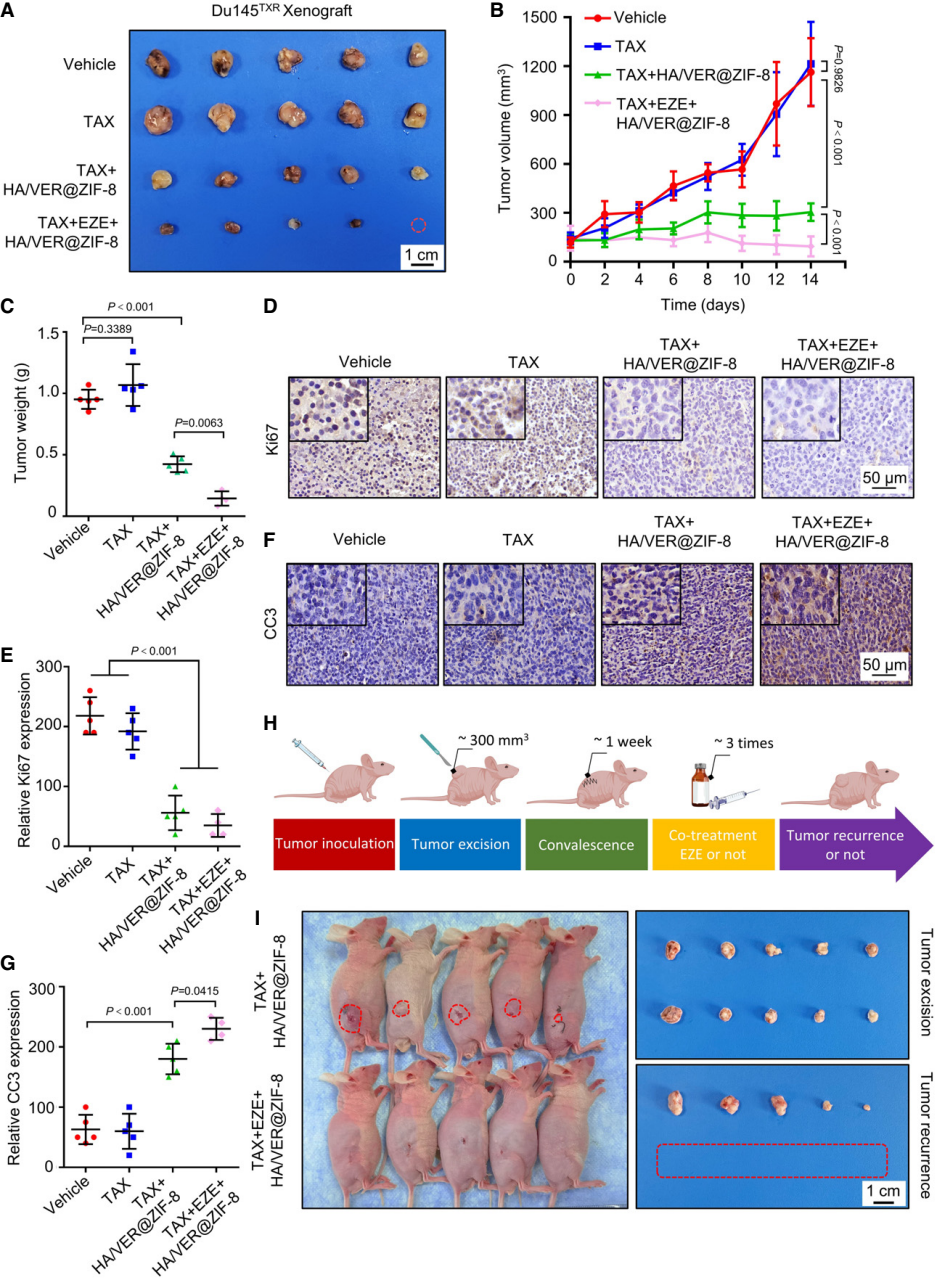
Ezetimibe synergizes with chemotherapy to eradicate MDR cancer cells and prevent recurrence *in vivo* A–CDu145^TXR^‐derived xenograft male nude mice received the indicated treatments. (A) Images, (B) tumor volumes, and (C) weights measured at the indicated time points are shown. Scale bar, 1 cm. The red dotted line indicates cured tumors. Two‐way ANOVA was used to analyze statistical differences in (B). One‐way ANOVA was used to analyze statistical differences in (C). Mean with ± SD.D, EImmunohistochemical staining of Ki67 in indicated groups. (D) Representative images and (E) relative immunohistochemical scores are shown. Scale bar, 50 μm. Student’s *t*‐test was used to analyze statistical differences. Mean with ± SD.F, GImmunohistochemical staining of cleaved caspase 3 (CC3) in indicated groups. (F) Representative images and (G) relative immunohistochemical scores are shown. Scale bar, 50 μm. One‐way ANOVA was used to analyze statistical differences. Mean with ± SD.H, I(H) Schematic of tumor recurrence model establishment and subsequent treatment. (I) Corresponding images and isolated tumors are shown. The red dotted line represents cured tumors. Data information: In B, C, E and G, data were obtained from 4 to 5 tumors per group. Source data are available online for this figure.

We next generated a mouse xenograft model by subcutaneously inoculating Du145^TXR^ cells to explore the anti‐cancer effects of triple‐combinatorial treatment of TAX/(HA/VER@ZIF‐8)/ezetimibe *in vivo*. As shown in Fig 6A–C, TAX/(HA/VER@ZIF‐8) treatment displayed an obvious reduction in the size, growth rate, and weight of xenograft tumors in comparison with the vehicle group and TAX treatment alone. As expected, the triple‐combinatorial treatment of TAX/(HA/VER@ZIF‐8)/ezetimibe exhibited a further decline in xenograft tumor size, growth rate, and weight compared with TAX/(HA/VER@ZIF‐8) treatment. In addition, TAX/(HA/VER@ZIF‐8) treatment and the triple‐combinatorial treatment of TAX/(HA/VER@ZIF‐8)/ezetimibe in Du145^TXR^ xenograft tumors displayed reduction in Ki67 staining compared with the vehicle and TAX monotherapy groups (Fig 6D and E). We further examined the cleaved caspase 3 (CC3) staining of xenograft tumors from four groups. As shown in Fig 6F and G, TAX/(HA/VER@ZIF‐8) treatment in Du145^TXR^ xenograft tumors showed stronger CC3 staining in comparison with the vehicle group or TAX monotherapy group, and the triple‐combinatorial treatment of TAX/(HA/VER@ZIF‐8)/ezetimibe showed a further upregulation of CC3 staining. Furthermore, obvious oxidative damage was observed in tumors from TAX/(HA/VER@ZIF‐8) treatment group as evidenced by increased 8‐OHdG (a well‐known oxidative stress marker) intensity, while triple‐combinatorial treatment displayed a more robust effect (Appendix Fig S5E and F). We also observed increased expression of NPC1L1 following TAX/(HA/VER@ZIF‐8) treatment by immunohistochemical staining consistent with the *in vitro* results, suggesting the occurrence of a DTP state in MDR cancer cells (Appendix Fig S5G and H). The morphology of major organs from the mice was examined by staining with hematoxylin and eosin (H&E). As shown in Appendix Fig S5I, the results of H&E staining indicated the no obvious toxic effects in the studied groups.

In light of the importance of DTPs in tumor relapse, we adopted a tumor recurrence model to mimic minimal residual disease in cancer patients. Specifically, we surgically removed the tumors when their average volume reached ~250 mm^3^ following subcutaneous injection of Du145^TXR^ cells into mice which were then randomized into two groups. After a postoperative recovery of 1 week, the mice were treated once every 2 days with TAX/(HA/VER@ZIF‐8) or TAX/(HA/VER@ZIF‐8)/ezetimibe (three treatments), to determine whether TAX/(HA/VER@ZIF‐8)/ezetimibe treatment was able to effectively prevent tumor relapse (Fig 6H). Surprisingly, our results revealed that TAX/(HA/VER@ZIF‐8) treatment had no obvious effect on tumor recurrence, although it had shown a curative effect for the tumor. By contrast, TAX/(HA/VER@ZIF‐8)/ezetimibe treatment showed a marked inhibition of both tumor growth and tumor recurrence (Fig 6I). Collectively, our data reveal that this particular triple‐combinatorial treatment contributes to a dominant growth suppression of MDR cancer and an attenuation of tumor relapse with no obvious side effects.

## Discussion

The complexity underlying failure of chemotherapy arises from various mechanisms, although much is generally attributed to the engagement of drug transporters that modulate the concentration of agents under a certain threshold to facilitate cell adaptation (Alexa‐Stratulat *et al,* 2019; Dong *et al,* 2020). Accordingly, the large number of studies targeting MDR cancer cells by modulation of drug transporters is justified. However, recent reports propose that cancer cells may exist in a free‐shuttling continuum that is a transient non‐mutational phenotype (so‐called drug‐tolerant persister (DTP)), to adapt to different pressures such as chemotherapy and targeted therapy, rather than as a discrete pool with unchanging factor‐induced drug resistance (Shen *et al*, 2019, 2020). This alters our understanding of resistance and the design of new chemotherapy regimens. It is known that this special subpopulation can survive or even expand in the presence of the drug after long‐term therapy. Moreover, a subgroup may further acquire a mutation‐driven resistance mechanism, thus becoming progressively drug tolerant or eventually drug resistant (Shen *et al*, 2019, 2020; Chen *et al,* 2021). Here, we observed the occurrence of a DTP state during routine treatment (combination chemotherapeutic agents with MDR1 inhibitors verapamil) in MDR cancer cells with aberrant expression of MDR1, highlighting that targeting cancer persistence also needs to be taken into consideration for breaking the “glass ceiling” of MDR.

Growing understanding of the molecular mechanisms governing the DTP state has driven researchers to explore the actionable drug targets in cancer combination treatment. As expected, persister cancer cells, that usually arise after oncotherapy, possess a powerful anti‐oxidant system against oxidative stress in the harsh environment created by anti‐cancer drugs (Shen *et al*, 2019, 2020; Dhimolea *et al,* 2021; Oren *et al,* 2021; Rehman *et al,* 2021). In addition, these cells are associated with a ubiquitous overproduction of reactive oxygen species (ROS), such as superoxide, resulting from enhanced mitochondrial oxidative respiration (Anand *et al,* 2019). Therefore, a redox‐dependent therapy which weakens reductive capacity while increasing oxidative potential should overwhelm the antioxidant program of persister cells and thus lead to cell death. For example, Dhimolea *et al* (2021). revealed that Myc inactivation‐mediated embryonic diapause‐like adaptation supports cancer cell survival via reduced redox stress (Dhimolea *et al,* 2021). In this study, aiming to preferentially repurpose existing drugs for a potential clinical strategy (Zhang *et al*, 2017, 2019, 2020), we screened the DEGs between MDR persister cancer cells and MDR cancer cells using RNA‐seq. This revealed NPC1L1, the direct target of the clinical hypolipidemic drug ezetimibe, as a key oxidative stress effector expressed highly in MDR persister cancer cells, contributing to enhanced uptake of vitamin E to partially counteract the combinatorial therapy‐induced oxidative stress. In addition, NRF2, a well‐known oxidant defense transcription factor, was revealed to directly regulate NPC1L1 expression by binding to the −205 to −215 bp site on the NPC1L1 promoter, thus providing a ROS‐neutralizing mechanism for protecting persister cells from oxidative stress. Consistent with our finding, a previous study which focused on TRPA1, a neuronal redox‐sensing channel, has demonstrated a novel oxidative stress tolerance mechanism, distinct from classical mechanisms, against excessive accumulation of ROS, promoting Ca^2+^‐dependent anti‐apoptotic signaling and eventually leading to chemotherapy resistance (Takahashi *et al,* 2018). Furthermore, Nisebita *et al*. showed that the miR‐371‐3p/peroxiredoxin 6 axis contributed to drug tolerance by modulating redox homeostasis, suggesting combining current cancer therapies with a ROS inducer as a potential strategy for delaying acquired drug resistance (Sahu *et al,* 2016). In this context, our study provides a NPC1L1‐mediated new perspective regarding a non‐canonical anti‐oxidative mechanism. Notably, utilization of ezetimibe during combination therapy markedly induced lethal macropinocytosis, resulting in oxidative stress‐dependent cell death. Although the specific mechanism of macropinocytosis induction in this process needed to be further elucidated, we propose a novel concept whereby co‐treatment of MDR cancer cells with routine anti‐MDR therapy together with the NPC1L1 inhibitor ezetimibe significantly reduces the residual persister cell pool by inducing oxidative stress‐dependent lethal macropinocytosis.

In addition to the dependence of orchestrated anti‐oxidative signaling networks, these persister cells hold well‐characterized reversibility of their cell status, which is mainly sustained by epigenetic pathways that do not directly lead to oncogenic mutations (Shen *et al*, 2019, 2020). In support of the epigenetic mechanism, alterations of histone methylation or acetylation and DNA or mRNA methylation (Recasens & Munoz, 2019; Shen *et al*, 2019, 2020) are usually found in DTP cells. For example, one study revealed that persister cells in melanoma highly expressed KDM5, thereby inducing H3K4me3/2 demethylation‐mediated cell cycle arrest (Roesch *et al,* 2010). Furthermore, persister cancer cells able to tolerate BRAF and MEK inhibitors performed a reversible translation reprogramming which benefited survival, resulting from an increased N6‐methyladenosine (m6A) modification in the untranslated regions of a subset of mRNAs (Shen *et al*, 2019, 2020). Indeed, our study identified an epigenetic process regarding expression of NPC1L1 that was crucial for MDR persister cells, mediated to an extent by DNA methyltransferases (DNMTs). Notably, NPC1L1 generally kept in a DNA methylation‐induced epigenetic silencing state is highly expressed in intestine due to extensive hypomethylation in order to facilitate cholesterol uptake (Dimova *et al,* 2017). In the DTP state, we confirmed the epigenetic regulation of NPC1L1 by DNA demethylation in MDR cancer cells, similar to intestinal cells, which mainly contributed to vitamin E uptake against oxidative stress. As summarized for epigenetic regulation in the DTP state, previous studies have identified numerous potential anti‐persister strategies by pharmacologically targeting the epigenetic enzymes KDMs, HDACs, and BET (Vinogradova *et al,* 2016; Risom *et al,* 2018; Wang *et al,* 2018a,b). However, killing DTP cells might require a highly personalized approach. Epigenetic drugs, including GSK‐J4‐ and JQ1‐targeting KDM and BET, respectively, are non‐selective inhibitors. A broader and deeper understanding of epigenetic enzymes‐modulated signaling networks and development of novel approach targeting the downstream effectors is therefore required. Unlike these previous strategies, our study highlights a potential clinical application by targeting NPC1L1 in MDR cancer cells with a DTP state.

Here, we have provided key insights into the relationships among MDR, DTP state, and oxidative stress defense. Our data indicate that MDR cancer cells undergoing a chemotherapy attack can transform into a DTP state. Notably, MDR cancer cells appeared to show superiority compared to control cancer cells, manifesting faster entry into the DTP state and more long‐lasting tolerance to treatment. The use of RNA‐seq to determine what supports cell survival in response to cytotoxic stress identified NPC1L1 as a potential druggable target in MDR persister cells. Further data suggested that NPC1L1 enhanced uptake of vitamin E to partially counter chemotherapy‐induced oxidative stress, whereas inhibition of NPC1L1 function using ezetimibe could trigger oxidative stress‐dependent cell death through activation of lethal macropinocytosis. In this regard, NPC1L1 was shown to be upregulated directly by NRF2‐mediated transcription, with decreased DNA methylation partially contributing to this process. Using a commonly available nanoparticle delivery system to mitigate the side effects of verapamil, we identified the triple‐combination strategy of chemotherapeutic agents, verapamil, and ezetimibe, which showed an obvious anti‐tumor effect and capacity to prevent tumor recurrence *in vivo*.

Our work highlights a conceptual extension to the understanding of drug resistance and further confirms that oxidative stress defense programs represent a vulnerability of persister cancer cells. Therefore, the development of therapies targeting oxidative stress defense represents an attractive strategy to kill MDR cancer cells based on existing therapies. However, translation of the inhibitors of NRF2 or GPX4 into clinical application is not straightforward due to their non‐selective effects and limited bioavailability. In contrast, the NPC1L1 inhibitor ezetimibe is currently registered for treating hyperlipidemia in the clinic, and possesses potential for drug repurposing in oncology therapeutic areas. Our results support the potential clinical value of targeting oxidative stress defense in MDR cancer cells.

## Materials and Methods

### Cell lines and cell culture

Human cancer cell line Du145/Du145^TXR^ and MCF‐7/MCF‐7^ADR^ cells were kindly provided by Prof. Jian Zhang (School of Medicine, Southern University of Science and Technology Shenzhen) and Prof. Jian Zhang (State Key Laboratory of Cancer Biology, Fourth Military Medical University), respectively. Du145/Du145^TXR^ cells or MCF‐7/MCF‐7^ADR^ cells were, respectively, cultured in RPMI Medium 1640 (Thermo Fisher Scientific, 31870082 A1048901) or in Minimum Essential Medium (Thermo Fisher Scientific, A1048901), which was supplemented with 10% FBS (HyClone, SH30088.03), and 100 U/ml penicillin and streptomycin (Invitrogen). All cells were maintained in a humidified atmosphere with 5% CO_2_ at 37°C. All cell lines were recently authenticated by STR profiling. In addition, only non‐contaminated cell lines were used for experiments.

### Antibodies and reagents

Product code and suppliers for all commercial antibodies used in this study are shown in Table 1. The dilution ratios of antibodies used in immunoblotting, immunofluorescence, and immunohistochemistry are 1:1,000, 1:200, and 1:100, respectively.

**Table 1:** Primary antibodies in this study.

Antibody	Company and Cat. No.
MDR1	#13342S, Cell Signaling Technology
p21	#2947, Cell Signaling Technology
p27	#3686, Cell Signaling Technology
ALDH1A1	#36671, Cell Signaling Technology
Oct‐4A	#2840, Cell Signaling Technology
Sox2	#3579, Cell Signaling Technology
KLF4	#4038, Cell Signaling Technology
CD44	#3570, Cell Signaling Technology
E‐cadherin	#3195, Cell Signaling Technology
Vimentin	ab8978, Abcam
NPC1L1	NB400‐127, NOVUS
NRF2	sc‐518033, Santa Cruz Biotechnology
Keap1	sc‐365626, Santa Cruz Biotechnology
8‐OHdG	sc‐393871, Santa Cruz Biotechnology
β‐actin	sc‐8432, Santa Cruz Biotechnology

Product code and suppliers for all commercial chemical reagents used in this study are listed in Table 2. All chemicals were handled according to the supplier’s recommendations.

**Table 2:** Chemical reagents in this study.

Chemical reagent	Company and Cat No.
Taxol	S1150, Selleck
Adriamycin HCl	S1208, Selleck
Verapamil HCl	S4202, Selleck
Rhodamine 123	ab275545, Abcam
3‐Methyladenine	HY‐19312, MedChemExpress
NAC	S1623, Selleck
Z‐VAD(OMe)‐FMK	HY‐16658, MedChemExpress
Ezetimibe	S1655, Selleck
Cholesterol	S4154, Selleck
Alpha‐Vitamin E	S4686, Selleck
Bafilomycin A1	S1413, Selleck
FITC‐DEX	P35368, Thermo Fisher Scientific
Decitabine	S1200, Selleck
Hyaluronic acid	9004‐61‐9, Aladdin
2‐methylimidazole	Beijing J & K Technology Co., Ltd
Zn(NO_3_)_2_·6H_2_O	Beijing J & K Technology Co., Ltd

### The generation of DTPs

For the control cell lines, persister cells were derived from the treatment of Du145 or MCF‐7 with 20 nM taxol or 200 nM adriamycin, respectively, for at least 9 days with fresh drug‐containing medium. For drug‐resistant cancer cells, persister cells were derived by co‐treatment of Du145^TXR^ or MCF‐7^ADR^ with 50 μM verapamil and 20 nM taxol or 200 nM adriamycin, respectively, for 3 days. In general, 70–90% of cells are killed and detached by the prescribed treatment. Surviving attached cells that are subsequently defined as persister cancer cells are trypsinized and washed once in phosphate‐buffered saline (PBS) for follow‐up exploration.

### Detection of cell growth and cell morphology

Cell sensitivity to the treatments was determined using a WST‐1 cell viability assay. Cells were plated in 96‐well plates (5,000 cells per well) and subjected to different treatments as reported previously (Shen *et al*, 2019, 2020). For the colony formation assay, cells were cultured in 24‐well plates with different treatments for 10 days. Giemsa was used to counterstain the colonies, then washed cells three times with cold PBS. Molecular Imager Gel Do XR^+^ System (BIO‐RAD) and Image J software (NIH), respectively, were used to record or count the visible colonies. For soft agar colony formation assay, 1 ml of 1% agar‐mixed medium and 1 ml of 0.5% agar‐mixed medium with 1 × 10^3^ cells were successively added to the 12‐well plates. Cells were cultured for 2–3 weeks at 37°C, and were observed and captured using a phase contrast microscope (Olympus IX73). For observation of cell morphology, cells were cultured in six‐well plates and treated with different agents or vehicle. Cell morphology was observed and captured using a phase contrast microscope (Olympus IX73).

### Western blot analysis

Cells cultured in six‐well plates were lysed in RIPA lysis buffer followed by a rinse with precooled PBS. RIPA lysis buffer comprised 150 mM Tris‐HCl, 150 mM NaCl, 1% NP‐40, 0.1% SDS, and protease inhibitors. Cell lysates were quantified by BCA Protein Assay (Thermo Fisher Scientific, 23250). Equal amounts of soluble proteins were separated by SDS‐PAGE. Proteins were transferred to PVDF membrane (EMD Millipore, ISEQ00010) and blocked with 5% powdered milk. The membranes were incubated overnight at 4°C with antibodies diluted at 1:1,000. After washing with TBST buffer, HRP secondary antibodies (Thermo Fisher Scientific) were used to incubate the membranes for immunoblot analysis.

### Immunofluorescence

5 × 10^3^ cells were cultured on glass coverslips overnight in 24‐well plates. Following fixation with 4% paraformaldehyde (Sigma), cells were permeabilized with 0.4% Triton X‐100 and blocked with 5% fetal bovine serum. Cells were then washed with PBS (3 times), followed by incubation with primary and Alexa Flor secondary antibodies. A Zeiss LSM 510 confocal microscope was used for immunofluorescence analysis. For FITC‐DEX staining, live cells were stained with 250 μg/ml FITC‐DEX for 4 h after 8 h of drug treatment at 37°C

### Immunohistochemistry

Immunohistochemistry was performed as previously described (Zhang *et al*, 2017, 2019, 2020). Samples were observed with the EnVision Detection System (Agilent Technologies, K5007). The quantitative score was calculated using the following equation: H‐score (< 300%) = A × B, A representing the percentage of staining‐positive cells area, and B representing the immunostaining intensity (0, negative; 1, weak; 2, positive; and 3, strongly positive).

### siRNA transfection

Negative control siRNA (si*Scramble*) sequence and the target sequences of si*ABCB1*, si*NPC1L1*, si*DNMT1*, si*DNMT3A*, s*iDNMT3B,* and si*NFE2L2* were chemically synthesized by GenePharma (Shanghai, China). The detailed sequences are shown in Table 3.

**Table 3:** List of siRNA sequences used for knockdown of *ABCB1*, *NPC1L1*, *DNMT1*, *DNMT3A*, *DNMT3B,* and *NFE2L2*, respectively.

siRNA	Oligomers (5′–3′)
ABCB1#1	AAGCGAAGCAGTGGTTCAGGT
ABCB1#2	GGGTAAAGTCAGTGATAAA
NPC1L1#1	CCAGCUACAUUGUCAUAUUTT
NPC1L1#2	CCGGTCCAGCTACAGGTAT
DNMT1	CAGAACAAGAAUCGCAUCUUU
DNMT3A	CUUUGAUGGAAUCGCUACAUU
DNMT3B	GGAUGCUAUUGUGAAUGUGTT
NFE2L2	AAAGUGAUAGAUCAGAAACAUCAAUGG
Negative control	UUCUCCGAACGUGUCACGUTT

All siRNAs were transfected with Lipofectamine 3000 reagent (Thermo Fisher Scientific) for 48 h according to the manufacturers’ protocol.

### Reactive oxygen species (ROS) and Lipid ROS assay

Measurement of intracellular ROS or lipid ROS levels were obtained using a ROS Assay Kit (S0033, Beyotime, China) or the BODIPY 581/591 C11 reagent (Invitrogen D3861) according to the manufacturer’s protocol, respectively. 1 × 10^4^ cells were cultured in 24‐well plates and treated with designated reagents for varying times. Cells were then stained with DCFH‐DA or BODIPY 581/591 C11 at 37°C for 30 min. Subsequently, cells were trypsinized and washed with PBS (3 times), and then were analyzed using flow cytometry (BD, FACS Celesta).

### Cellular total cholesterol and vitamin E analysis

Cells were cultured in six‐well plates and collected after treatment. The collected cells were washed with PBS (three times) and then cellular total cholesterol assay kits (Applygen Technologies Inc., Beijing, China) were used to measure total cholesterol content. For vitamin E analysis, cells were previously treated with 50 μm vitamin E and then treated with the indicated drug treatment. Vitamin E assay kits (Cat. No. SNM197), purchased from Beijing BIORAB, were used to measure the vitamin E content of collected supernatants. The experimental procedures were in accordance with the manufacturer’s instructions.

### GSH and GSSG measurement and malondialdehyde (MDA) levels analysis

Cells were cultured in six‐well plates and collected after treatment. Cellular glutathione (GSH) and oxidized glutathione (GSSG) were measured using a GSH or GSSG Assay kit (S0053, Beyotime, China) and the ratio of GSSG to GSH was then calculated according to the manufacturer’s protocol. The relative MDA content was examined using a lipid peroxidation MDA assay kit (S0131S, Beyotime, China) according to the manufacturer’s protocol.

### RNA‐seq and quantitative real‐time PCR

For RNA‐seq, total RNAs were extracted by TRIzol Reagent (Thermo Fisher Scientific, 15596018) following treatment. Subsequently, RNA samples were sent to Novogene Technology Co., Ltd. (Beijing, P.R. China), for high‐throughput sequencing analysis. For quantitative real‐time PCR, cDNA from extracted RNA was generated using TaKaRa PrimeScript RT Reagent kit (Takara, RR014) according to the indicated protocol. The expression of candidate mRNA was examined in a CFX Connect Real‐Time PCR Detection System (Bio‐ Rad). Primer sequences are listed in Table 4.

**Table 4:** List of primers used for quantification of specific gene expression.

Gene	Forward (5′–3′)	Reverse (5′–3′)
NPC1L1	CCAAGTCGACTGGAAGGACC	AGGGCCTCTGCCTCAGAATA
DNMT1	ATGCTTACAACCGGGAAGTG	TGAACGCTTAGCCTCTCCAT
DNMT3A	ACCACGACCAGGAATTTGAC	ACACCTCCGAGGCAATGTAG
DNMT3B	AGATCAAGCTCGCGACTCTC	CTGGGCTTTCTGAACGAGTC
β‐actin	AGGCCAACCGCGAGAAGATG	GCCAGAGGCGTACAGGGATA

### DNA methylation analysis

Gene JET Genomic DNA Purification kit (Thermo Fisher Scientific) was used to isolate genomic DNA from MDR cells or MDR persister cells. Then, the genomic DNA was subjected to bisulfite conversion using the EZ DNA Methylation‐Gold kit (Zymo Research). After PCR using TaKaRa EpiTaq HS DNA polymerase to amplify these bisulfite‐modified DNAs, TOPO TA Cloning kit (Thermo Fisher Scientific) was used to subclone the PCR products into the pCR4 TOPO vector for further analysis.

### Chromatin immunoprecipitation assays

Based on the predicted results from JASPAR (http://jaspar.genereg.net/), ChIP assay was used to confirm the binding region of NRF2 on the *NPC1L1* promoter. In brief, 1 × 10^7^ Du145^TXR^ or MCF‐7^ADR^ cells were treated with drugs and cross‐linked with 1% formaldehyde. Next, the samples were sonicated at 30% of the maximum power for ~8 min/10 s each pulse (XINYI‐IID). Supernatants were obtained by centrifugation at 15,000 × *g* for 10 min and immunoprecipitated with 1 μg of NRF2 antibody or IgG antibody using a ChIP kit (Millipore Corp.). The products were analyzed by RT‐qPCR to detect any DNA binding by NRF2. The primers used are shown in Table 5.

**Table 5:** List of primers of NPC1L1 promoter region used for ChIP assays.

Code	Forward (5′–3′)	Reverse (5′–3′)
1	GGTCCCATCTGTGCCTCCAGGCCCT	GCCCCCGCTTTAGATGAGCCTCATC
2	GTCACTGCGTCACTCCACCCTGCCT	GGCTCAGCCTCAGACCCCAGCTCTG
3	CCAGGGGCTTAGCCTAGACAGCCCC	GGCACCTTCAGTGTCAAAGGGCCTT
4	CAGGAGGGAGCAGAAAGTGTGTACC	CTTCATCCCCTCTTCCCCCCGACCT

### Dual‐luciferase reporter assay

The binding region of *NPC1L1* confirmed by ChIP assay was inserted into the pGL3 basic vector (Promega) for luciferase reporter assay according to the manufacturer’s protocol (Dual‐Luciferase Reporter Assay System Promega, E1910), and Renilla luciferase activity was used to normalize the transfection efficiency. The primers used for the constructions of wild type or mutants are listed in Table 6.

**Table 6:** List of primers for constructs in dual‐luciferase assays.

Constructs	Forward (5′–3′)	Reverse (5′–3′)
WT	GAGCTAGCcacgcgttctgtggaccataac	GCAAGCTTgctgccttaatgtgtgttcagcc
Mutant	cccctagctgactcagAcctctggcttc	Tctgagtcagctaggggagagaccacg

### Preparation and characterization of nanoparticles

For the preparation of HA/VER@ZIF‐8, 1 g 2‐methylimidazole (2‐mim) and 0.1 g Zn(NO_3_)_2_·6H_2_O, respectively, were dissolved in 4 ml and 400 μl ultrapure water. A 2 ml stock solution of 60 mg VER was added to Zn(NO_3_)_2_ stock solution with stirring for 5 min and then the mixed solution was added drop wise to the 2‐mim stock solution with stirring for 10 min. The samples were washed with ultrapure water (3 times). After vacuum drying for 3 days, VER@ZIF‐8 was obtained. VER@ZIF‐8 dispersed in ultrapure water was prepared for further modification with HA on the surface of VER@ZIF‐8. In brief, VER@ZIF‐8 stock solution and 3 mg/ml HA solution were stirred for 3 days. HA/VER@ZIF‐8 was obtained by vacuum drying. Redispersed samples were prepared for transmission electron microscopy (TEM) analysis (JEM‐200CX transmission electron microscope). The average size and distribution of nanoparticles were examined by dynamic light scattering analysis (Brookhaven BI‐200SM). A Zetasizer Nano ZS90 (Malvern, Westborough, MA) was used for detecting the zeta potential of the nanoparticles. A standard curve of VER (y = 0.0059x + 0.0106, *R*² = 0.9994) was confirmed by UV‐vis spectrophotometry to calculate the VER content of HA/VER@ZIF‐8.

### Animal studies

All animal experiments were approved by the Institutional Animal Care and Use Committee of Sichuan University. All male BALB/c nude (CAnN. Cg‐Foxn1nu/Crl) mice (7‐week old) were obtained from HFK Bioscience Co., Ltd (Beijing, China). All mice were housed in cages contained aspen chip bedding in rooms under standard condition (temperature: 20–22°C, relative humidity: 61–65%, 12 h light/dark cycle, and 5 mice per cage) in accordance with institutional guidelines from Animal Care and Use Committee of Sichuan University. Mice had free access to tap water and standard commercial mouse chow (HFK Bioscience Co., Ltd, Beijing, China). For the subcutaneous xenograft model, Du145^TXR^ cells (1 × 10^7^ cells/mouse) were suspended in cold PBS and then subcutaneously administrated to the rear flank of the mice. The tumor size was measured using digital calipers and evaluated according to the formula: tumor volume (mm^3^) = 4/3 × π × length/2 × (width/2)^2^. When the volume reached ~100 mm^3^, the mice were randomized into four groups (*n* = 5): group 1 treated with 100 μl of vehicle; group 2 treated with 3 mg/kg TAX; group 3 treated with 3 mg/kg TAX + 20 mg/kg HA/VER@ZIF‐8; and group 4 treated with 3 mg/kg TAX + 20 mg/kg HA/VER@ZIF‐8 + 25 mg/kg ezetimibe. This was administered by i.p. injections once every 2 days. Mice were euthanized when obvious differences between each group were obtained. Subsequently, tumor tissues were removed and fixed immediately for further analysis. For the tumor recurrence model, Du145^TXR^ cells were subcutaneously injected into the mice as described above. Xenografts were removed surgically when their average volume reached ~250 mm^3^. Isolated tumor tissues were fixed immediately in 4% paraformaldehyde. Mice were randomized into two groups: group 1 treated with 3 mg/kg TAX + 20 mg/kg HA/VER@ZIF‐8; group 2 treated with 3 mg/kg TAX + 20 mg/kg HA/VER@ZIF‐8 + 25 mg/kg ezetimibe. A course of three injections was given every 2 days and recoveries followed postoperatively for 1 week. Subsequently, after all tumors of group 1 became palpable, tumor tissues of the two studied groups were observed and isolated immediately.

### Statistical analysis

GraphPad 7 software (GraphPad, La Jolla, CA, USA) was used for all statistical analysis and graph plotting. Statistical differences were evaluated using one‐way ANOVA or Student’s *t*‐test. Each experiment was performed at least three times and error bars indicate SD unless otherwise indicated.

## Author contributions

**Zhe Zhang:** Conceptualization; Data curation; Visualization; Writing – original draft; Writing – review and editing. **Siyuan Qin:** Data curation; Methodology. **Yan Chen:** Data curation; Methodology. **Li Zhou:** Data curation. **Mei Yang:** Data curation. **Yongquan Tang:** Data curation. **Jing Zuo:** Data curation. **Jian Zhang:** Data curation. **Atsushi Mizokami:** Data curation. **Edouard C Nice:** Supervision; Writing – review and editing. **Hai‐Ning Chen:** Conceptualization; Supervision; Funding acquisition; Methodology; Writing – review and editing. **Canhua Huang:** Conceptualization; Supervision; Funding acquisition; Validation; Project administration. **Xiawei Wei:** Conceptualization; Supervision; Funding acquisition; Validation; Project administration; Writing – review and editing.

In addition to the CRediT author contributions listed above, the contributions in detail are:

XW, CH, and H‐NC designed the conception. ZZ, SQ, and YC designed and performed the experiments. ZZ drafted the manuscript. LZ, MY, YT, and JZu acquired and analyzed the data. JZh and AM established Du145^TXR^ cancer cell model. ECN revised the manuscript and polished the language. All authors read and approved the final manuscript.

## Disclosure statement and competing interests

The authors declare that they have no conflict of interest.

## Supporting information



Appendix

Expanded View Figures PDF

Movie EV1

Source Data for Expanded View and Appendix

Source Data for Figure 1

Source Data for Figure 2

Source Data for Figure 4

Source Data for Figure 5

Source Data for Figure 6

## Data Availability

Data (RNA sequencing of MCs and MPCs) generated data in this study has been deposited in the SRA database (https://www.ncbi.nlm.nih.gov/sra) with the accession numbers PRJNA777049 and in the GEO database (https://www.ncbi.nlm.nih.gov/geo) with the accession number GSE187441.
