# Supplementary material for: Inhibition of NPC1L1 disrupts adaptive responses of drug‐tolerant persister cells to chemotherapy
Source: EMBO Mol Med. 2022 Jan 13;14(2):e14903. doi: 10.15252/emmm.202114903 (PMC8819355; doi:10.15252/emmm.202114903)
Supplement: Supplementary file 1 — Appendix [file EMMM-14-e14903-s003.pdf]

Appendix for:

Inhibition of NPC1L1 disrupts adaptive responses of drug-tolerant persister cells to chemotherapy

The appendix include:

Appendix Figure S1-S5 and figure legends

Appendix Figure S1

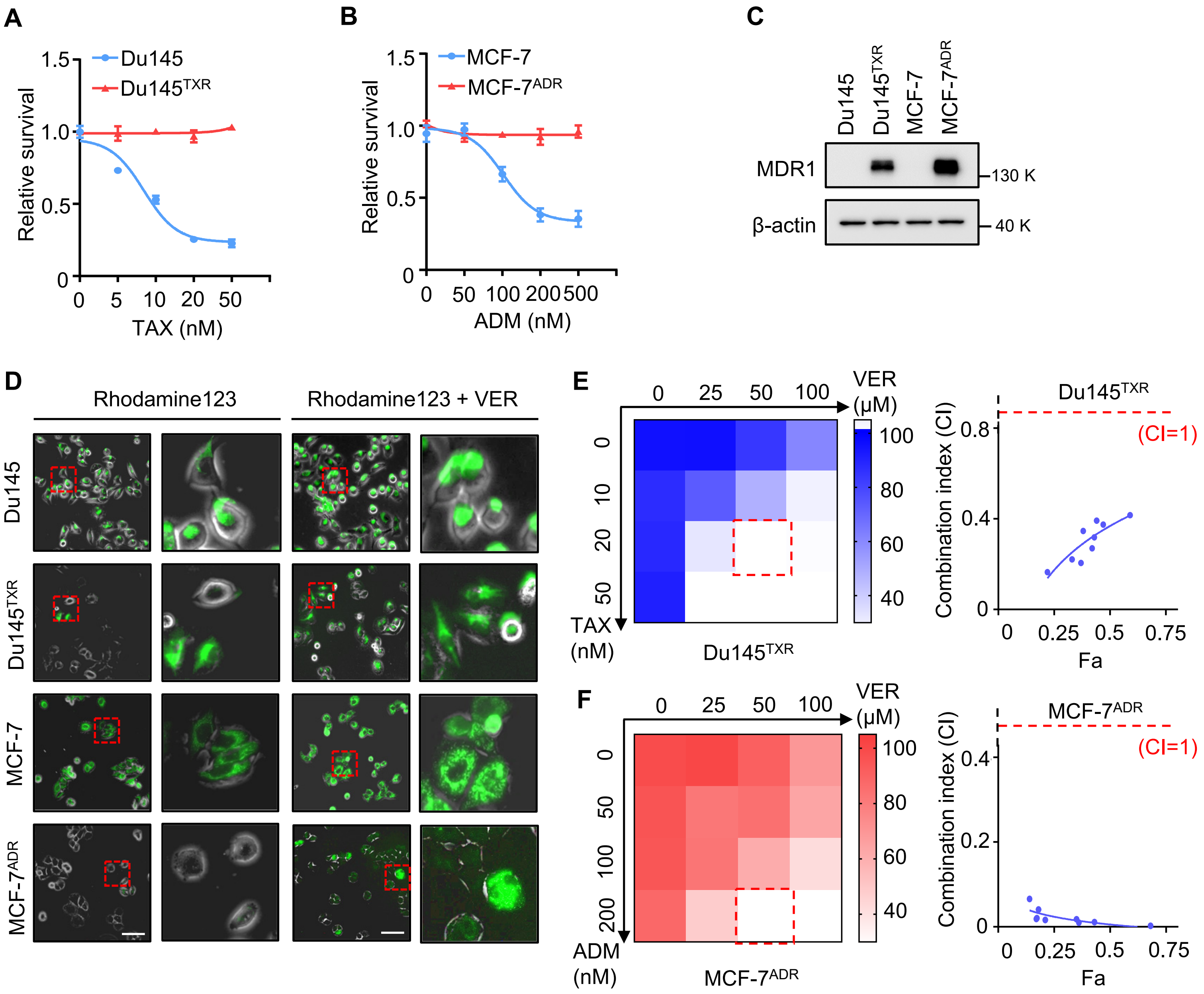

Appendix Figure S1. MDR1 identified by RNA-seq plays a key role in driving multi-drug resistance.

A-B, cell viability of (A) Du145/Du145^{TXR} or (B) MCF-7/MCF-7^{ADR} cells treated with the indicated concentrations of taxol (TAX) or adriamycin (ADM) for 72 hours. Mean with ± SD.

C, immunoblotting of MDR1 in Du145/Du145^{TXR} and MCF-7/MCF-7^{ADR} cells.

D, efflux function analysis of MDR1 by rhodamine123 staining of Du145/Du145^{TXR} or MCF-7/MCF-7^{ADR} cells. Scale bar, 50 μm.

E-F, cell viability of (F) Du145^{TXR} or (G) MCF-7^{ADR} cells treated with the indicated doses of TAX or ADM in combination with verapamil (VER) for 72 hours (left), combination index (CI) of the different combinations of TAX or ADM and VER were calculated to measure the level of synergism (CI < 1) or antagonism (CI > 1) using the Chou-Talalay method (right). Given the essential role of MDR1 in maintaining the resistant phenotype of Du145^{TXR} and MCF-7^{ADR} cell lines, the concentration of verapamil was chosen at a maximum but non-lethal dose to minimize the contributions made by MDR1 to drug efflux. As for the chemotherapeutic agents (taxol or adriamycin), we selected the concentrations which displayed significantly inhibitory effects (>70 percent inhibition) on tumor cells as cut-off values for further exploration. The red dotted line indicates adopted combination (50 μM verapamil/20 nM taxol or 200 nM adriamycin) in this study unless otherwise specified.

Data information: Results are representative of three independent experiments.

Appendix Figure S2

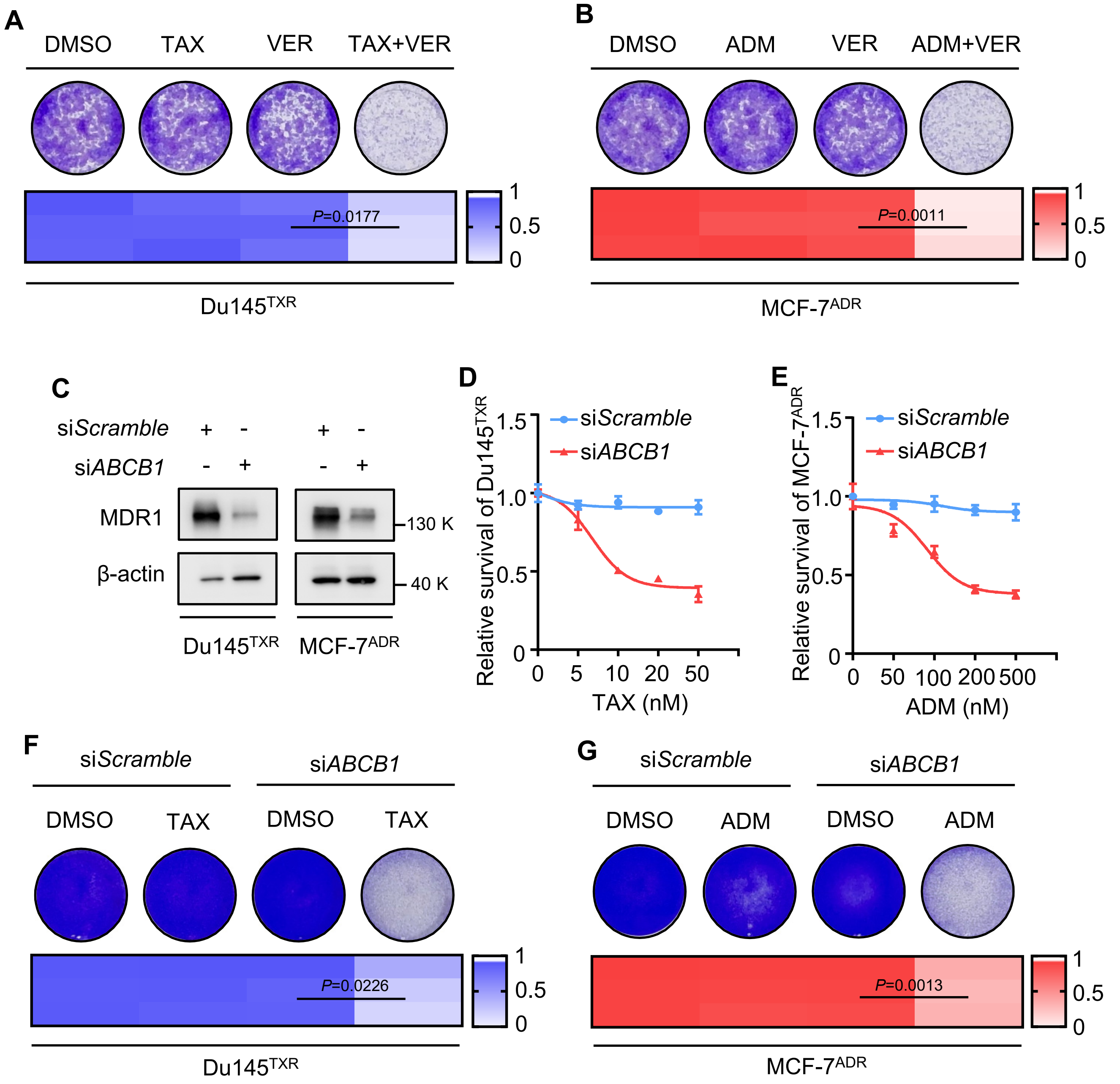

Appendix Figure S2. Targeting MDR1 partially enables drug-resistant cancer cells to recover chemosensitivity.

A-B, colony formation assay and quantification of (A) Du145^{TXR} or (B) MCF-7^{ADR} cells treated with indicated agents. One-way ANOVA was used to analyze statistical differences.

C, immunoblotting of MDR1 in Du145^{TXR} or MCF-7^{ADR} cells transfected with si*ABCB1* or si*Scramble*.

D-E, cell viability of (D) Du145^{TXR} or (E) MCF-7^{ADR} cells transfected with si*ABCB1* or si*Scramble* followed by treatment with the indicated concentrations of TAX or ADM for 72 hours. Mean with ± SD.

F-G, colony formation assay and quantification of (F) Du145^{TXR} or (G) MCF-7^{ADR} cells transfected with si*ABCB1* or si*Scramble* followed by treatment with treated with indicated agents. One-way ANOVA was used to analyze statistical differences.

Data information: Results are representative of three independent experiments.

Appendix Figure S3

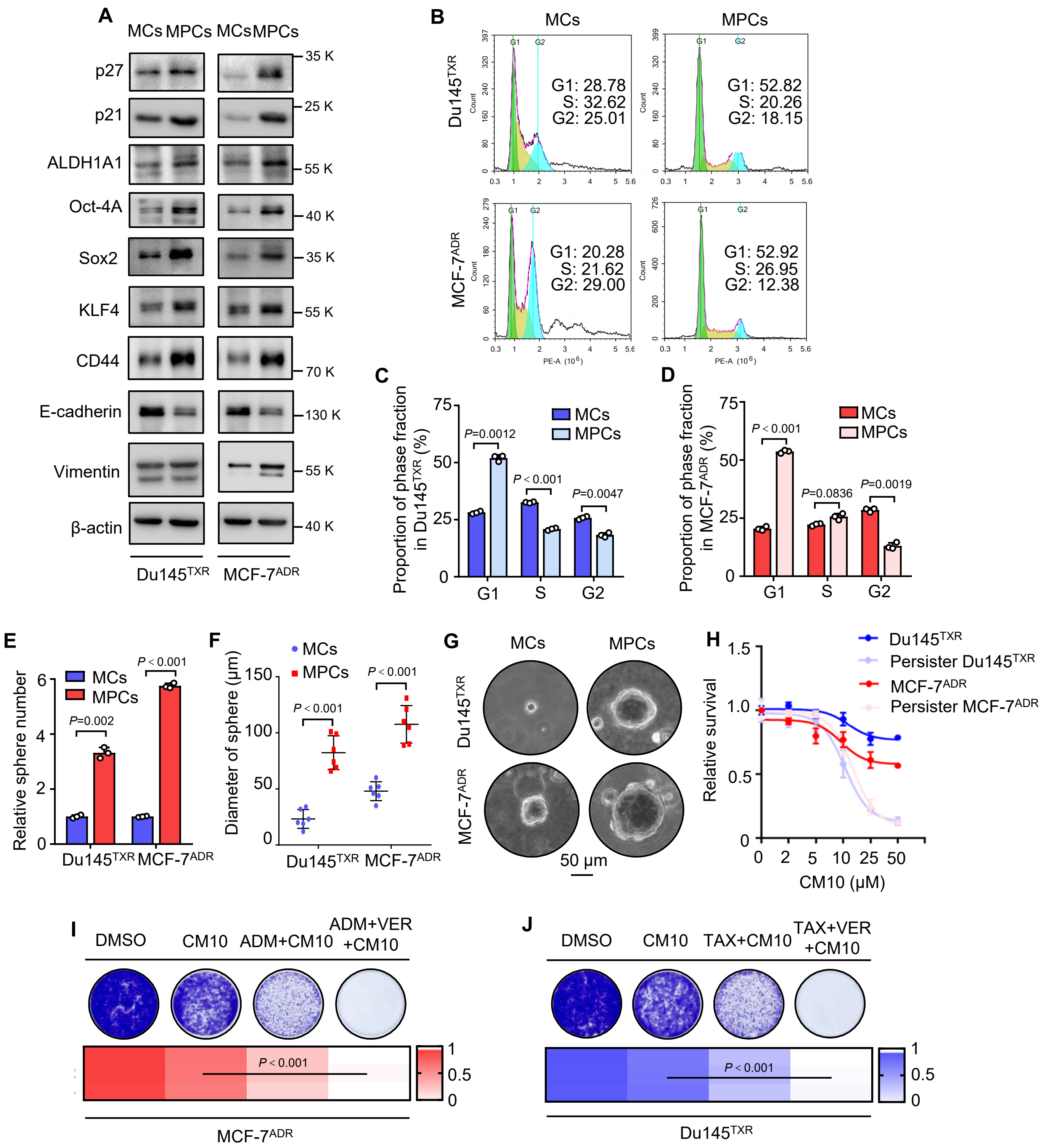

Appendix Figure S3. The characteristics of MPCs include induction of cell cycle arrest, increased stemness, and activation of EMT.

A, immunoblotting of p27, p21, ALDH1A1, Oct-4A, Sox2, KLF4, CD44, E-cadherin and Vimentin in MCs and MPCs of Du145^{TXR} or MCF-7^{ADR} cells.

B-D, flow cytometric analysis of cell cycle in MCs and MPCs of Du145^{TXR} or MCF-7^{ADR} cells. (B) Representative images and quantification of ROS in (C) Du145^{TXR} or (D) MCF-7^{ADR} cells are shown. Student's t test was used to analyze statistical differences. Mean with ± SD.

E-G, sphere formation analysis including number (E), diameter(F) and representative images (G) of spheres in MCs and MPCs of Du145^{TXR} or MCF-7^{ADR} cells. Scale bar, 50 µm. Student's t test was used to analyze statistical differences. Mean with ± SD.

H, cell viability of Du145^{TXR}/Persister Du145^{TXR} and MCF-7^{ADR}/Persister MCF-7^{ADR} cells treated with the indicated concentrations of CM10 for 24 hours. Mean with ± SD.

I-J, colony formation assay and quantification of (I) Du145^{TXR} or (J) MCF-7^{ADR} cells treated with indicated agents. One-way ANOVA was used to analyze statistical differences.

Data information: Results are representative of three independent experiments.

Appendix Figure S4

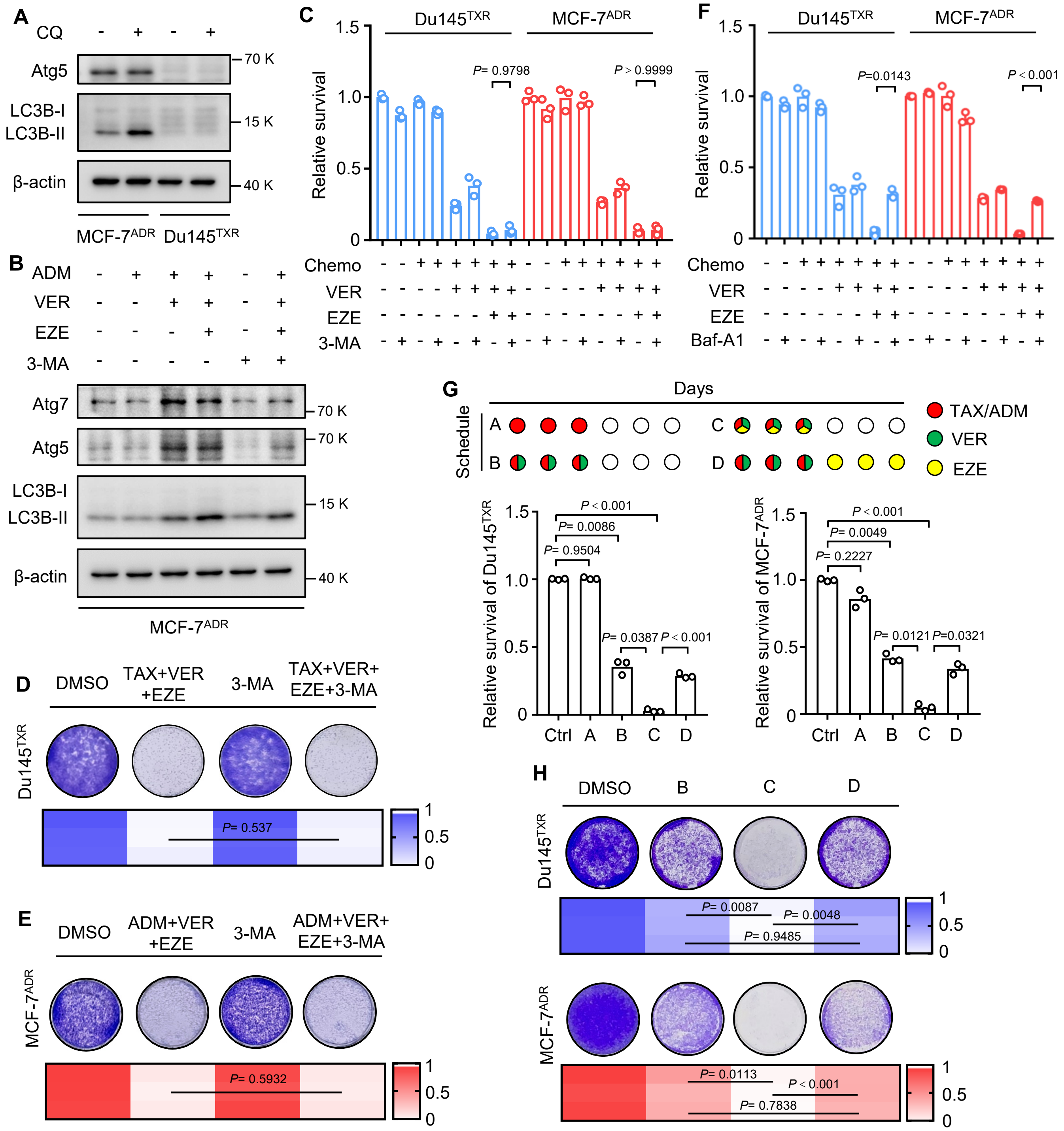

Appendix Figure S4. Triple -combination treatment-induced cell death is involved in macropinocytosis but not autophagy.

A, immunoblotting of LC3B, Atg5, in Du145^{TXR} and MCF-7^{ADR} cells treated with or without 10 μM CQ for 24 hours.

B, immunoblotting of LC3B, Atg5, and Atg7 in MCF-7^{ADR} cells treated with indicated agents for 24 hours.

C, cell viability of Du145^{TXR} and MCF-7^{ADR} cells treated with the indicated agents for 72 hours (Chemo represents 20 nM taxol or 200 nM adriamycin for Du145^{TXR} or MCF-7^{ADR}, respectively; 50 μM verapamil; 25 μM ezetimibe; 1 mM 3-MA). One-way ANOVA was used to analyze statistical differences. Mean with ± SD.

D-E, colony formation assay and quantification of (D) Du145^{TXR} or (E) MCF-7^{ADR} cells treated with the indicated agents. One-way ANOVA was used to analyze statistical differences.

F, cell viability of Du145^{TXR} and MCF-7^{ADR} cells treated with indicated agents for 72 hours (Chemo represents 20 nM taxol or 200 nM adriamycin for Du145^{TXR} or MCF-7^{ADR}, respectively; 50 μM verapamil; 25 μM ezetimibe; 100 nM Baf-A1). One-way ANOVA was used to analyze statistical differences. Mean with ± SD.

G, cell viability of Du145^{TXR} or MCF-7^{ADR} cells treated with indicated schedules. One-way ANOVA was used to analyze statistical differences. Mean with ± SD.

H, colony formation assay and quantification of Du145^{TXR} or MCF-7^{ADR} cells treated with indicated schedules. One-way ANOVA was used to analyze statistical differences.

Data information: Results are representative of three independent experiments.

Appendix Figure S5

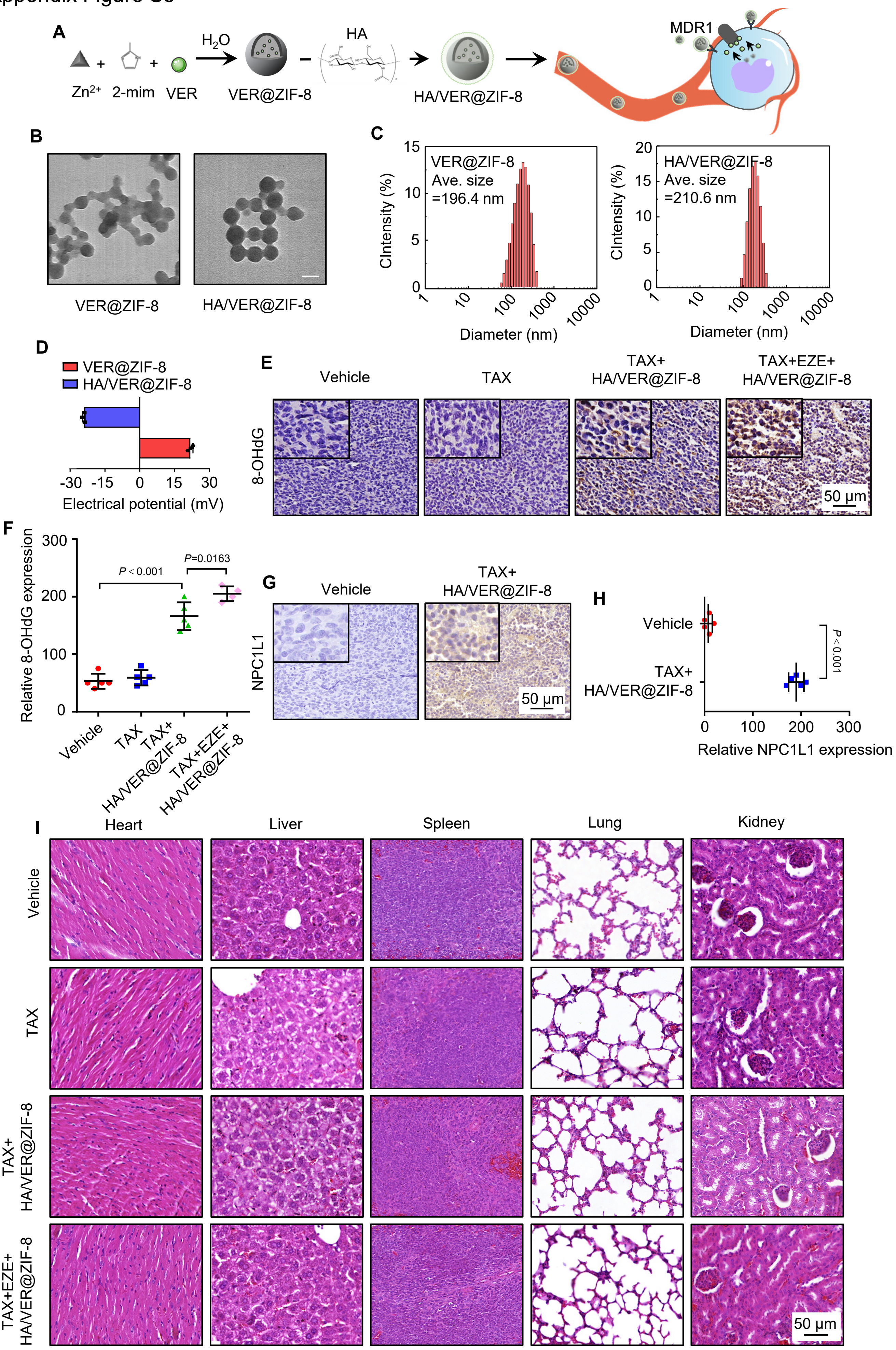

Appendix Figure S5. Nanocarrier-based therapy triggers oxidative stress and has no obvious toxicity *in vivo*.

A, schematic illustration of HA-decorated ZIF-8 as a VER delivery vehicle.

B-C, (B) TEM images and (C) size distributions of VER@ZIF-8 and HA/VER@ZIF-8.

D, electrical potential analysis of VER@ZIF-8 and HA/VER@ZIF-8. Mean with ± SD.

E-F, immunohistochemical staining of 8-OHdG in indicated groups. (E) Representative images and (F) relative immunohistochemical scores are shown. Scale bar, 50 μm. One-way ANOVA was used to analyze statistical differences. Mean with ± SD.

G-H, immunohistochemical staining of NPC1L1 in indicated groups. (G) Representative images and (H) relative immunohistochemical scores are shown. Student's t test was used to analyze statistical differences. Mean with ± SD.

I, H&E staining of heart, liver, spleen, lung and kidney of male nude mice in indicated groups. Scale bar, 50 μm.
