# Supplementary material for: Inhibition of NPC1L1 disrupts adaptive responses of drug‐tolerant persister cells to chemotherapy
Source: EMBO Mol Med. 2022 Jan 13;14(2):e14903. doi: 10.15252/emmm.202114903 (PMC8819355; doi:10.15252/emmm.202114903)
Supplement: Supplementary file 2 — Expanded View Figures PDF [file EMMM-14-e14903-s007.pdf]

Expanded View Figures

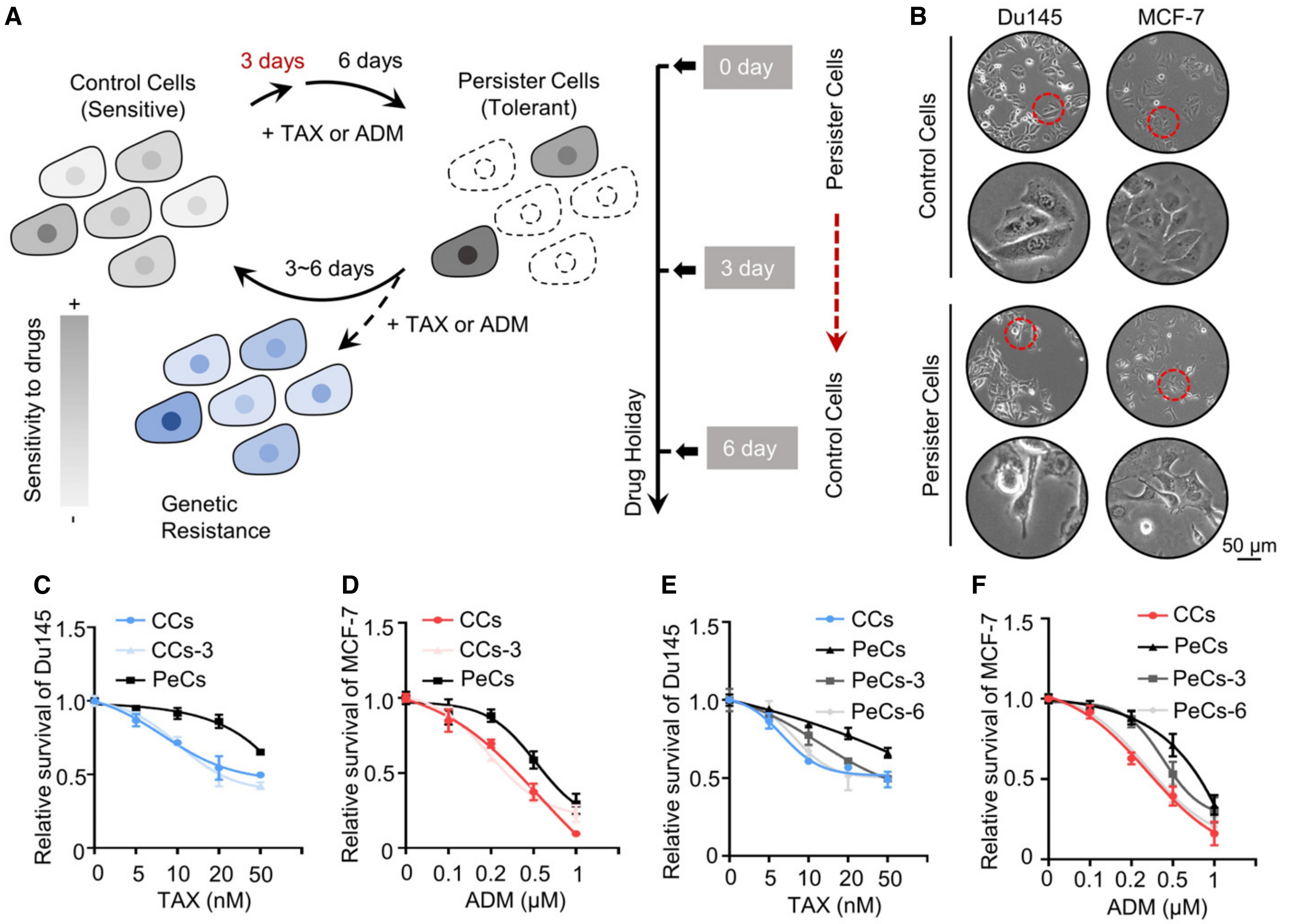

Figure EV1. Control cancer cells transform into DTP states in response to chemotherapy.

A Schematic of drug persister cancer cell generation and subsequent analyses.

B Phase contrast images of the morphological change in Du145 or MCF-7 cells followed by chemotherapy treatment for 72 h. Red circles mark magnified areas (bottom). Scale bar, 50 μm (low-magnification images).

C, D Cell viability of control cancer cells (CCs), 3 days treated control cancer cells (CCs-3), and persister cancer cells (PeCs) of (C) Du145 or (D) MCF-7 cells treated with the indicated concentrations of taxol (TAX) or adriamycin (ADM) for 24 h. Mean with ± SD.

E, F Cell viability of CCs, PeCs, 3 days drug withdrawn PeCs (PeCs-3), and 6 days drug withdrawn PeCs (PeCs-6) of (E) Du145 or (F) MCF-7 cells treated with the indicated concentrations of taxol (TAX) or adriamycin (ADM) for 24 h. Mean with ± SD.

Data information: Results are representative of three independent experiments.
Source data are available online for this figure.

Figure EV2. Signaling pathways identified in MPCs by performing RNA-seq.

A KEGG/PID pathway analysis showed the changed signaling pathways of MCs vs. MPCs in Du145^{TXR} and MCF-7^{ADR}.
B, C GSEA indicated GO Myc targets of MCs vs. MPCs in (B) Du145^{TXR} and (C) MCF-7^{ADR}.
D, E GSEA indicated GO cell cycle and DNA replication of MCs vs. MPCs in (D) Du145^{TXR} and (E) MCF-7^{ADR}.

▶

 © 2022 The Authors

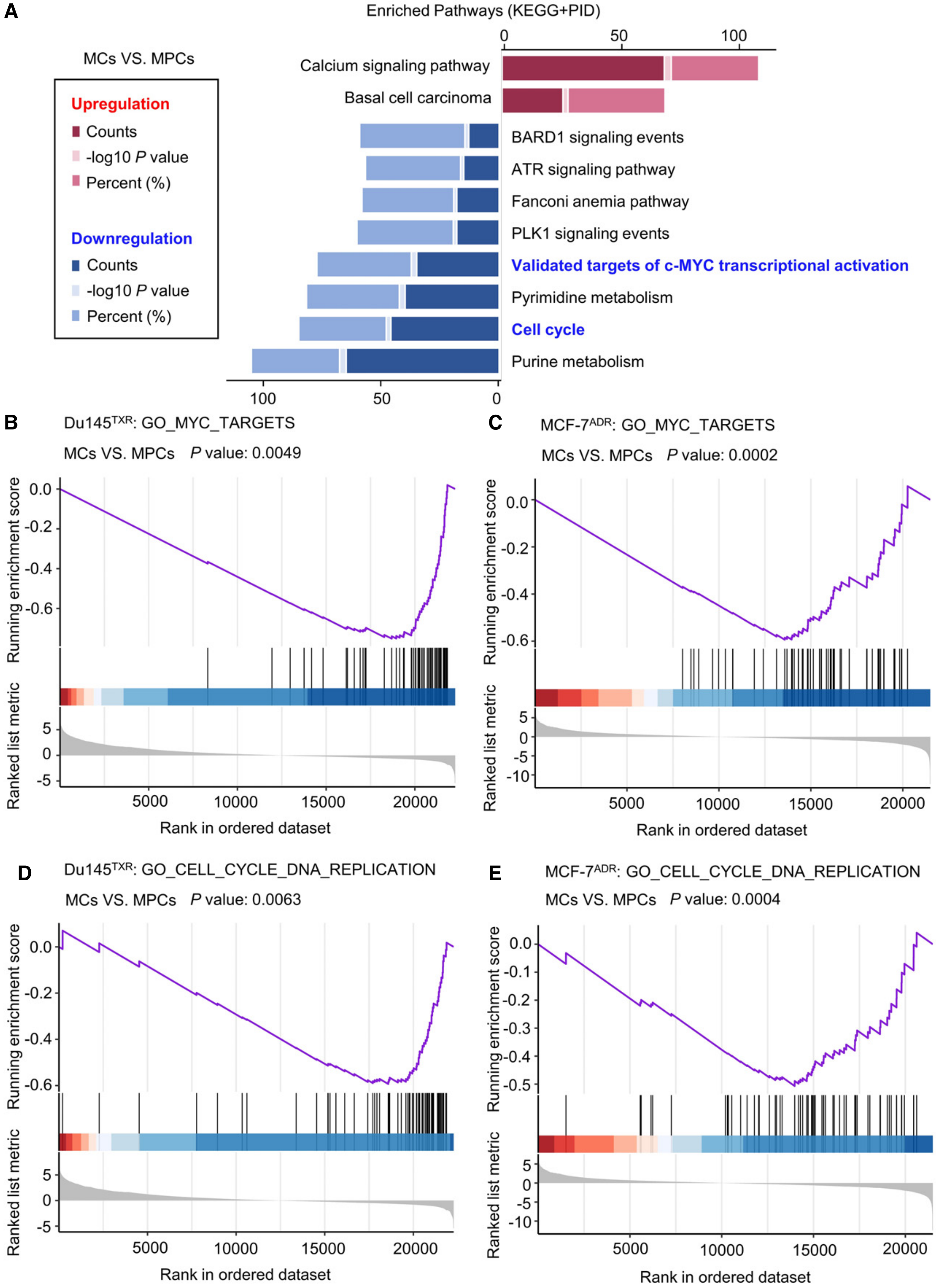
A
Enriched Pathways (KEGG+PID)
MCs VS. MPCs
Upregulation
Counts
-log10 P value
Percent (%)
Downregulation
Counts
-log10 P value
Percent (%)
Calcium signaling pathway
Basal cell carcinoma
BARD1 signaling events
ATR signaling pathway
Fanconi anemia pathway
PLK1 signaling events
Validated targets of c-MYC transcriptional activation
Pyrimidine metabolism
Cell cycle
Purine metabolism
B
Du145TXR: GO_MYC_TARGETS
MCs VS. MPCs P value: 0.0049
C
MCF-7ADR: GO_MYC_TARGETS
MCs VS. MPCs P value: 0.0002
D
Du145TXR: GO_CELL_CYCLE_DNA_REPLICATION
MCs VS. MPCs P value: 0.0063
E
MCF-7ADR: GO_CELL_CYCLE_DNA_REPLICATION
MCs VS. MPCs P value: 0.0004
Running enrichment score
Ranked list metric
Rank in ordered dataset

Figure EV2.

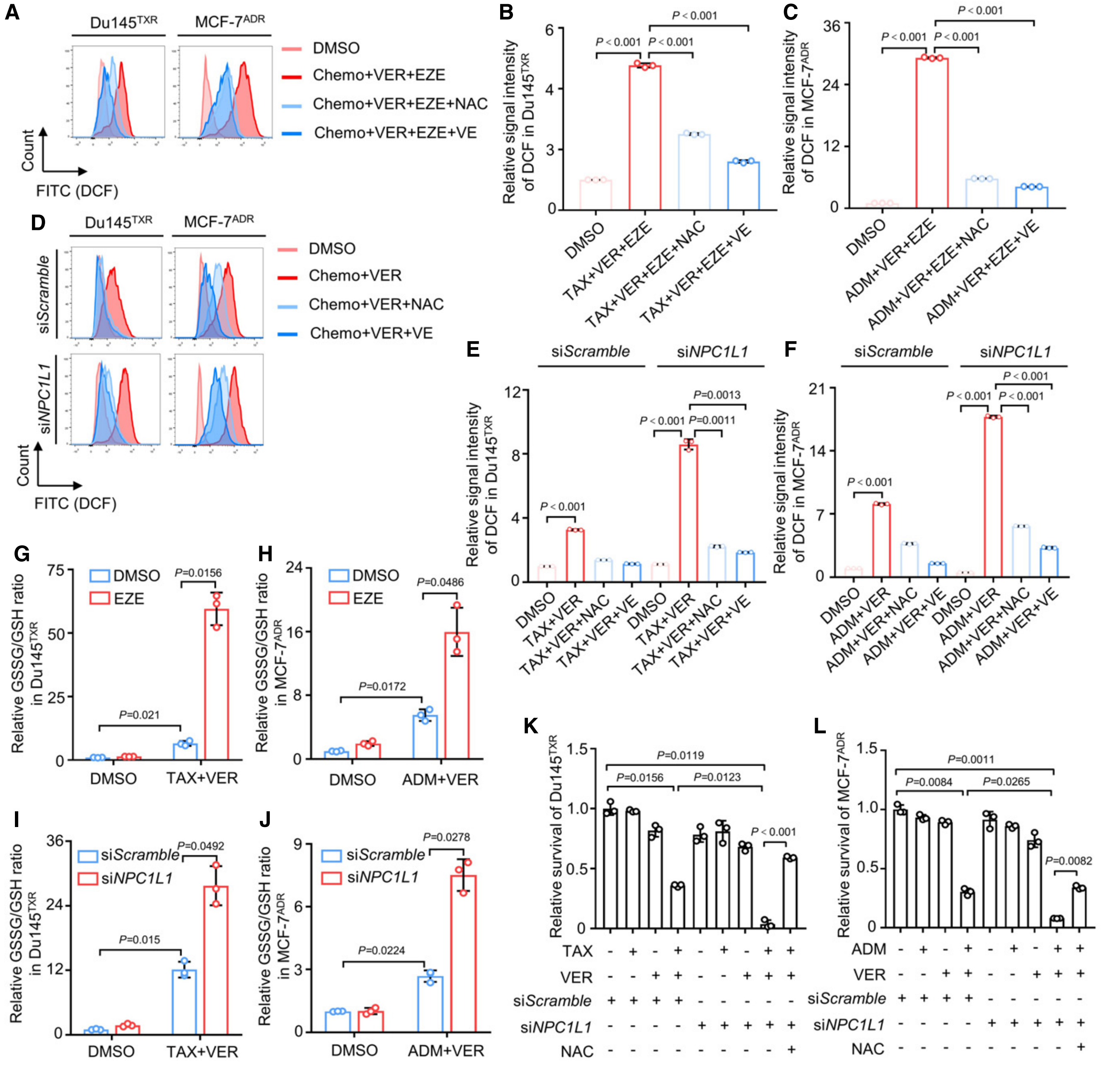

Figure EV3. Inhibition of NPC1L1 induces oxidative stress-mediated cell death.

A–C Flow cytometric analysis of ROS levels in Du145^{TXR} or MCF-7^{ADR} cells treated with indicated agents for 24 h. Chemo represents 20 nM taxol or 200 nM adriamycin for Du145^{TXR} or MCF-7^{ADR}, respectively. (A) Representative images and quantification of ROS in (B) Du145^{TXR} or (C) MCF-7^{ADR} cells are shown. One-way ANOVA was used to analyze statistical differences. Mean with ± SD.

D–F Flow cytometric analysis of ROS levels in Du145^{TXR} or MCF-7^{ADR} cells transfected with si*NPC1L1* or si*Scramble* followed by indicated treatment for 24 h. Chemo represents 20 nM taxol or 200 nM adriamycin for Du145^{TXR} or MCF-7^{ADR}, respectively. (D) Representative images and quantification of ROS in (E) Du145^{TXR} or (F) MCF-7^{ADR} cells are shown. One-way ANOVA was used to analyze statistical differences. Mean with ± SD.

G, H GSSG/GSH ratio measurement of (G) Du145^{TXR} or (H) MCF-7^{ADR} cells treated with indicated agents for 24 h. One-way ANOVA was used to analyze statistical differences. Mean with ± SD.

I, J GSSG/GSH ratio measurement of (I) Du145^{TXR} or (J) MCF-7^{ADR} transfected with si*NPC1L1* or si*Scramble* followed by indicated treatment for 24 hours. One-way ANOVA was used to analyze statistical differences. Mean with ± SD.

K, L Cell viability of (K) Du145^{TXR} or (L) MCF-7^{ADR} cells transfected with si*NPC1L1* or si*Scramble* followed by treatment with the indicated agents for 72 h. One-way ANOVA was used to analyze statistical differences. Mean with ± SD.

Data information: Results are representative of three independent experiments.

 © 2022 The Authors

Figure EV4. Lipotoxicity from NPC1L1 inhibition-induced oxidative stress may partially contribute to cell death of MPCs.

A Immunofluorescence analysis of NPC1L1 in $Du145^{TXR}$ and MCF-7^{ADR} treated with indicated agents for 12 h. Scale bar, 10 μm.

B, C Cellular total cholesterol analysis in MCs and MPCs of (B) $Du145^{TXR}$ or (C) MCF-7^{ADR} cells treated with 25 μM ezetimibe for 12 h. One-way ANOVA was used to analyze statistical differences. Mean with ± SD.

D–F Flow cytometric analysis of lipid ROS using BODIPY 581/591 C11 reagent in $Du145^{TXR}$ or MCF-7^{ADR} cells treated with indicated agents for 24 h. Chemo represents 20 nM taxol or 200 nM adriamycin for $Du145^{TXR}$ or MCF-7^{ADR}, respectively. (D) Representative images and quantification of lipid ROS in (E) $Du145^{TXR}$ or (F) MCF-7^{ADR} cells are shown. One-way ANOVA was used to analyze statistical differences. Mean with ± SD.

G–I Flow cytometric analysis of lipid ROS using BODIPY 581/591 C11 reagent in $Du145^{TXR}$ or MCF-7^{ADR} cells transfected with si*NPC1L1* or si*Scramble* followed by indicated treatment for 24 h. Chemo represents 20 nM taxol or 200 nM adriamycin for $Du145^{TXR}$ or MCF-7^{ADR}, respectively. (G) Representative images and quantification of lipid ROS in (H) $Du145^{TXR}$ or (I) MCF-7^{ADR} cells are shown. One-way ANOVA was used to analyze statistical differences. Mean with ± SD.

J–K MDA levels measurement of (J) $Du145^{TXR}$ or (K) MCF-7^{ADR} cells treated with indicated agents for 24 h. One-way ANOVA was used to analyze statistical differences. Mean with ± SD.

L, M MDA levels in (L) $Du145^{TXR}$ or (M) MCF-7^{ADR} cells transfected with si*NPC1L1* or si*Scramble* followed by indicated treatment for 24 h. One-way ANOVA was used to analyze statistical differences. Mean with ± SD.

N, O Cell viability of (N) $Du145^{TXR}$ or (O) MCF-7^{ADR} cells transfected with si*NPC1L1* or si*Scramble* followed by treatment with the indicated agents cultured in normal or serum-free medium for 72 h. One-way ANOVA was used to analyze statistical differences. Mean with ± SD.

P, Q Colony formation assay and quantification of (P) $Du145^{TXR}$ or (Q) MCF-7^{ADR} cells transfected with si*NPC1L1* or si*Scramble* followed by treatment with indicated agents. One-way ANOVA was used to analyze statistical differences.

R, S Flow cytometric analysis of ROS levels in MCs and MPCs of $Du145^{TXR}$ and MCF-7^{ADR} cells. (R) Representative images and (S) quantification of ROS are shown. Student's *t*-test was used to analyze statistical differences. Mean with ± SD.

Data information: Results are representative of three independent experiments.
Source data are available online for this figure.

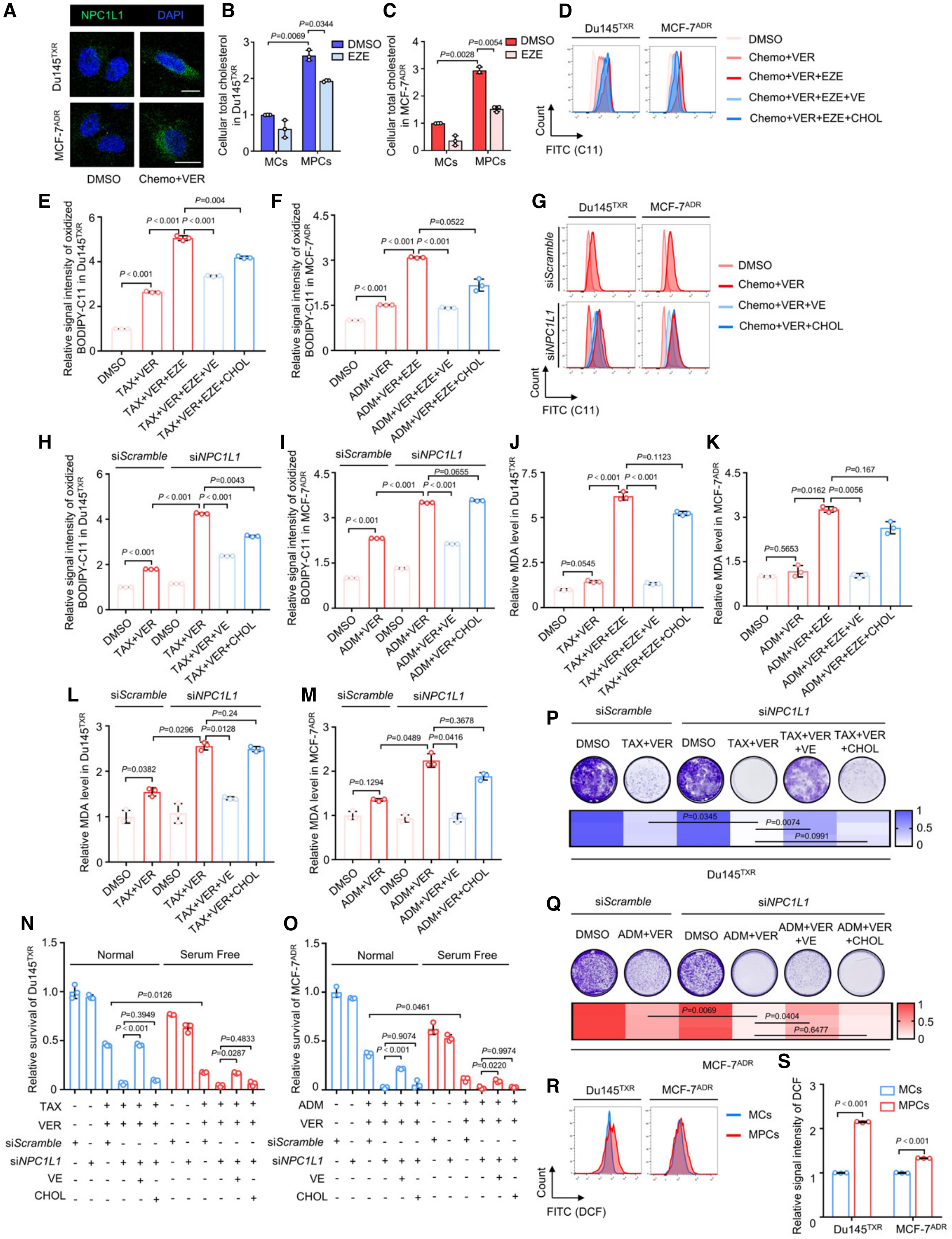

Figure EV4.

 © 2022 The Authors

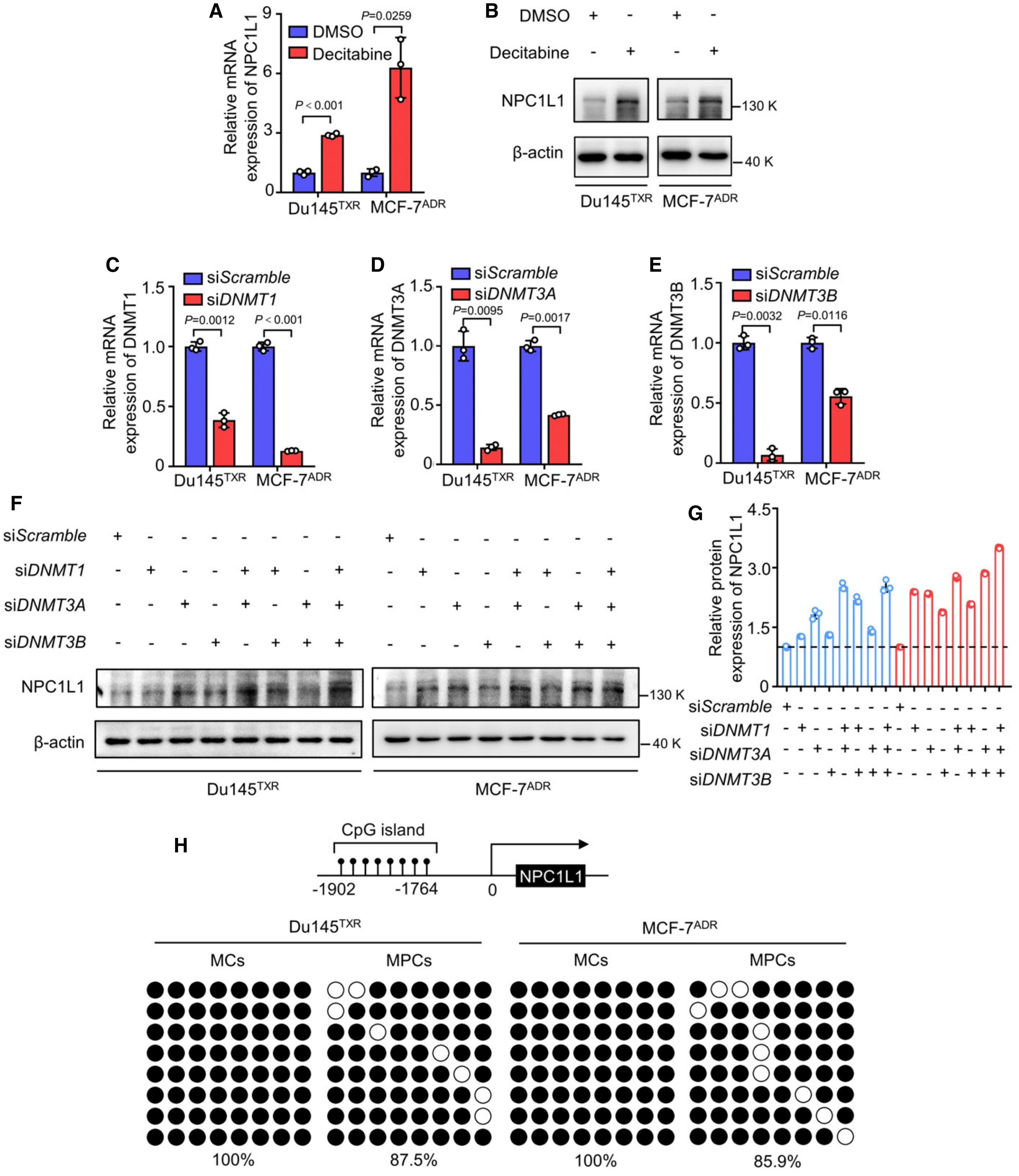
A
DMSO
Decitabine
Relative mRNA expression of NPC1L1
P < 0.001
P=0.0259
Du145TXR
MCF-7ADR
B
DMSO
Decitabine
NPC1L1
β-actin
130 K
40 K
Du145TXR
MCF-7ADR
C
siScramble
siDNMT1
Relative mRNA expression of DNMT1
P=0.0012
P < 0.001
D
siScramble
siDNMT3A
Relative mRNA expression of DNMT3A
P=0.0095
P=0.0017
E
siScramble
siDNMT3B
Relative mRNA expression of DNMT3B
P=0.0032
P=0.0116
F
siScramble
siDNMT1
siDNMT3A
siDNMT3B
NPC1L1
β-actin
130 K
40 K
Du145TXR
MCF-7ADR
G
Relative protein expression of NPC1L1
H
CpG island
-1902
-1764
0
NPC1L1
Du145TXR
MCF-7ADR
MCs
MPCs
100%
87.5%
100%
85.9%

Figure EV5.

◀ **Figure EV5. Decreased DNMTs-mediated DNA methylation contributes to upregulation of NPC1L1 in MPCs.**

A qRT-PCR analysis of NPC1L1 in Du145^{TXR} or MCF-7^{ADR} cells treated with 2 μM decitabine for 72 h. Student's *t*-test was used to analyze statistical differences. Mean with ± SD.

B Immunoblotting of NPC1L1 in Du145^{TXR} or MCF-7^{ADR} cells treated with 2 μM decitabine for 72 h.

C–E qRT-PCR analyses of (C) DNMT1, (D) DNMT3A, and (E) DNMT3B in Du145^{TXR} or MCF-7^{ADR} cells transfected with si*DNMT1*/si*DNMT3A*/si*DNMT3B* or siScramble. Student's *t*-test was used to analyze statistical differences. Mean with ± SD.

F-G (F) Immunoblotting of NPC1L1 in Du145^{TXR} or MCF-7^{ADR} cells transfected with si*DNMT1*/si*DNMT3A*/si*DNMT3B* or si*Scramble*. (G) The expression levels of NPC1L1 in (F) were quantified. Dotted lines represent value 1 (relative protein expression of NPC1L1). Mean with ± SD.

H Bisulfite genomic sequencing of the methylation level of eight CpGs within CpG island of NPC1L1 promotor in MCs and MPCs of Du145^{TXR} or MCF-7^{ADR} cells. The black circles and empty circles indicate methylated and unmethylated CpG dinucleotides, respectively.

Data information: Results are representative of three independent experiments.
Source data are available online for this figure.

© 2022 The Authors
