# Supplementary material for: Inhibition of NPC1L1 disrupts adaptive responses of drug‐tolerant persister cells to chemotherapy
Source: EMBO Mol Med. 2022 Jan 13;14(2):e14903. doi: 10.15252/emmm.202114903 (PMC8819355; doi:10.15252/emmm.202114903)
Supplement: Supplementary file 3 — Movie EV1 [file EMMM-14-e14903-s001.zip › Movie_EV1.docx]

**Movie EV1. Utilization of NPC1L1 inhibitor ezetimibe induces macropinocytosis in MPCs.**

Legend of video: Taxol (TAX) plus verapamil (VER) triggers cell death by induction of apoptosis in Du145^TXR^ cells. Specifically, the apoptotic Du145^TXR^ cells are disengaging from the surrounding matrix, while the cell membrane shrinks inward, the cytoplasm concentrates and the nuclear ruptures. Co-treatment of NPC1L1 inhibitor ezetimibe (EZE) with TAX/VER induces macropinocytosis in Du145^TXR^ cells, evidenced by hard and frequent contractions as well as occurrence of cytoplasmic vacuolization. Scale bar, 1 μm; 1 second representing 1 hour.
