# Supplementary figures and images for: Inhibition of NPC1L1 disrupts adaptive responses of drug‐tolerant persister cells to chemotherapy
Source: EMBO Mol Med. 2022 Jan 13;14(2):e14903. doi: 10.15252/emmm.202114903 (PMC8819355; doi:10.15252/emmm.202114903)

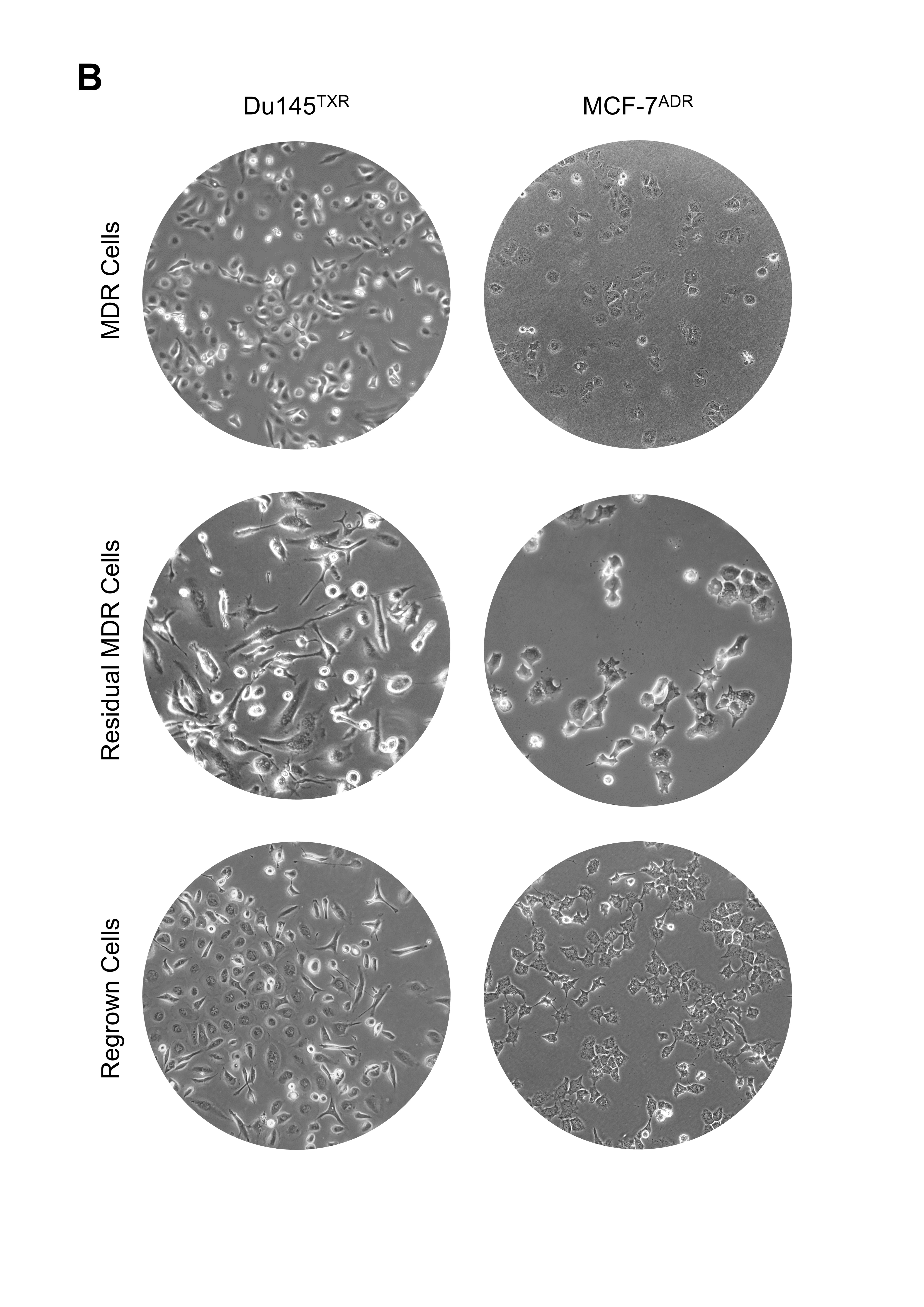

Supplement: Supplementary file 5 — Source Data for Figure 1 [file EMMM-14-e14903-s008.jpg]

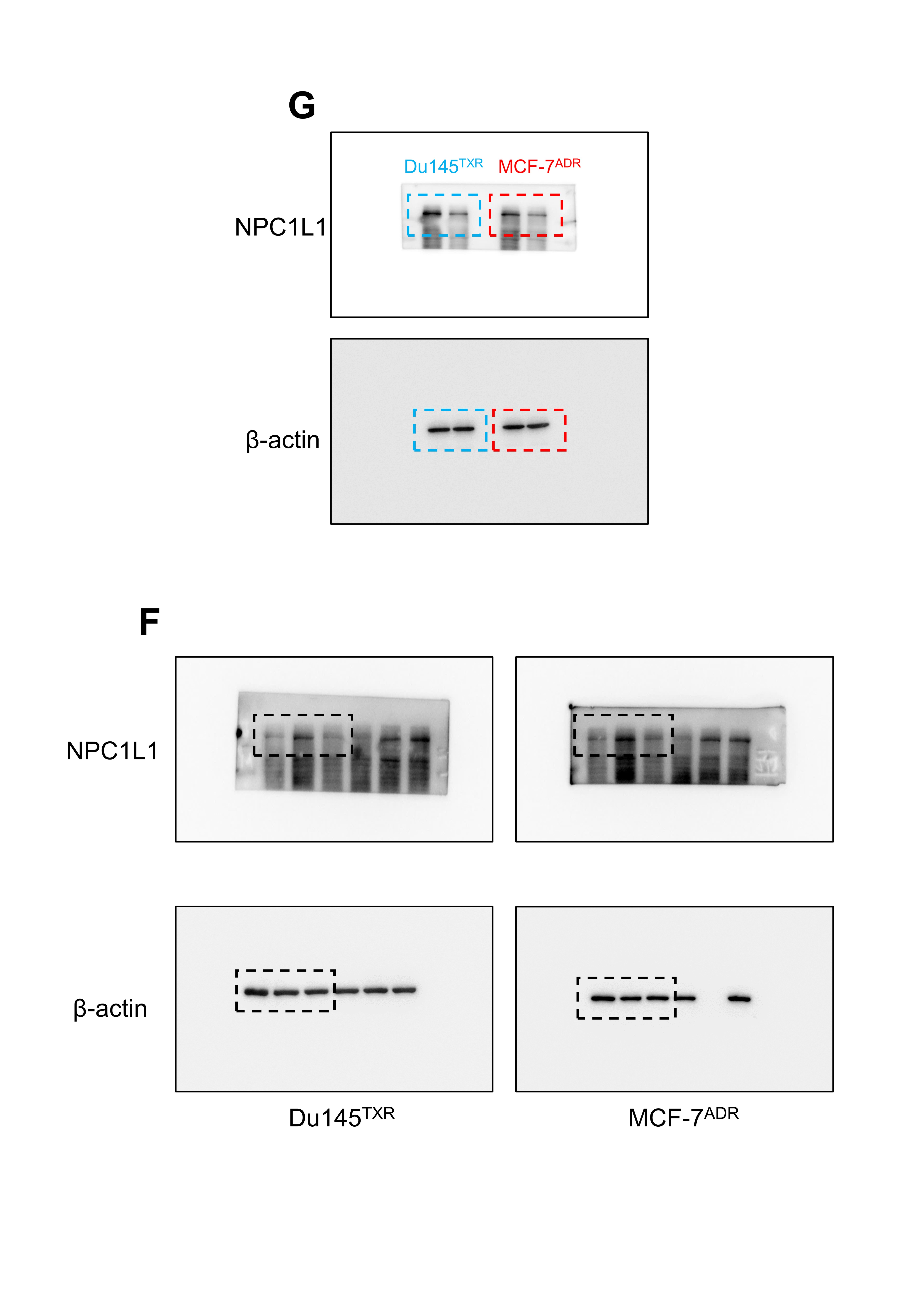

Supplement: Supplementary file 6 — Source Data for Figure 2 [file EMMM-14-e14903-s004.jpg]

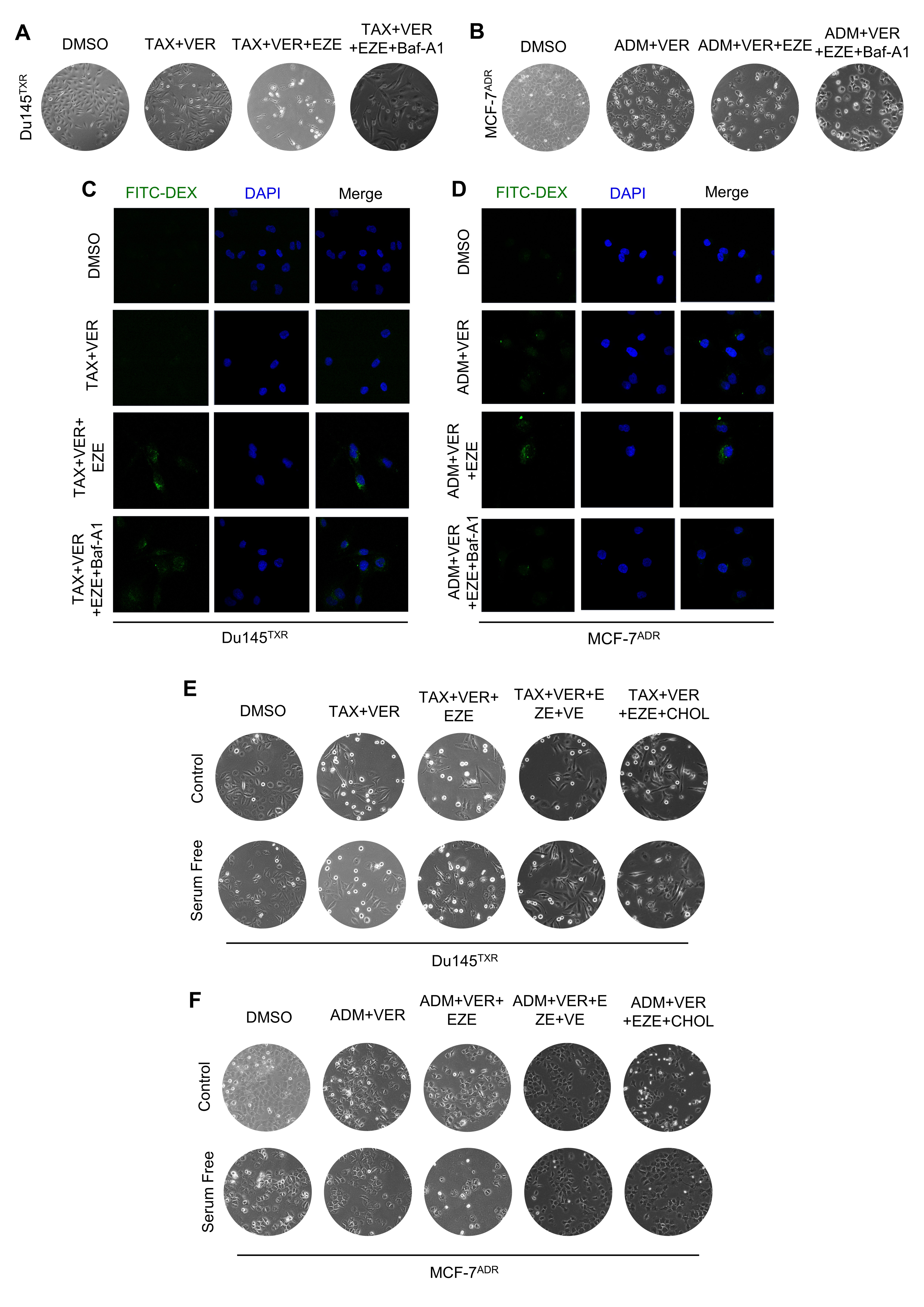

Supplement: Supplementary file 7 — Source Data for Figure 4 [file EMMM-14-e14903-s010.jpg]

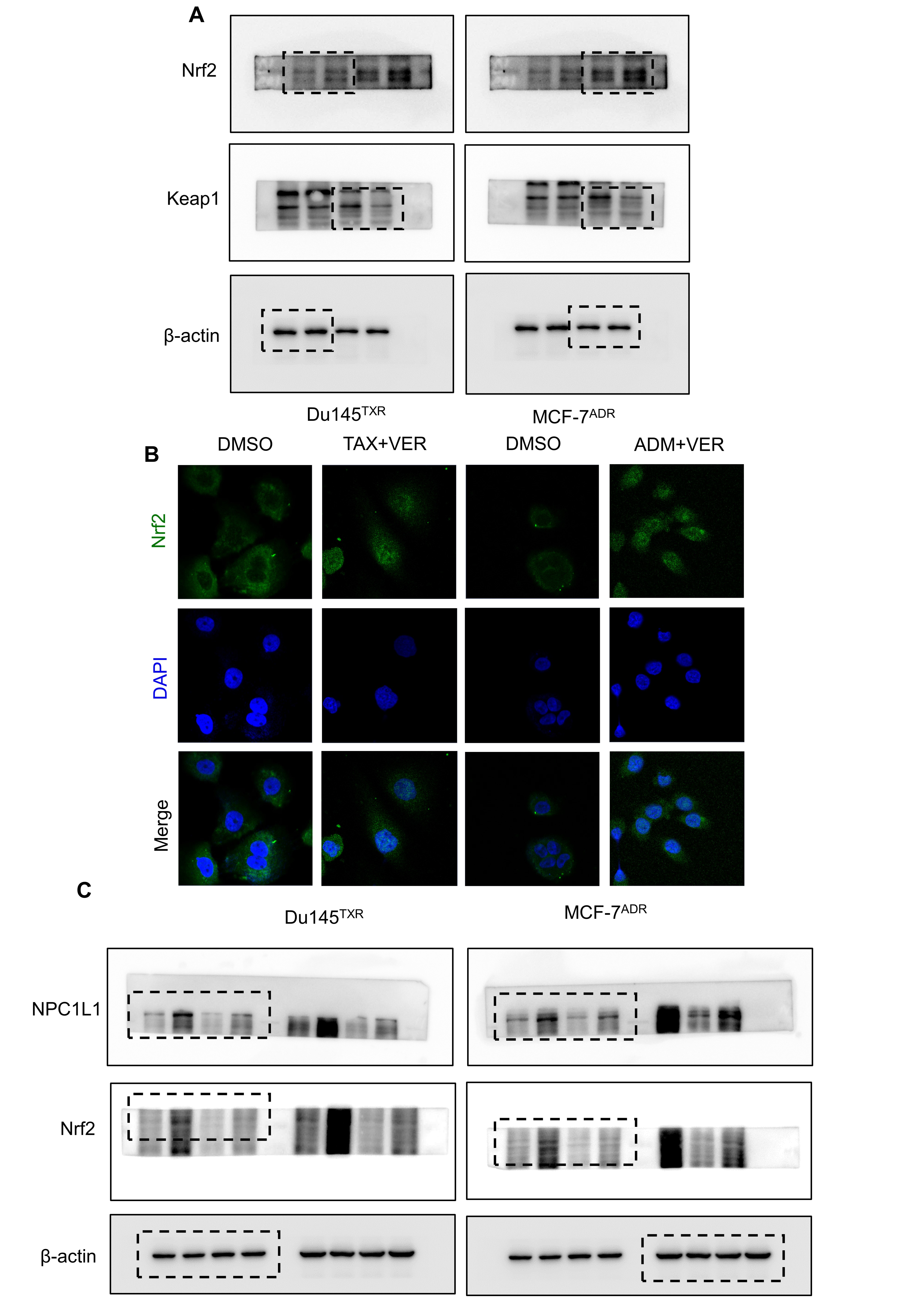

Supplement: Supplementary file 8 — Source Data for Figure 5 [file EMMM-14-e14903-s006.jpg]

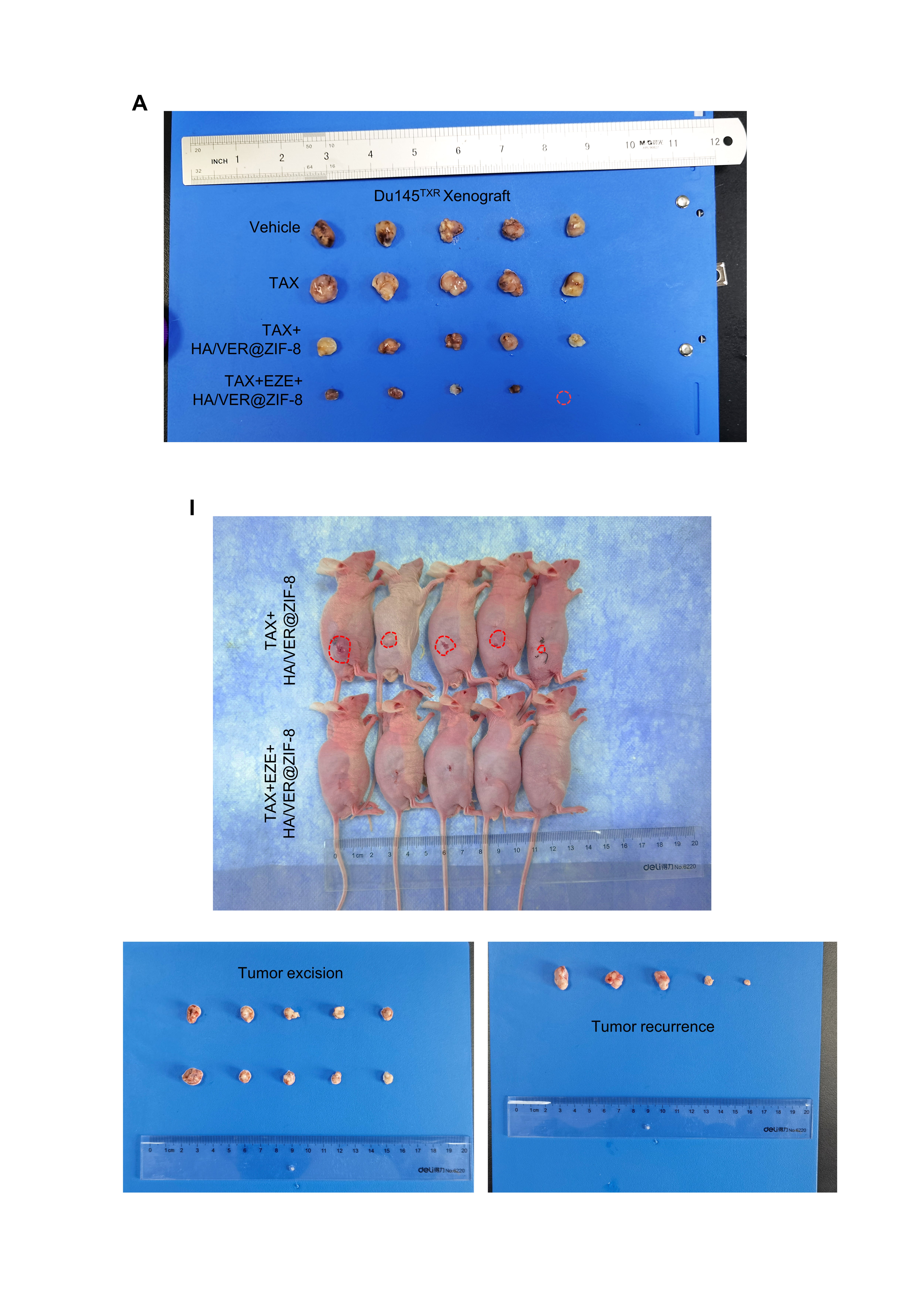

Supplement: Supplementary file 9 — Source Data for Figure 6 [file EMMM-14-e14903-s005.jpg]
