# Supplementary material for: Inhibition of NPC1L1 disrupts adaptive responses of drug‐tolerant persister cells to chemotherapy
Source: EMBO Mol Med. 2022 Jan 13;14(2):e14903. doi: 10.15252/emmm.202114903 (PMC8819355; doi:10.15252/emmm.202114903)
Supplement: Supplementary file 10 [file EMMM-14-e14903-s009.pdf]

EMBO Molecular Medicine

Inhibition of NPC1L1 disrupts adaptive responses of drug-tolerant persister cells to chemotherapy

Zhe Zhang, Siyuan Qin, Yan Chen, Li Zhou, Mei Yang, Yongquan Tang, Jing Zuo, Jian Zhang, Mizokami Atsushi, Edouard Nice, Hai-Ning Chen, Canhua Huang, and Xiawei Wei
DOI: 10.15252/emmm.202114903

Corresponding authors: Xiawei Wei (xiaweiwei@scu.edu.cn) , Hai-Ning Chen (hnchen@scu.edu.cn), Canhua Huang (hcanhua@scu.edu.cn)

Review Timeline:

Submission Date:	27th Jul 21
Editorial Decision:	11th Aug 21
Revision Received:	8th Nov 21
Editorial Decision:	7th Dec 21
Revision Received:	10th Dec 21
Accepted:	14th Dec 21

Editor: Lise Roth

Transaction Report:

(Note: With the exception of the correction of typographical or spelling errors that could be a source of ambiguity, letters and reports are not edited. Depending on transfer agreements, referee reports obtained elsewhere may or may not be included in this compilation. Referee reports are anonymous unless the Referee chooses to sign their reports.)

11th Aug 2021

Dear Prof. Wei,

Thank you for submitting your work to EMBO Molecular Medicine. We have now heard back from the referees who agreed to evaluate your manuscript. As you will see below, while reviewer #1 is overall supportive of publication of your study, reviewers #2 and #3 raise substantial concerns on your work, which unfortunately preclude its publication in EMBO Molecular Medicine in its current form.

The reviewers acknowledge the interest of the study, but they also raise a number of issues, pertaining mostly to the selection and generation of DTP cell lines, to the lack of mechanistic understanding, and to general flaws in the rationale and experimental design.

If you feel you can satisfactorily address the points listed by the referees, you may wish to submit a revised version of your manuscript. Please attach a covering letter giving details of the way in which you have handled each of the referees' concerns. A revised manuscript will once again be subject to review, and we cannot guarantee at this stage that the eventual outcome will be favorable.

Revised manuscripts should be submitted within three months of a request for revision; they will otherwise be treated as new submissions, except under exceptional circumstances in which a short extension is obtained from the editor.

Should you find that the requested revisions are not feasible within the constraints outlined here and prefer, therefore, to submit your paper elsewhere, we would welcome a message to this effect.

When submitting your revised manuscript, please carefully review the instructions that follow below. We perform an initial quality control of all revised manuscripts before re-review; failure to include requested items will delay the evaluation of your revision.

We require:

1) A .docx formatted version of the manuscript text (including legends for main figures, EV figures and tables). Please make sure that the changes are highlighted to be clearly visible.

2) Individual production quality figure files as .eps, .tif, .jpg (one file per figure). For guidance, download the 'Figure Guide PDF' (https://www.embopress.org/page/journal/17574684/authorguide#figureformat).

3) A .docx formatted letter INCLUDING the reviewers' reports and your detailed point-by-point responses to their comments. As part of the EMBO Press transparent editorial process, the point-by-point response is part of the Review Process File (RPF), which will be published alongside your paper.

4) A complete author checklist, which you can download from our author guidelines (https://www.embopress.org/page/journal/17574684/authorguide#submissionofrevisions). Please insert information in the checklist that is also reflected in the manuscript. The completed author checklist will also be part of the RPF.

5) It is mandatory to include a 'Data Availability' section after the Materials and Methods. Before submitting your revision, primary datasets produced in this study need to be deposited in an appropriate public database, and the accession numbers and database listed under 'Data Availability'. Please remember to provide a reviewer password if the datasets are not yet public (see https://www.embopress.org/page/journal/17574684/authorguide#dataavailability).

In case you have no data that requires deposition in a public database, please state so in this section. Note that the Data Availability Section is restricted to new primary data that are part of this study.

6) For data quantification: please specify the name of the statistical test used to generate error bars and P values, the number (n) of independent experiments (specify technical or biological replicates) underlying each data point and the test used to calculate p-values in each figure legend. The figure legends should contain a basic description of n, P and the test applied. Graphs must include a description of the bars and the error bars (s.d., s.e.m.).

7) We would also encourage you to include the source data for figure panels that show essential data. Numerical data should be provided as individual .xls or .csv files (including a tab describing the data). For blots or microscopy, uncropped images should be submitted (using a zip archive if multiple images need to be supplied for one panel). Additional information on source data and instruction on how to label the files are available at
.

8) Our journal encourages inclusion of *data citations in the reference list* to directly cite datasets that were re-used and obtained from public databases. Data citations in the article text are distinct from normal bibliographical citations and should directly link to the database records from which the data can be accessed. In the main text, data citations are formatted as follows: "Data ref: Smith et al, 2001" or "Data ref: NCBI Sequence Read Archive PRJNA342805, 2017". In the Reference list, data citations must be labeled with "[DATASET]". A data reference must provide the database name, accession number/identifiers and a resolvable link to the landing page from which the data can be accessed at the end of the reference. Further instructions are available at .

9) We replaced Supplementary Information with Expanded View (EV) Figures and Tables that are collapsible/expandable online. A maximum of 5 EV Figures can be typeset. EV Figures should be cited as 'Figure EV1, Figure EV2" etc... in the text and their respective legends should be included in the main text after the legends of regular figures.

- For the figures that you do NOT wish to display as Expanded View figures, they should be bundled together with their legends in a single PDF file called *Appendix*, which should start with a short Table of Content. Appendix figures should be referred to in the main text as: "Appendix Figure S1, Appendix Figure S2" etc.

- Additional Tables/Datasets should be labeled and referred to as Table EV1, Dataset EV1, etc. Legends have to be provided in a separate tab in case of .xls files. Alternatively, the legend can be supplied as a separate text file (README) and zipped together with the Table/Dataset file.

See detailed instructions here:
.

10) The paper explained: EMBO Molecular Medicine articles are accompanied by a summary of the articles to emphasize the major findings in the paper and their medical implications for the non-specialist reader. Please provide a draft summary of your article highlighting
- the medical issue you are addressing,
- the results obtained and
- their clinical impact.

This may be edited to ensure that readers understand the significance and context of the research. Please refer to any of our published articles for an example.

11) For more information: There is space at the end of each article to list relevant web links for further consultation by our readers. Could you identify some relevant ones and provide such information as well? Some examples are patient associations, relevant databases, OMIM/proteins/genes links, author's websites, etc...

12) Every published paper now includes a 'Synopsis' to further enhance discoverability. Synopses are displayed on the journal webpage and are freely accessible to all readers. They include a short stand first (maximum of 300 characters, including space) as well as 2-5 one-sentences bullet points that summarizes the paper. Please write the bullet points to summarize the key NEW findings. They should be designed to be complementary to the abstract - i.e. not repeat the same text. We encourage inclusion of key acronyms and quantitative information (maximum of 30 words / bullet point). Please use the passive voice. Please attach these in a separate file or send them by email, we will incorporate them accordingly.

Please also suggest a striking image or visual abstract to illustrate your article as a PNG file 550 px wide x 300-600 px high.

13) As part of the EMBO Publications transparent editorial process initiative (see our Editorial at http://embomolmed.embopress.org/content/2/9/329), EMBO Molecular Medicine will publish online a Review Process File (RPF) to accompany accepted manuscripts.
In the event of acceptance, this file will be published in conjunction with your paper and will include the anonymous referee reports, your point-by-point response and all pertinent correspondence relating to the manuscript. Let us know whether you agree with the publication of the RPF and as here, if you want to remove or not any figures from it prior to publication.
Please note that the Authors checklist will be published at the end of the RPF.

EMBO Molecular Medicine has a "scooping protection" policy, whereby similar findings that are published by others during review or revision are not a criterion for rejection. Should you decide to submit a revised version, I do ask that you get in touch after three months if you have not completed it, to update us on the status.

I look forward to receiving your revised manuscript.

Yours sincerely,

Lise Roth

Lise Roth, PhD
Editor
EMBO Molecular Medicine

Use this link to login to the manuscript system and submit your revision: Link Not Available

***** Reviewer's comments *****

Referee #1 (Comments on Novelty/Model System for Author):

Drug Tolerant Persister cells (DTPs) are of major interest in relation to drug resistance. The current manuscript identifies a frequently used drug for treatment of high cholesterol as an effective drug for killing such DTPs that arise after chemotherapy. This is very exciting and novel and has direct utility in the clinic.

Referee #1 (Remarks for Author):

The authors have done a remarkable job in identifying the vulnerabilities of DPTs that arise after treatment of chemotherapy resistant cells that express MDR1 with chemo+MDR1 inhibitors. The data shows that such MDR-DTPs have upreagulated NPC1L1, which is activated by an NRF2-mediated the anti-oxidant response in these cells. Ezetimibe is an established inhibitor of NPC1L1 and use of this drug in combination with chemo and verapamil leads to macropinocytosis-mediated cell death. The experiments are well-controlled and in general compelling. The clincial relevance of their finding is immediate, given that ezetimibe is prescribed widely for hypercholesterol therapy without major side effects.
Strengths of the manuscript are that the experiments are carried out in two indpendent MDR resistant cell line models, the deep analyses of the mechanism of DTP survival and the compelling in vivo validation of the drug combinations.
I am unable to judge the specifics of the delivery of verapamil in vivo, I trust other reviewers may have an opinion on the suitability of this vehicle.
I am highly supportive of immediate publicaiton of this work, given its potential direct clinical application.

Referee #2 (Comments on Novelty/Model System for Author):

The selection and generation of DTP cell lines caused confusion. It seems that in the combination index experiments a drug concentration was chosen that inhibited growth around 30% (?), if the legend in S1G and S1H, blue to white and red to white, respectively, indicate growth inhibition. If that is the case, then a large surviving fraction has been chosen for further study that could contain resistant cells.

Referee #2 (Remarks for Author):

In the study "Inhibition of NPC1L1 disrupts adaptive responses of drug-tolerant
persister cells to chemotherapy-induced oxidative stress", Zhang et all describe the isolation and characterization of drug tolerant peristent cells generated from Adriamycin resistant MCF-7 and taxol-resistant Du145 cells. After MDR1 overexpression was established to convey drug-resistance, cells sensitivity was re-established after co-treating cells with the MDR-inhibitor verapamil. Treatment persistent cells were isolated and characterized morphologically and functionally as DTP cells. Gene expression (RNA-seq)analysis compared then MDR cancer cells with residual, regrown MDR cancer cells, and identified NPC1L1 as differential expressed gene and it's upregulation supported cell survival in response to cytotoxic in MDR-persister cells. The authors further showed that NPC1L1 compromises oxidative stress by promoting vitamin E uptake during chemotherapeutic agents/verapamil treatment in MDR cancer cells. Inhibition of NPC1L1 using ezetimibe suggested cell death by micropinocytosis. NRF2 was shown to be one of the transcription factors targeting the transcription of NPC1L1. Lastly, in a xenograft mouse models, the authors show that taxol/verapamil/ezetimibe delivered via a nanoparticle approach can decrease tumor burden in the xenograft model. Currently third generation p-gp inhibitors are in development. If NPC1L1 turns out to be a desired drug target in cancer to target DTP, then this nanotechnology may be of high importance. However, as PK and PD studies are missing (and beyond the scope), this is difficult to assess.

This study offers plentiful of experiments and detail in some instances.
However, overall, the manuscript is very difficult to follow in the beginning where DTPs are isolated or generated. Typos and

grammatical errors contribute to this. There is also sometimes a lack of proper rationale and data interpretation.

Importantly, the selection and generation of DTP cell lines caused great confusion. As these cells build the foundation of the study, this is an important point. Cells used here were isolated from drug-resistant cancer cell lines. Is the assumption that drug resistant cell lines overexpressing MDR have subpopulations of DTPs? If so, that needs to be stated and convincingly demonstrated. For example, to identify DTP cells, combination index experiments were used. There, a drug concentration that distinguished DTPs from the 'other cells' was chosen that inhibited growth around 30% (?), if the legend in S1G and S1H, blue to white and red to white, respectively, indicate growth inhibition. If that is the case, then this large surviving fraction could easily contain resistant cells. The rationale for this cut off is missing.
Would it not be better to find a dose that leads to unaffected growth of resistant cells and isolate cells that grow only modestly?

In addition, although text states from line 164 to 167: cells that survived cytotoxic exposure were collected; it is not described in detail how that was done.

What was the rationale for treatment times chosen in 1C, 1D?
1F and 1G are not well described: "drug tolerant rate of (F) Du145/Du145TXR
(20 nM taxol / 20 nM taxol + 50 μM verapamil) or (G) MCF-7/MCF-7ADR (200 nM
adriamycin / 200 nM adriamycin + 50 μM verapamil) cells treated with drug on the
indicated days. Drug tolerant rate defined by compared cell viability followed treatment,
RMCs vs. MCs or PeCs vs.PaCs, > 1 representing drug tolerant". Which ratio is referred to? MCF-7/MCF-7ADR or RMCs vs. MCs? If RMCs grow faster that MC, couldn't it be that a resistant clone is responsible for this phenotype?

1D is mislabeled in the legend: MCF-7ADR

Knocking down MDR should increase sensitivity, this is not novel.

Line 171: "MC that have never been treated" - which drug is referred to? If it is Adriamycin or taxol, is that statement then true? How were the resistant cell lines initially made?

Line 182: Fig. S3A: shouldn't that read Fig. S2A? Pls check entire paragraph for figure mislabelling.

Line 185-191: this statement is confusing as Figs 1F and 1G don't show that.

Regarding the exclusion of autophagy: line 300: not likely caused by autophagy; That needs to be visited in more detail, especially since the autophagy inhibitor bafilomycin a1 was used and a phenotype was found in response to it. Other autophagy markers need to be examined.

Lastly, stemness and EMT are also markers of drug resistant cells, supporting the point made earlier regarding the specificity of the cells identified as DTPs.

Referee #3 (Comments on Novelty/Model System for Author):

Experimental design has significant flaws as outlined in my review below.

Referee #3 (Remarks for Author):

In this study, Zhang et al. investigate mechanisms underlying the multidrug resistant (MDR) phenotype in adriamycin-resistant MCF7 cells and taxol-resistant DU145 cells. They propose a drug tolerant persister (DTP) state underlies resistance to chemotherapeutic drugs and the MDR1 inhibitor verapamil. They further propose this resistance is characterized by resistance to oxidative stress through NRF2-mediated elevation in the cholesterol and alpha-tocopherol transporter, NPC1L1. The authors then use the NPC1L1 inhibitor ezetimibe to show that co-treatment with this agent plus chemotherapy plus verapamil produces ROS production and macropinocytosis-induced cell death in vitro and prevents tumor recurrence in vivo. These studies are interesting and innovative. The occurrence of MDR and DTP in the cell lines used are well-characterized and the siNPC1L1 survival data are convincing. The major weakness is that their main premise of redox homeostasis dysregulation in the MDR process is not convincingly established, and there are some significant conceptual inaccuracies underlying their experimental design and analyses. Furthermore, while the authors have shown a strong link of NPC1L1 to the DTP state, the mechanism by which NPC1L1 inhibition eradicates these cells is also not convincing - it seems ferroptosis would be a more likely mechanism than macropinocytosis given the role of NPC1L1. Overall, some depth and clarification should be provided in these areas to strengthen and streamline the study.

Specific comments are below.

1. Vitamin E has been shown to prevent lipid peroxidation by scavenging lipid peroxyl radicals. It has not been shown to

scavenge any other type of radical. DCFDA measures peroxides which do not in fact directly produce lipid peroxides. These are produced by a reaction of the hydroxyl radical (not scavenged by Vit E) with fatty acids, a process facilitated by labile iron. Have the authors assessed whether the siNPC1L1 or Eze induced effects can be rescued by desferal and ferrostatin-1? It's entirely possible that the main effect of siNPC1L1 is ferroptosis given its role as a cholesterol and VitE transporter. The authors should also consider measuring malondialdehyde as a specific product of lipid peroxidation, and whether this is increased by siNPC1L1 or Eze and/or whether VitE or cholesterol addition alter its levels. Regardless it is highly unlikely that Vit E is providing redox protective effects to MPCs by reducing the ROS species being measured by the authors.

2. Secondly, the data provided regarding ROS increase in Fig 3 are not rigorous. ROS-reactive fluorescence quantification via microscopy is notoriously inaccurate/non-homogenous, and quantitative methods such as flow cytometry should be employed.

3. Furthermore, the role of ROS needs to be carried through to the other experiments to show convincing involvement of redox dysregulation i.e. do NAC or VE actually reduce ROS levels in addition to promoting survival? If NPCL1L1 is indeed the mediator of increased NRF2 in MPCs then siNPC1L1 should also correlate with redox stress and elevated ROS. These have not been provided. Without such data, the fact that Eze could be having off-target effects on ROS levels, as many drugs do, cannot be ruled out. Ideally, to invoke oxidative stress via NPC1L1 inhibition, effects should be shown at the level of specific oxidative stress markers such as 8-oxodG, protein carbonylation etc., as DCF elevation in and of itself is not a marker of oxidative stress in cancer cells. It would also be useful to assess how key antioxidant factors (glutathione, thioredoxin, superoxide dismutase, catalase, GPX4) are altered. Currently the only evidence for the titular premise is the DCFDA staining in Eze-treated cells which (as discussed above) does not adequately a redox-protective for NCP1L1.

4. With NRF2 being higher in MPCs vs. MCs, what are their relative ROS levels? This would be useful to show in support of a redox-associated mechanism underlying DTPs. Peroxides and superoxides drive a number of mitogenic and survival signaling programs and may actually be higher in the MPCs, whereas NCP1L1 may be elevated to protect against 'damaging' species such as lipid peroxides. Such nuanced evaluation would add significant mechanistic depth to this study.

5. Macropinocytosis has a complicated role in tumor cell survival - it has clear tumor-protective effects in a number of tumors through its nutrient scavenging role. Its extreme version (methuosis) is associated with cell death. It is unclear why the authors assign it a tumor-inhibitory role here. Lethal micropinocytosis can also be induced with chalcones. Thus if methuosis is indeed the way to kill MPCs, then chalcones (MIPP, for example) should be as effective as EZE and would support this mechanism of eradication. Or if this is not anticipated, it should be explained why not. Further, if macropinocytosis is critically involved in the process, it may be that MDR relies on macropinocytosis for enhanced cell survival but NPC1L1 inhibition alters lipid metabolism and integrity sufficiently to push the cells into methuosis instead. In this vein, are the parental cells more or less resistant to Baf-1 in conjunction with chemotherapy plus Ver compared to their MDR counterparts? It may be helpful to the authors' premise if they explore this idea. At the very least, they should discuss the tumor-protective role of macropinocytosis and how that may be contravened by the mechanisms they are evaluating.

6. Fig 6B: although there is statistical significance, the magnitude of difference in tumor kinetics between the Eze- and Eze + treatment groups with verapamil nanoparticle conjugates is quite modest. The Ki67 sections provided don't quite support the difference in quantitation provided - the magnitude of staining is very similar. The purported difference might have been clearer had the kinetics been followed beyond the two-week mark. It is recommended that the authors further examine the tumor specimen from these two groups for tumor suppressive markers to support the notion that Eze does a better job at eradicating these tumors than Ver alone - caspase staining for instance.

Textual corrections:
1) As this is a critical premise of the study, please provide the primary article citations rather than just reviews: 'Based on the current knowledge of MDR cancer cells, these special populations are acknowledged to have specific dependency on the antioxidant system.'
2) "KEAP1" is often mis-spelled as "KEEP1"
3) Pg 10: EMT should be 'epithelial- mesenchymal transition', not 'epidermal'
4) Fig S6 - this figure shows only viability (no molecular cell death, Vit E uptake or ROS levels). Please modify the title to reflect what is shown - effects on viability via NPC1L1 modulation. Similarly Fig S7 does not show markers of macropinocytosis, rather effects on viability through use of bafilomycin (and other agents).
5) Fig 4I: Should be DMSO (currently typo DMOS)
6) Please add molecular weight markers to all western blots

Point-by-point response

The revised sections of the manuscript are marked in blue.

Referee #1 (Comments on Novelty/Model System for Author):

Drug Tolerant Persister cells (DTPs) are of major interest in relation to drug resistance. The current manuscript identifies a frequently used drug for treatment of high cholesterol as an effective drug for killing such DTPs that arise after chemotherapy. This is very exciting and novel and has direct utility in the clinic.

Referee #1 (Remarks for Author):

The authors have done a remarkable job in identifying the vulnerabilities of DPTs that arise after treatment of chemotherapy resistant cells that express MDR1 with chemo+MDR1 inhibitors. The data shows that such MDR-DTPs have upregulated NPC1L1, which is activated by an NRF2-mediated the anti-oxidant response in these cells. Ezetimibe is an established inhibitor of NPC1L1 and use of this drug in combination with chemo and verapamil leads to macropinocytosis-mediated cell death. The experiments are well-controlled and in general compelling. The clinical relevance of their finding is immediate, given that ezetimibe is prescribed widely for hypercholesterol therapy without major side effects. Strengths of the manuscript are that the experiments are carried out in two independent MDR resistant cell line models, the deep analyses of the mechanism of DTP survival and the compelling in vivo validation of the drug combinations. I am unable to judge the specifics of the delivery of verapamil in vivo, I trust other reviewers may have an opinion on the suitability of this vehicle. I am highly supportive of immediate publication of this work, given its potential direct clinical application.

Re: Thank you so much for your comments. In this study, we used zeolitic imidazolate MOF-8 (ZIF-8) as a drug delivery platform for verapamil (VER@ZIF-8), which shows the advantages of improved biocompatibility with excellent loading capacity for biomolecules, high physiological constancy without premature drug

release, and good pH responsiveness (Dutta, ***Biomater Sci***, 2021; Su et al., ***Trends Biotechnol***, 2019). To further enhance the on-target activity and selectivity, VER@ZIF-8 was decorated with hyaluronic acid shells to specifically target cancer cells with CD44 receptors, a validated protein for improved delivery of cancer therapeutics (Ghosh et al., ***Expert Opin Ther Targets***, 2012) (**Appendix Figure S3A**). In a similar study to our own, improvement has been achieved in melanomas and breast cancer cells by Zhang and colleagues using methoxy poly (ethylene glycol)-folate (PEG-FA)- (Zhang et al., ***ACS Appl Mater Interfaces***, 2017). In summary, ZIF-8-based nanomaterial has been commonly used as an effective drug delivery system for cancer therapy due to its high surface area and pore volume, in combination with facile modification and chemical functionalization.

Referee #2 (Comments on Novelty/Model System for Author):

The selection and generation of DTP cell lines caused confusion. It seems that in the combination index experiments a drug concentration was chosen that inhibited growth around 30% (?), if the legend in S1G and S1H, blue to white and red to white, respectively, indicate growth inhibition. If that is the case, then a large surviving fraction has been chosen for further study that could contain resistant cells.

Referee #2 (Remarks for Author):

In the study "Inhibition of NPC1L1 disrupts adaptive responses of drug-tolerant persister cells to chemotherapy-induced oxidative stress", Zhang et all describe the isolation and characterization of drug tolerant persistent cells generated from Adriamycin resistant MCF-7 and taxol-resistant Du145 cells. After MDR1 overexpression was established to convey drug-resistance, cells sensitivity was re-established after co-treating cells with the MDR-inhibitor verapamil. Treatment persistent cells were isolated and characterized morphologically and functionally as DTP cells. Gene expression (RNA-seq) analysis compared then MDR cancer cells with residual, regrown MDR cancer cells, and identified NPC1L1 as differential expressed gene and it's upregulation supported cell survival in response to cytotoxic

in MDR-persister cells. The authors further showed that NPC1L1 compromises oxidative stress by promoting vitamin E uptake during chemotherapeutic agents/verapamil treatment in MDR cancer cells. Inhibition of NPC1L1 using ezetimibe suggested cell death by macropinocytosis. NRF2 was shown to be one of the transcription factors targeting the transcription of NPC1L1. Lastly, in a xenograft mouse models, the authors show that taxol/verapamil/ezetimibe delivered via a nanoparticle approach can decrease tumor burden in the xenograft model. Currently third generation p-gp inhibitors are in development. If NPC1L1 turns out to be a desired drug target in cancer to target DTP, then this nanotechnology may be of high importance. However, as PK and PD studies are missing (and beyond the scope), this is difficult to assess. This study offers plentiful of experiments and detail in some instances.

However, overall, the manuscript is very difficult to follow in the beginning where DTPs are isolated or generated. Typos and grammatical errors contribute to this. There is also sometimes a lack of proper rationale and data interpretation.

1. Importantly, the selection and generation of DTP cell lines caused great confusion. As these cells build the foundation of the study, this is an important point. Cells used here were isolated from drug-resistant cancer cell lines. Is the assumption that drug resistant cell lines overexpressing MDR have subpopulations of DTPs? If so, that needs to be stated and convincingly demonstrated. For example, to identify DTP cells, combination index experiments were used. There, a drug concentration that distinguished DTPs from the 'other cells' was chosen that inhibited growth around 30% (?), if the legend in S1G and S1H, blue to white and red to white, respectively, indicate growth inhibition. If that is the case, then this large surviving fraction could easily contain resistant cells. The rationale for this cut off is missing. Would it not be better to find a dose that leads to unaffected growth of resistant cells and isolate cells that grow only modestly?

Re: Thank you very much for your kind suggestion. In recent years, observations from high-resolution analysis of single cells have demonstrated that all the clones

present throughout cancer development may exist before the addition of drugs, but in different clonal frequencies (Marine et al., ***Nat Rev Cancer***, 2020). Indeed, this pre-existence of various clones can be explained by the fact that single tumor cells display a radical transcriptional fluctuation of resistance markers which occurs in a manner similar to that of the so-called transcriptional noise, thus giving rise to an incremental source of transcriptional variability that can predict which tumor cells will ultimately survive drug treatment (Arias and Hayward, ***Nat Rev Genet***, 2006; Shaffer et al., ***Nature***, 2017). As a result, the rare cells whose expression of these resistance-related genes transiently exceeds a certain threshold at treatment initiation, can resist treatment (Qin et al., ***Signal Transduct Target Ther***, 2020). Accompanied by the cumulative exposure duration to drugs, such a stochastic and fluctuated survival mode of this rare subpopulation of tumor cells can differentiate into a stable and heritable state via epigenetic modifications, resulting in a pool of drug tolerant persister cells (DTPs) (Boumahdi and de Sauvage, ***Nat Rev Drug Discov***, 2020; Shaffer et al., ***Nature***, 2017). In contrast to bulk tumor sequencing, this single-cell-based scenario outlines the genesis of DTPs upon treatment and might be applicable to different types of cancer cells, regardless of what status they are in, *e.g.*, MDR-overexpressing cell lines. Based on this notion, we used the two well-established drug-resistant cancer cell lines (Du145^{TXR} and MCF-7^{ADR}) to investigate the molecular mechanism driving the DTP phenotype in MDR-overexpressing cancer cells.

Defining the cancer persistent cell state, *i.e.*, cell cycle arrest; upregulation of drug tolerant rate in comparison with the treatment-naive parental cells; reversibility of the processes in terms of proliferation rate and sensitivity to the same treatment; no occurrence of resistance-conferring mutations (Shen et al., ***Cell***, 2020), would be helpful to avoid confusion with other resistance-related states of tumor cells. In support of this definition, DTPs of Du145^{TXR} and MCF-7^{ADR} cell lines used in our study were shown to have lower proliferative rate (**Appendix Figure S3A-D and Figure EV2D-E**), decreased sensitivity to antitumor agents (**Figure 1C-D**), reversible capacities of proliferative rate and sensitivity following drug withdrawal (**Figure**

1E-G). Moreover, as evidenced by whole-exome sequencing, no additional mutation was found in the DTP cells and the clonal composition remains largely stable. (**Unpublished Figure 1A-B**).

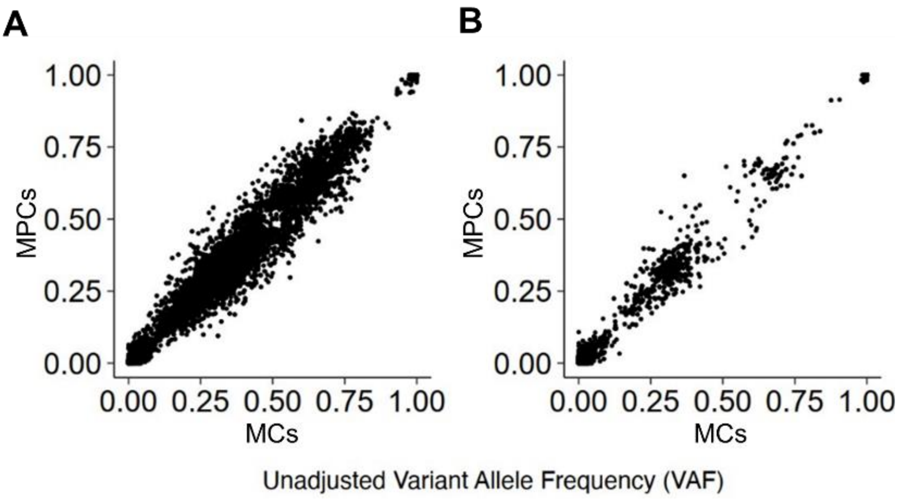

Unpublished Figure 1. **A-B,** scatter plot showing genetic events identified by whole-exome sequencing of MDR cancer cells (MCs) VS. MDR persister cells (MPCs) in (A) Du145^{TXR} or (B) MCF-7^{ADR}.

Given the essential role of MDR1 in maintaining the resistant phenotype of Du145^{TXR} and MCF-7^{ADR} cell lines (**Appendix Figure S1A-C**), the concentration of verapamil, an MDR1 inhibitor, was chosen at a maximum but non-lethal dose to minimize the contributions made by MDR1 to drug efflux (**Appendix Figure 1D-F**). As for the chemotherapeutic agents (taxol or adriamycin), we selected the concentrations which displayed significant inhibitory effects (> 70 percent inhibition) on tumor cells as cut-off values for further exploration (**Appendix Figure 1E-F**). It should be noted that these cut-offs (20 nM taxol/200 nM adriamycin) are larger than the 72 hour IC50 values of their control cell lines. To maximumly isolate DTPs and avoid distractions of resistant cells, these combination strategies (20 nM taxol/200 nM adriamycin plus 50 μM verapamil) were used in Du145^{TXR} and MCF-7^{ADR} cell lines for 72 hours (**Figure 1A**). To address and prevent misunderstanding, we added the related descriptions to the figure legend of **Appendix Figure 1E-F**. In fact, the method of persister cell derivation is relatively standard although there are some

differences compared with previous studies (Hangauer et al., ***Nature***, 2017; Oren et al., ***Nature***, 2021; Shen et al., ***Nat Commun***, 2019; Shen et al., ***Cell Rep***, 2020). Herein, we developed a reliable approach (see details in **the answer to questions 2-3**) by referring to previous studies to identify and validate these residual cancer cells as DTPs.

2. In addition, although text states from line 164 to 167: cells that survived cytotoxic exposure were collected; it is not described in detail how that was done.

Re: Thank you for your valuable comments. In agreement with previous studies (Hangauer et al., ***Nature***, 2017; Oren et al., ***Nature***, 2021; Shen et al., ***Nat Commun***, 2019; Shen et al., ***Cell Rep***, 2020), our method of persister cell collection is relatively standard although we have made some minor alterations. In general, 70 % ~ 90 % of cells are killed and detached by the prescribed treatment. Survival attached cells, that are subsequently defined as persister cancer cells, are trypsinized and washed once in phosphate-buffered saline (PBS) for follow-up exploration. Here, for the control cell lines, persister cells were derived from the treatment of Du145 or MCF-7 with 20 nM taxol or 200 nM adriamycin, respectively, for at least 9 days with fresh drug-containing media; for drug-resistant cancer cells, persister cells were derived upon co-treatment of $Du145^{TXR}$ or $MCF\text{-}7^{ADR}$ with 50 μM verapamil and 20 nM taxol or 200 nM adriamycin, respectively, for 3 days. To help readers better understand, the description of drug-tolerant persister cells derivation has been added into ***Materials and Methods***.

3. What was the rationale for treatment times chosen in 1C, 1D? 1F and 1G are not well described: "drug tolerant rate of (F) Du145/Du145TXR (20 nM taxol / 20 nM taxol + 50 μM verapamil) or (G) MCF-7/MCF-7ADR (200 nM adriamycin / 200 nM adriamycin + 50 μM verapamil) cells treated with drug on the indicated days. Drug tolerant rate defined by compared cell viability followed treatment, RMCs vs. MCs or PeCs vs.PaCs, > 1 representing drug tolerant". Which ratio is referred to? MCF-7/MCF-7ADR or RMCs vs. MCs? If RMCs grow faster that MC, couldn't it be

that a resistant clone is responsible for this phenotype?

Re: Again, thank you for your valuable comments. As described in **the answer to question 2**, a suitably formulated experimental protocol was established by referring to numerous previous studies (Hangauer et al., ***Nature***, 2017; Oren et al., ***Nature***, 2021; Shen et al., ***Nat Commun***, 2019; Shen et al., ***Cell Rep***, 2020). We have added a detailed description regarding the generation of drug-tolerant persister cells into ***Materials and Methods***.

Furthermore, residual MDR cancer cells (RMCs), which were subsequently defined as MDR persister cells (MPCs), displayed a slower proliferative rate than MDR cancer cells (MCs), as evidenced by our data and previous observations that demonstrated a significant cell cycle arrest at G0/G1 phase by flow cytometry and bioinformatic analysis (**Appendix Figure S3B-D and Figure EV2D-E**). Accordingly, we can indirectly reflect the drug tolerant rate by comparing cell viability of persister cells with non-persister cells following the same treatment without interference from growth rate. In order to avoid confusion, we have refined the related description in the revised manuscript.

4. 1D is mislabeled in the legend: MCF-7ADR

Re: Thank you very much. We have corrected this in the revised manuscript.

5. Knocking down MDR should increase sensitivity, this is not novel.

Re: Thank you for the comments. Indeed, ample evidence suggests that the expression of multidrug resistance protein 1 (MDR1, also known as P-glycoprotein or P-gp) confers resistance to cytotoxic therapy. However, as an emerging concept, the DTP state of tumor cells seems to redefine the regulatory mechanisms underlying drug resistance, therefore attracting much attention (Hangauer et al., ***Nature***, 2017; Marin-Bejar et al., ***Cancer Cell***, 2021; Russo et al., ***Science***, 2019). Notably, a recent study published in ***Cell*** (Rehman et al., ***Cell***, 2021) indicated that all the tumor cells, including the MDR1-overexpressing ones, have equal capacity to enter and exit the DTP state to survive drug exposure. In other words, the current strategy of targeting

drug-efflux pumps using verapamil has to deal with the development of further resistance via the DTP stage, which is the reason why we focus on MDR1-derived drug-resistant cancer cells. Indeed, our study reveals that the DTP state does arise in MDR1-overexpressing cells (Du145^{TXR} or MCF-7^{ADR}) during the co-treatment of chemotherapeutic agents and verapamil, thus resulting in suboptimal therapeutic responses both *in vivo* and *in vitro*. Here, in an attempt to improve this situation, we have provided a therapeutic strategy with significant clinical feasibility, that is, ezetimibe (a new cholesterol absorption inhibitor via targeting NPC1L1) in combination with chemotherapy and verapamil. As far as we know, there is no published research investigating the DTP state using MDR cell models, and we have identified NPC1L1 as a novel and promising target for the treatment of DTPs. The main novelty of present study can be summarized in these two aspects. In this regard, our research is of clinical importance to combat DTP-related resistance.

6. Line 171: “MC that have never been treated with” - which drug is referred to? If it is adriamycin or taxol, is that statement then true? How were the resistant cell lines initially made?

Re: Thank you very much for your comments. We apologize for the ambiguity made by inaccurate statements. Here, we intend to indicate the MDR cancer cells (MCs) without the treatment of combinatorial therapy, *i.e.*, chemotherapy combined with verapamil. We have corrected this description in the revised manuscript.

7. Line 182: Fig. S3A: shouldn’t that read Fig. S2A? Pls check entire paragraph for figure mislabelling.

Re: Following your valuable suggestions, we have checked all of the figure labels throughout and corrected the mistakes in the revised manuscript.

8. Line 185-191: this statement is confusing as Figs 1F and 1G don’t show that.

Re: Thanks for your valuable suggestions. In agreement with your comments, we have re-organized the structural framework of Figure 1F-G and rewritten the figure

legends. Furthermore, the related statements have also been polished.

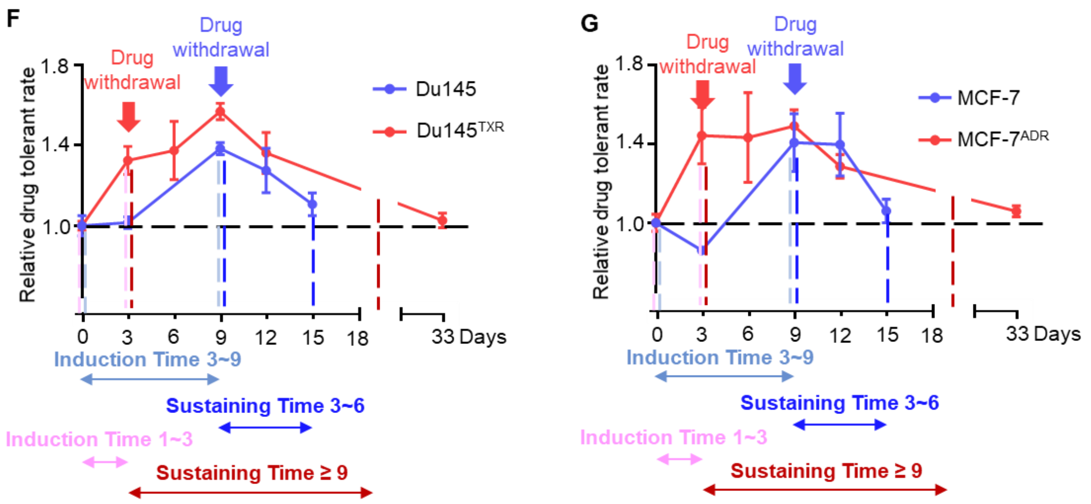

Figure 1. F-G, drug tolerant rate of (F) Du145/Du145^{TXR} (20 nM taxol/20 nM taxol plus 50 μM verapamil) or (G) MCF-7/MCF-7^{ADR} (200 nM adriamycin/200 nM adriamycin plus 50 μM verapamil) cells treated with drugs on the indicated days. Drug tolerant rate defined by comparing cell viability followed treatment, RMCs VS. MCs or CCs VS. PeCs, > 1 representing drug tolerant. Induction Time indicates the time of entering DTP state; Sustaining Time indicates the time of maintaining DTP state. Blue colors represent control group; Red colors represent MDR cancer cells.

9. Regarding the exclusion of autophagy: line 300: not likely caused by autophagy; That needs to be visited in more detail, especially since the autophagy inhibitor bafilomycin a1 was used and a phenotype was found in response to it. Other autophagy markers need to be examined.

Re: Thank you for your valuable comments. Following your suggestions, we examined the alterations in the expression of autophagy-related proteins (Atg5 and LC3B) in the presence of chloroquine (CQ), a late-stage autophagy inhibitor. As shown in **Appendix Figure S4A**, *Atg5*-deficient Du145^{TXR} cells displayed a failed conversion of LC3B-I to lapidated LC3B-II (an established autophagosome marker) due to the lack of Atg5 expression, which is consistent with several previous studies (Lee et al., ***Mol Carcinog***, 2017; Ouyang et al., ***Autophagy***, 2013; Zha et al., ***PLoS One***, 2015). Therefore, we further investigated whether triple-combinatorial treatment

could induce autophagic induction in MCF-7^{ADR} cells. As shown in **Appendix Figure S4B**, triple-combinatorial therapy resulted in marked autophagy induction, as evidenced by increased LC3B turnover and levels of Atg5 and Atg7. In contrast, an early autophagy inhibitor (3-Methyladenine, 3-MA, 1 mM) prominently decreased LC3B lipidation, Atg5, and Atg7 expression in triple-combinatorial therapy-treated MCF-7^{ADR} cells. In addition, to investigate whether autophagy was involved in the anti-cancer effect of triple-combinatorial therapy, Du145^{TXR} and MCF-7^{ADR} cells were treated with triple-combinatorial therapy combined with 3-MA. As shown in **Appendix Figure S4C**, 3-MA showed no obvious influence on cell viability. Consistently, no apparent changes in clonogenic capacity were observed following 3-MA treatment in triple-combinatorial therapy-treated cancer cells (**Appendix Figure S4D-E**). Taken together, our results suggest that triple-combinatorial treatment-induced cell death may not be dependent on autophagy induction.

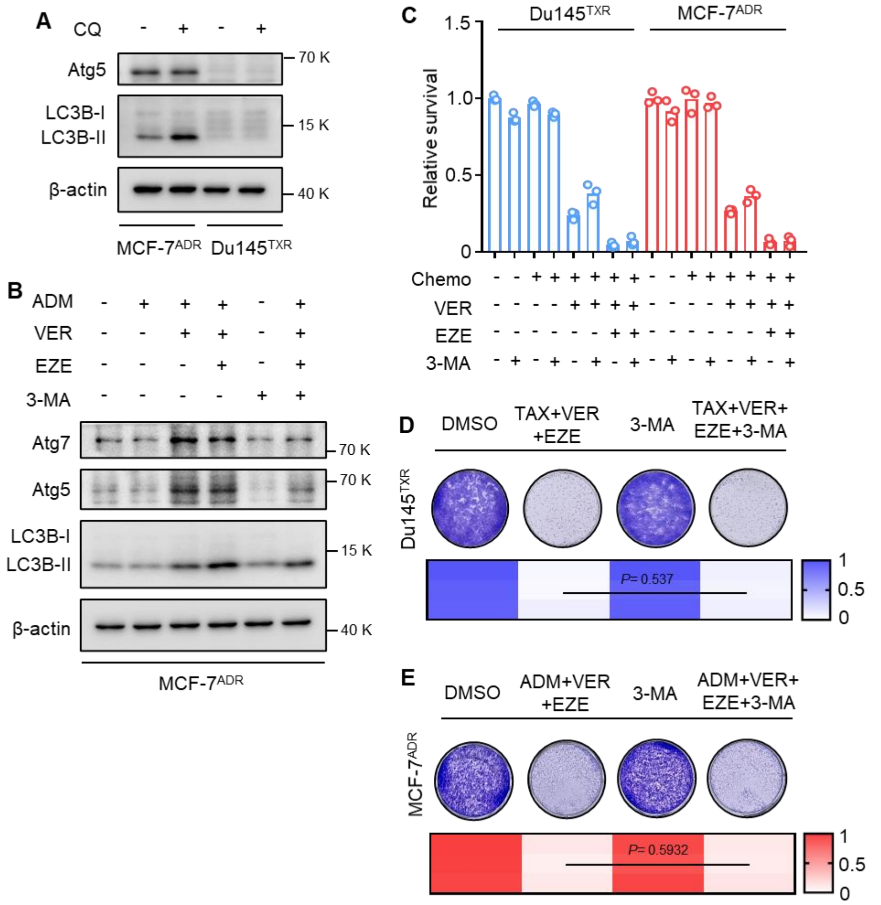

Appendix Figure S4. A, immunoblotting of LC3B, Atg5, in Du145^{TXR} and MCF-7^{ADR} cells treated with or without 10 μM CQ for 24 hours. **B,** immunoblotting

of LC3B, Atg5, and Atg7 in MCF-7^{ADR} cells treated with indicated agents for 24 hours. **C,** cell viability of Du145^{TXR} and MCF-7^{ADR} cells treated with the indicated agents for 72 hours. One-way ANOVA was used to analyze statistical differences. Mean with SD. **D-E,** colony formation assay and quantification of (D) Du145^{TXR} or (E) MCF-7^{ADR} cells treated with the indicated agents. One-way ANOVA was used to analyze statistical differences.

10. Lastly, stemness and EMT are also markers of drug resistant cells, supporting the point made earlier regarding the specificity of the cells identified as DTPs.
Re: Thank you very much for your useful comments. The above general description (**the answer to question 1**) of DTPs in cases of drug exposure or withdrawal represents a commonly applicable paradigm of phenotypic switching in cancer cells, regardless of what types of cancers are treated or what kinds of therapies are used. However, behind this behavior of DTPs, there exist differences in exactly how cancer cells evade therapy, including epithelial-mesenchymal transition (EMT) and acquiring properties of stemness (Boumahdi and de Sauvage, ***Nat Rev Drug Discov***, 2020; Nieto, ***Science***, 2013; Shibue and Weinberg, ***Nat Rev Clin Oncol***, 2017). These functionally comparable processes are associated with the aberrant (re-)activation of developmental programs, suggesting that DTPs may be generated by similar molecular mechanisms (Qin et al., ***Signal Transduct Target Ther***, 2020; Shibue and Weinberg, ***Nat Rev Clin Oncol***, 2017). As for resistance cells, alterations in markers of stemness and EMT can be regarded as a continuation of DTPs.

Referee #3 (Comments on Novelty/Model System for Author):

Experimental design has significant flaws as outlined in my review below.

Referee #3 (Remarks for Author):

In this study, Zhang et al. investigate mechanisms underlying the multidrug resistant (MDR) phenotype in adriamycin-resistant MCF7 cells and taxol-resistant DU145 cells. They propose a drug tolerant persister (DTP) state underlies resistance to

chemotherapeutic drugs and the MDR1 inhibitor verapamil. They further propose this resistance is characterized by resistance to oxidative stress through NRF2-mediated elevation in the cholesterol and alpha-tocopherol transporter, NPC1L1. The authors then use the NPC1L1 inhibitor ezetimibe to show that co-treatment with this agent plus chemotherapy plus verapamil produces ROS production and macropinocytosis-induced cell death in vitro and prevents tumor recurrence in vivo. These studies are interesting and innovative. The occurrence of MDR and DTP in the cell lines used are well-characterized and the siNPC1L1 survival data are convincing. The major weakness is that their main premise of redox homeostasis dysregulation in the MDR process is not convincingly established, and there are some significant conceptual inaccuracies underlying their experimental design and analyses. Furthermore, while the authors have shown a strong link of NPC1L1 to the DTP state, the mechanism by which NPC1L1 inhibition eradicates these cells is also not convincing - it seems ferroptosis would be a more likely mechanism than macropinocytosis given the role of NPC1L1. Overall, some depth and clarification should be provided in these areas to strengthen and streamline the study.
Specific comments are below.

1. Vitamin E has been shown to prevent lipid peroxidation by scavenging lipid peroxyl radicals. It has not been shown to scavenge any other type of radical. DCFDA measures peroxides which do not in fact directly produce lipid peroxides. These are produced by a reaction of the hydroxyl radical (not scavenged by Vit E) with fatty acids, a process facilitated by labile iron. Have the authors assessed whether the siNPC1L1 or Eze induced effects can be rescued by desferal and ferrostatin-1? It's entirely possible that the main effect of siNPC1L1 is ferroptosis given its role as a cholesterol and VitE transporter. The authors should also consider measuring malondialdehyde as a specific product of lipid peroxidation, and whether this is increased by siNPC1L1 or Eze and/or whether VitE or cholesterol addition alter its levels. Regardless it is highly unlikely that Vit E is providing redox protective effects to MPCs by reducing the ROS species being measured by the authors.

Re: Thank you for your critical and useful comments. Using BODIPY 581/591 C11 reagent, we detected the generation of lipid ROS in Du145^{TXR} and MCF-7^{ADR} cells following triple-combinatorial treatment with or without 50 μM cholesterol (CHOL) or 50 μM vitamin E (VE) by flow cytometry. We observed that ezetimibe treatment further elevated lipid ROS accumulation of combination therapy-induced. Both VE and CHOL combined with triple-combinatorial therapy alleviated lipid ROS in Du145^{TXR}. In MCF-7^{ADR} cells, VE but not CHOL led to decreased lipid ROS (**Figure EV4D-F**). In line with the results from ezetimibe, knockdown of NPC1L1 by si*NPC1L1* also resulted in a similar outcome (**Figure EV4G-I**). Furthermore, utilization of either ezetimibe or si*NPC1L1* significantly promoted malondialdehyde (MDA) levels (a specific product of lipid peroxidation) in combination therapy-treated cells, while VE alone compromised triple-combinatorial treatment-induced upregulation of MDA levels (**Figure EV4J-M**). Interestingly, ferroptosis inhibitors including deferoxamine mesylate (DFOM, 5 μM) and ferrostatin-1 (Fer-1, 2 μM) could partially restore NPC1L1 inhibition-triggered cell death in combination therapy-treated cells, evidenced by cell viability and colony formation assays (**Unpublished Figure 2A-H**). Together, our results indicate that lipotoxicity from NPC1L1 inhibition-induced oxidative stress may partially contribute to cell death in persister cells.

However, it is still not clear whether the main effect of NPC1L1 inhibition is attributed to the induction of ferroptosis. As shown in **Figure 4A-B**, we observed a striking cytoplasmic vacuolization upon exposure to triple-combinatorial treatment. Furthermore, a markedly increased uptake of fluorescently labeled high molecular weight dextran (FITC-dextran, a specific substrate of macropinocytosis) was confirmed by immunofluorescence (**Figure 4C-D**). These data reveal that NPC1L1 inhibition using ezetimibe induces macropinocytosis in MPCs. In addition, the vulnerability of persister cancer cells to ferroptosis has been previously reported (Hangauer et al., ***Nature***, 2017; You et al., ***Cancer Lett***, 2021). As such, we focused on triple-combinatorial treatment-induced cytoplasmic vacuolization in MDR cancer cells.

Nevertheless, we still believe that inhibition of NPC1L1 may be associated with the induction of ferroptosis (the result from lipid peroxidation), at least in part. Because macropinocytosis is an endocytic mechanism mediated by plasma membrane structural rearrangements (Puccini et al., ***Trends Cancer***, 2021), NPC1L1 inhibition-triggered oxidative stress could generate peroxidation damage of lipid moleculars containing unsaturated long chain fatty acids on the cell membrane when macropinocytosis occurs. In this respect, the molecular mechanisms underlying these two events, *i.e.*, the induction of macropinocytosis and ferroptosis occurring in a parallel or sequential manner, remain largely elusive, which will be a novel point for our future studies on DTPs.

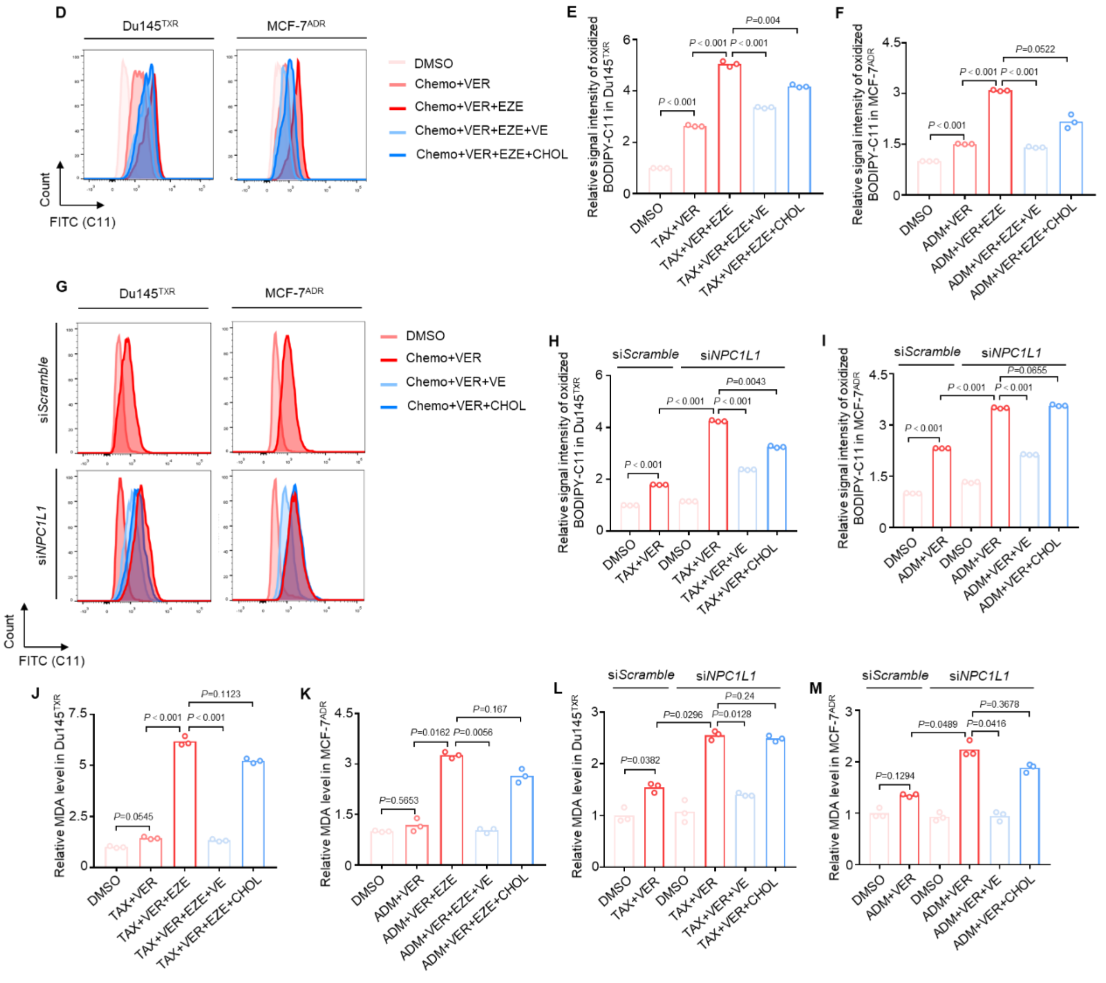

Figure EV4. D-F, flow cytometric analysis of lipid ROS using BODIPY 581/591 C11 reagent in Du145^{TXR} or MCF-7^{ADR} cells treated with indicated agents for 24 hours. Chemo represents 20 nM taxol or 200 nM adriamycin for Du145^{TXR} or MCF-7^{ADR}, respectively. (D) Representative images and quantification of lipid ROS in (E) Du145^{TXR} or (F) MCF-7^{ADR} cells are shown. One-way ANOVA was used to analyze statistical differences. Mean with SD. **G-I,** flow cytometric analysis lipid ROS using BODIPY 581/591 C11 reagent in Du145^{TXR} or MCF-7^{ADR} cells transfected with si*NPC1L1* or si*Scramble* followed by indicated treatment for 24 hours. Chemo represents 20 nM taxol or 200 nM adriamycin for Du145^{TXR} or MCF-7^{ADR},

respectively. (G) Representative images and quantification of lipid ROS in (H) Du145^{TXR} or (I) MCF-7^{ADR} cells are shown. One-way ANOVA was used to analyze statistical differences. Mean with SD. **J-K,** MDA levels measurement of (J) Du145^{TXR} or (K) MCF-7^{ADR} cells treated with indicated agents for 24 hours. One-way ANOVA was used to analyze statistical differences. Mean with SD. **L-M,** MDA levels measurement of (L) Du145^{TXR} or (M) MCF-7^{ADR} cells transfected with si*NPC1L1* or si*Scramble* followed by indicated treatment for 24 hours. One-way ANOVA was used to analyze statistical differences. Mean with SD.

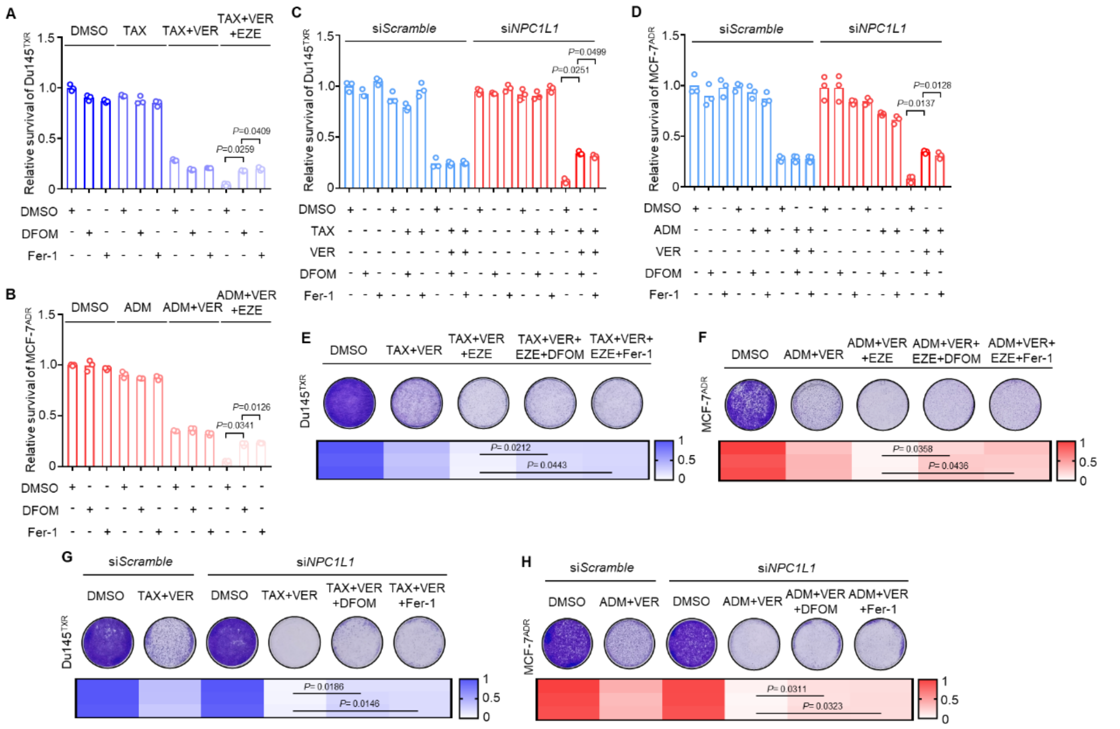

Unpublished Figure 2. **A-B,** cell viability of (A) Du145^{TXR} or (B) MCF-7^{ADR} cells treated with indicated agents for 72 hours. One-way ANOVA was used to analyze statistical differences. Mean with SD. **C-D,** cell viability of (C) Du145^{TXR} or (D) MCF-7^{ADR} cells transfected with si*NPC1L1* or si*Scramble* followed by indicated treatment for 72 hours. One-way ANOVA was used to analyze statistical differences. Mean with SD. **E-F,** colony formation assay and quantification of (E) Du145^{TXR} or (F) MCF-7^{ADR} cells treated with indicated agents. One-way ANOVA was used to analyze statistical differences. **G-H,** colony formation assay and quantification of (E) Du145^{TXR} or (F) MCF-7^{ADR} cells transfected with si*NPC1L1* or si*Scramble* followed

by indicated treatment. One-way ANOVA was used to analyze statistical differences.

2. Secondly, the data provided regarding ROS increase in Fig 3 are not rigorous. ROS-reactive fluorescence quantification via microscopy is notoriously inaccurate/non-homogenous, and quantitative methods such as flow cytometry should be employed.

Re: In agreement with your valuable suggestions, reactive oxygen species (ROS) analysis using the fluorescence probe DCFH-DA was performed again by flow cytometry. The results revealed that the intracellular ROS levels rapidly accumulated following combination treatment within 12 hours, while pharmacological inhibition of NPC1L1 using ezetimibe (25 μM) further enhanced this accumulation which tended to be sustained at a high level for a relatively long time (**Figure 3A-C**).

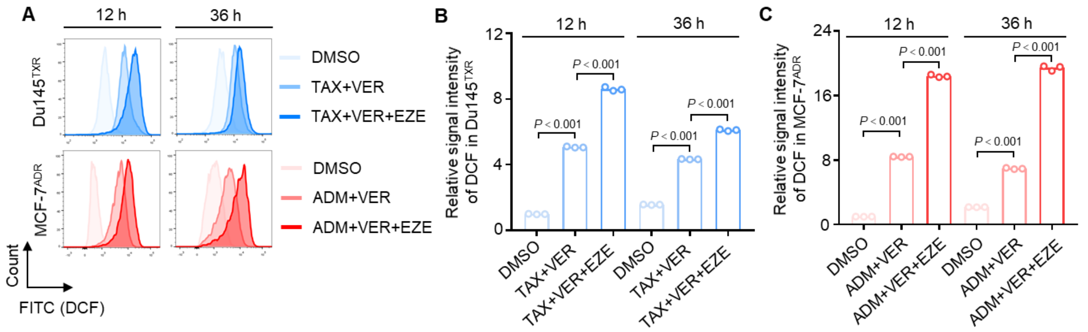

Figure 3. A-C, flow cytometric analysis of ROS levels in Du145^{TXR} or MCF-7^{ADR} cells treated with indicated agents for 12 hours and 36 hours. (A) Representative images and quantification of ROS in (B) Du145^{TXR} or (C) MCF-7^{ADR} cells are shown. One-way ANOVA was used to analyze statistical differences. Mean with SD.

3. Furthermore, the role of ROS needs to be carried through to the other experiments to show convincing involvement of redox dysregulation i.e. do NAC or VE actually reduce ROS levels in addition to promoting survival? If NPCL1L1 is indeed the mediator of increased NRF2 in MPCs then siNPC1L1 should also correlate with redox stress and elevated ROS. These have not been provided. Without such data, the fact that Eze could be having off-target effects on ROS levels, as many drugs do,

cannot be ruled out. Ideally, to invoke oxidative stress via NPC1L1 inhibition, effects should be shown at the level of specific oxidative stress markers such as 8-oxodG, protein carbonylation etc., as DCF elevation in and of itself is not a marker of oxidative stress in cancer cells. It would also be useful to assess how key antioxidant factors (glutathione, thioredoxin, superoxide dismutase, catalase, GPX4) are altered. Currently the only evidence for the titular premise is the DCFDA staining in Eze-treated cells which (as discussed above) does not adequately a redox-protective for NCP1L1.

Re: Thank you very much for your useful comments. Following your valuable suggestions, we examined ROS levels in Du145^{TXR} and MCF-7^{ADR} cells following triple-combinatorial treatment with or without 5 mM N-Acetyl-L-cysteine (NAC) or 50 μM VE by investigating DCF fluorescence intensity with flow cytometry. As shown in **Figure EV3A-C**, triple-combinatorial treatment induced marked accumulation of ROS levels, while this accumulation could be compromised by NAC or VE. Consistently, knockdown of NPC1L1 by si*NPC1L1* significantly enhanced combination treatment-induced ROS accumulation. In contrast, either NAC or VE clearly decreased ROS levels following combination treatment in si*NPC1L1*-transfected cancer cells (**Figure EV3D-F**). Moreover, we observed that ezetimibe induced obvious upregulation of the ratio of oxidized glutathione (GSSG) to reduced glutathione (GSH) in combination treatment-treated cancer cells (**Figure EV3G-H**). Unsurprisingly, *NPC1L1* knockdown induced a similar outcome to ezetimibe (**Figure EV3I-J**). Notably, obvious oxidative damage was also observed in tumors from TAX/(HA/VER@ZIF-8) treatment group as evidenced by increased 8-OHdG (a well-known oxidative stress marker) intensity, while triple-combinatorial treatment displayed further upregulation (**Appendix Figure S5E-F**). Taken together, these data consistently support a role for NPC1L1-mediated antioxidant defense in MPCs.

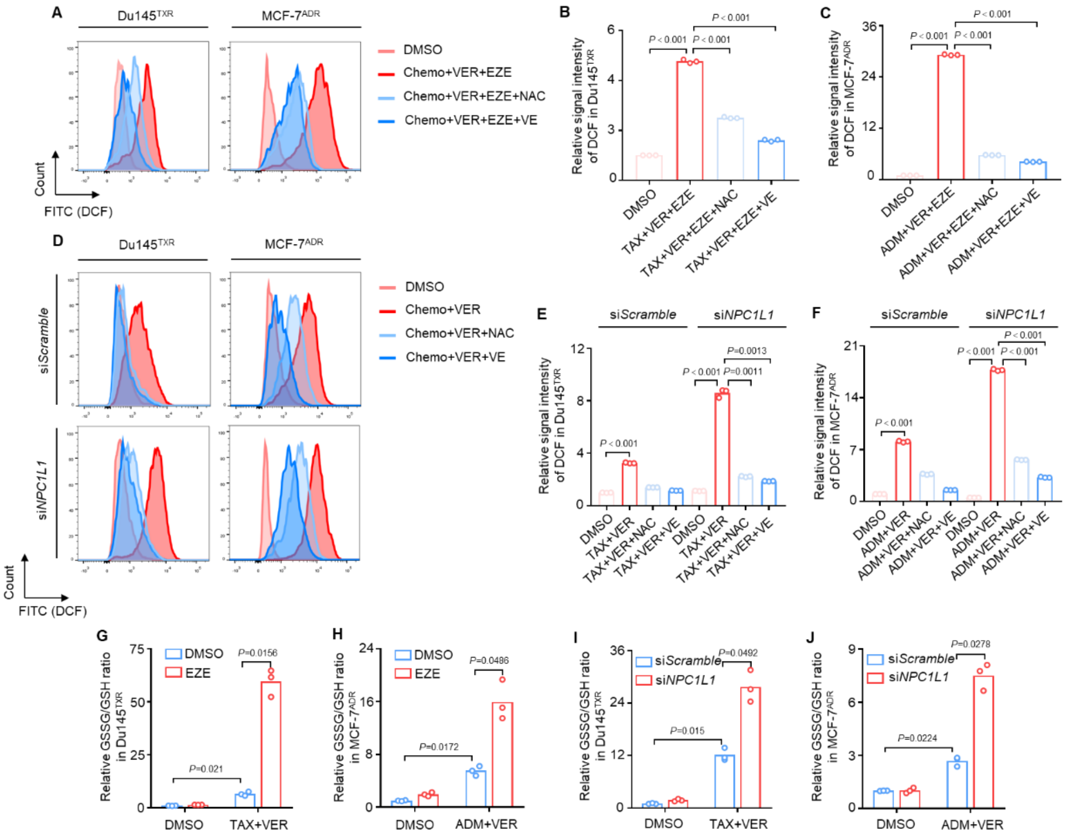

Figure EV3. A-C, flow cytometric analysis of ROS levels in Du145^{TXR} or MCF-7^{ADR} cells treated with indicated agents for 24 hours. Chemo represents 20 nM taxol or 200 nM adriamycin for Du145^{TXR} or MCF-7^{ADR}, respectively. (A) Representative images and quantification of ROS in (B) Du145^{TXR} or (C) MCF-7^{ADR} cells are shown. One-way ANOVA was used to analyze statistical differences. Mean with SD. **D-F,** flow cytometric analysis of ROS levels in Du145^{TXR} or MCF-7^{ADR} cells transfected with si*NPC1L1* or si*Scramble* followed by indicated treatment for 24 hours. Chemo represents 20 nM taxol or 200 nM adriamycin for Du145^{TXR} or MCF-7^{ADR}, respectively. (D) Representative images and quantification of ROS in (E) Du145^{TXR} or (F) MCF-7^{ADR} cells are shown. One-way ANOVA was used to analyze statistical differences. Mean with SD. **G-H,** GSSG/GSH ratio measurement of (G) Du145^{TXR} or (H) MCF-7^{ADR} cells treated with indicated agents for 24 hours. One-way ANOVA was used to analyze statistical differences. Mean with SD. **I-J,** GSSG/GSH ratio measurement of (I) Du145^{TXR} or (J) MCF-7^{ADR} transfected with si*NPC1L1* or si*Scramble* followed by indicated treatment for 24 hours. One-way ANOVA was used to analyze statistical differences. Mean with SD.

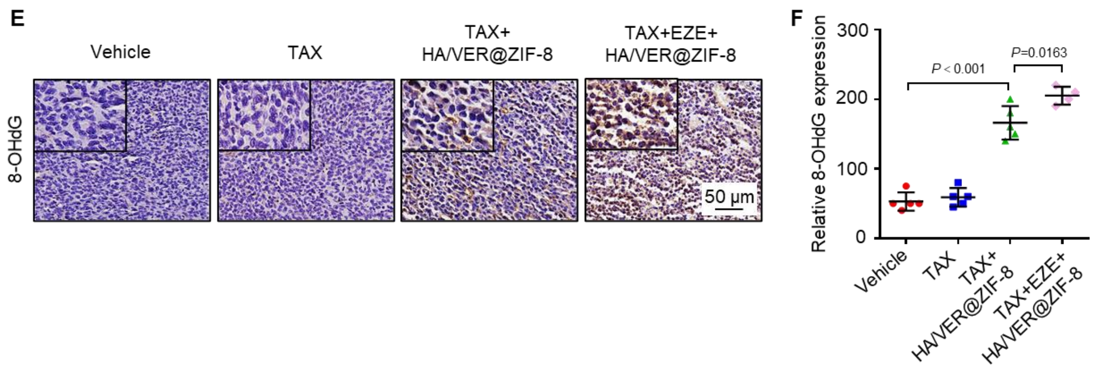

Appendix Figure S5. E-F, immunohistochemical staining of 8-OHdG in indicated groups. (E) Representative images and (F) relative immunohistochemical scores are shown. Scale bar, 50 μm. One-way ANOVA was used to analyze statistical differences. Mean with SD.

4. With NRF2 being higher in MPCs vs. MCs, what are their relative ROS levels? This would be useful to show in support of a redox-associated mechanism underlying DTPs. Peroxides and superoxides drive a number of mitogenic and survival signaling programs and may actually be higher in the MPCs, whereas NCP1L1 may be elevated to protect against 'damaging' species such as lipid peroxides. Such nuanced evaluation would add significant mechanistic depth to this study.

Re: Thanks for your very useful comments. Following your valuable suggestions, we ascertained intracellular ROS levels of MPCs and MCs in Du145^{TXR} or MCF-7^{ADR} by investigating DCF fluorescence intensity with flow cytometry. The results indicated that the intracellular ROS levels of MPCs were slightly higher than those of MCs in both Du145^{TXR} and MCF-7^{ADR} (**Figure EV4R-S**). As you mentioned above, elevated expression of NPC1L1 likely plays a key role in protecting against "damaging" species such as lipid peroxides. In contrast, the observation of higher ROS levels may support ROS-mediated survival signaling in MPCs. We totally concur with this concept and point towards a role for redox state in regulating the survival capacity of DTPs in the revised manuscript.

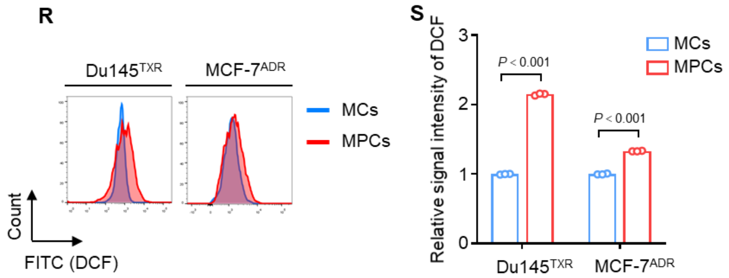

Figure EV4. R-S, flow cytometric analysis of ROS levels in MCs and MPCs of $Du145^{TXR}$ and $MCF\text{-}7^{ADR}$ cells. (R) Representative images and (S) quantification of ROS are shown. Student's t test was used to analyze statistical differences. Mean with SD.

5. Macropinocytosis has a complicated role in tumor cell survival - it has clear tumor-protective effects in a number of tumors through its nutrient scavenging role. Its extreme version (methuosis) is associated with cell death. It is unclear why the authors assign it a tumor-inhibitory role here. Lethal macropinocytosis can also be induced with chalcones. Thus if methuosis is indeed the way to kill MPCs, then chalcones (MIPP, for example) should be as effective as EZE and would support this mechanism of eradication. Or if this is not anticipated, it should be explained why not. Further, if macropinocytosis is critically involved in the process, it may be that MDR relies on macropinocytosis for enhanced cell survival but NPC1L1 inhibition alters lipid metabolism and integrity sufficiently to push the cells into methuosis instead. In this vein, are the parental cells more or less resistant to Baf-1 in conjunction with chemotherapy plus Ver compared to their MDR counterparts? It may be helpful to the authors' premise if they explore this idea. At the very least, they should discuss the tumor-protective role of macropinocytosis and how that may be contravened by the mechanisms they are evaluating.

Re: Thank you for your valuable comments. Indeed, macropinocytosis, as a metabolic adaptation to nutrient stress, usually plays a protective role by promoting nutrient scavenging and anabolism in cancer development. Herein, we demonstrated that macropinocytosis played a lethal role in NPC1L1 inhibitor ezetimibe-induced cell death of MPCs. Nonetheless, the original intention of cancer cells is likely for survival,

through activating macropinocytosis to uptake more antioxidant species (such as VE) against triple-combinatorial treatment-induced oxidative stress. In turn, this activation of macropinocytosis can be hijacked for therapeutic purpose due to the enhanced delivery of anticancer drugs, while inducing a catastrophic and rapid fluid uptake that induces cell death (Hussein et al., ***Drug Resist Updat***, 2021). We have discussed the dual role of micropinocytosis, with a particular emphasis on its therapeutic potential against cancer MDR.

In addition, to ascertain whether activation of macropinocytosis could further promote combination therapy-induced cell death, MDR cancer cells were treated with combination therapy combined with a macropinocytosis inducer vacquinol-1 (VAC, 10 μM) (Colin et al., ***Cancers (Basel)***, 2019). Firstly, the observation using a phase-contrast microscope demonstrated that combination therapy combined with VAC induced numerous single-membrane-bounded vesicles within MDR cancer cells (**Unpublished Figure 3A**). Moreover, as shown in **Unpublished Figure 3B-E**, VAC significantly inhibited the cell growth in combination therapy-treated Du145^{TXR} cells, as evidenced by reduced cell viability and colony formation, while VAC ineffectively sensitized MCF-7^{ADR} cells to combination therapy. The results of MCF-7^{ADR} cells might be attributed to the slight accumulation of empty vesicles following VAC treatment (**Unpublished Figure 3A**). Collectively, these results combined with our previous data demonstrate that excessive activation of macropinocytosis may be an emerging approach to eradicate DTP cells in some tumors. In fact, we are now investigating the role of macropinocytosis against MDR in another study.

Following your kind advice, we also examined whether Baf-A1 influenced the anti-cancer effect of combination therapy (20 nM taxol combined with 50 μM verapamil (VER) treating Du145 or 200 nM adriamycin combined with 50 μM verapamil treating MCF-7 cells). As shown in **Unpublished Figure 3F**, no obvious cytoplasmic vacuolization was observed in combination therapy-treated cancer cells. Furthermore, both cell viability and colony formation assays revealed that Baf-A1 barely counteracted combination therapy-induced growth inhibition (**Unpublished Figure 3G-J**).

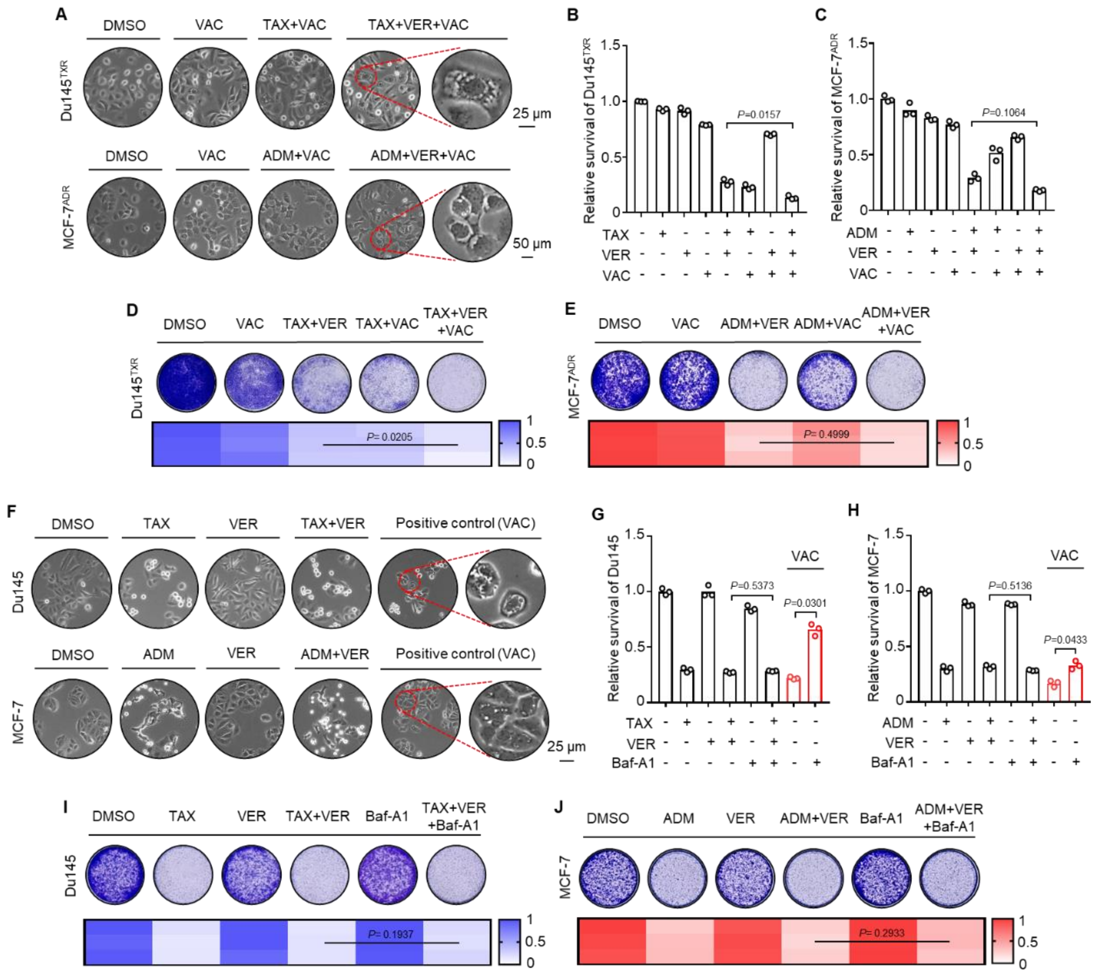

Unpublished Figure 3. **A,** phase contrast images showing the morphological change in Du145^{TXR} and MCF-7^{ADR} cells treated with indicated agents for 24 hours. Scale bars, 25 or 50 μm. **B-C,** cell viability of (B) Du145^{TXR} or (C) MCF-7^{ADR} cells treated with indicated agents for 72 hours. One-way ANOVA was used to analyze statistical differences. Mean with SD. **D-E,** colony formation assay and quantification of (D) Du145^{TXR} or (E) MCF-7^{ADR} cells treated with indicated agents. One-way ANOVA was used to analyze statistical differences. **F,** phase contrast images showing the morphological change in Du145 and MCF-7 cells treated with indicated agents for 24 hours. Scale bars, 25 μm. **G-H,** cell viability of (G) Du145 or (H) MCF-7 cells treated with indicated agents for 72 hours. One-way ANOVA was used to analyze statistical differences. Mean with SD. **I-J,** colony formation assay and quantification of (I) Du145 or (J) MCF-7 cells treated with indicated agents. One-way ANOVA was used to analyze statistical differences.

6. Fig 6B: although there is statistical significance, the magnitude of difference in tumor kinetics between the Eze- and Eze + treatment groups with verapamil nanoparticle conjugates is quite modest. The Ki67 sections provided don't quite support the difference in quantitation provided - the magnitude of staining is very similar. The purported difference might have been clearer had the kinetics been followed beyond the two-week mark. It is recommended that the authors further examine the tumor specimen from these two groups for tumor suppressive markers to support the notion that Eze does a better job at eradicating these tumors than Ver alone - caspase staining for instance.

Re: Following your valuable suggestions, we further examined the cleaved-caspase 3(CC3) expression in xenograft tumors from mice that received different treatments by immunohistochemical (IHC) staining. As shown in **Figure 6F-G**, TAX/(HA/VER@ZIF-8) treatment with Du145^{TXR}-derived xenograft tumors showed stronger CC3 staining levels in comparison with those of the vehicle group or TAX monotherapy, and the triple-combinatorial treatment with TAX/(HA/VER@ZIF-8)/ezetimibe displayed an elevated upregulation of CC3

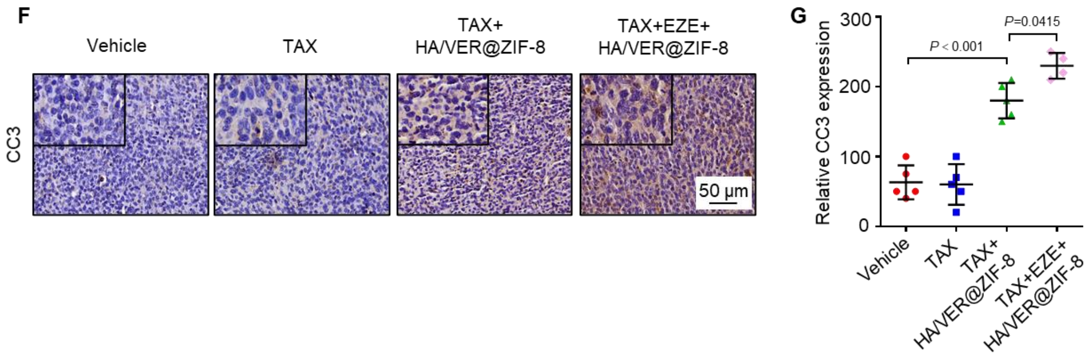

staining.

Figure 6. F-G, immunohistochemical staining of cleaved-caspase 3 (CC3) in indicated groups. (F) Representative images and (G) relative immunohistochemical scores are shown. Scale bar, 50 μm. One-way ANOVA was used to analyze statistical differences. Mean with SD.

Textual corrections:

1) As this is a critical premise of the study, please provide the primary article citations rather than just reviews: 'Based on the current knowledge of MDR cancer cells, these special populations are acknowledged to have specific dependency on the antioxidant system.'

2) "KEAP1" is often mis-spelled as "KEEP1"

3) Pg 10: EMT should be 'epithelial- mesenchymal transition', not 'epidermal'

4) Fig S6 - this figure shows only viability (no molecular cell death, Vit E uptake or ROS levels). Please modify the title to reflect what is shown - effects on viability via NPC1L1 modulation. Similarly Fig S7 does not show markers of macropinocytosis, rather effects on viability through use of bafilomycin (and other agents).

5) Fig 4I: Should be DMSO (currently typo DMOS)

6) Please add molecular weight markers to all western blots

Re: Following your kind suggestions, we have rechecked the manuscript throughout and corrected the mistakes in the revised manuscript.

References

1. Arias, A.M., and Hayward, P. (2006). Filtering transcriptional noise during development: concepts and mechanisms. Nat Rev Genet *7*, 34-44.
2. Boumahdi, S., and de Sauvage, F.J. (2020). The great escape: tumour cell plasticity in resistance to targeted therapy. Nat Rev Drug Discov *19*, 39-56.
3. Colin, M., Delporte, C., Janky, R., Lechon, A.S., Renard, G., Van Antwerpen, P., Maltese, W.A., and Mathieu, V. (2019). Dysregulation of Macropinocytosis Processes in Glioblastomas May Be Exploited to Increase Intracellular Anti-Cancer Drug Levels: The Example of Temozolomide. Cancers (Basel) *11*.
4. Dutta, S. (2021). Immunotherapy of tumors by tailored nano-zeolitic imidazolate framework protected biopharmaceuticals. Biomater Sci *9*, 6391-6402.
5. Ghosh, S.C., Neslihan Alpay, S., and Klostergaard, J. (2012). CD44: a validated target for improved delivery of cancer therapeutics. Expert Opin Ther Targets *16*, 635-650.
6. Hangauer, M.J., Viswanathan, V.S., Ryan, M.J., Bole, D., Eaton, J.K., Matov, A., Galeas, J., Dhruv, H.D., Berens, M.E., Schreiber, S.L., *et al.* (2017). Drug-tolerant persister cancer cells are vulnerable to GPX4 inhibition. Nature *551*, 247-250.
7. Hussein, N.A., Malla, S., Pasternak, M.A., Terrero, D., Brown, N.G., Ashby, C.R., Jr., Assaraf, Y.G., Chen, Z.S., and Tiwari, A.K. (2021). The role of endolysosomal trafficking in anticancer drug resistance. Drug Resist Updat *57*, 100769.
8. Lee, M.S., Tsai, C.W., Wang, C.P., Chen, J.H., and Lin, H.H. (2017). Anti-prostate cancer potential of gossypetin via inducing apoptotic and autophagic cell death. Mol Carcinog *56*, 2578-2592.
9. Marin-Bejar, O., Rogiers, A., Dewaele, M., Femel, J., Karras, P., Pozniak, J., Bervoets, G., Van Raemdonck, N., Pedri, D., Swings, T., *et al.* (2021). Evolutionary predictability of genetic versus nongenetic resistance to anticancer drugs in melanoma. Cancer Cell *39*, 1135-1149 e1138.
10. Marine, J.C., Dawson, S.J., and Dawson, M.A. (2020). Non-genetic mechanisms

of therapeutic resistance in cancer. Nat Rev Cancer *20*, 743-756.

11. Nieto, M.A. (2013). Epithelial plasticity: a common theme in embryonic and cancer cells. Science *342*, 1234850.
12. Oren, Y., Tsabar, M., Cuoco, M.S., Amir-Zilberstein, L., Cabanos, H.F., Hutter, J.C., Hu, B., Thakore, P.I., Tabaka, M., Fulco, C.P., *et al.* (2021). Cycling cancer persister cells arise from lineages with distinct programs. Nature *596*, 576-582.
13. Ouyang, D.Y., Xu, L.H., He, X.H., Zhang, Y.T., Zeng, L.H., Cai, J.Y., and Ren, S. (2013). Autophagy is differentially induced in prostate cancer LNCaP, DU145 and PC-3 cells via distinct splicing profiles of ATG5. Autophagy *9*, 20-32.
14. Puccini, J., Badgley, M.A., and Bar-Sagi, D. (2021). Exploiting cancer's drinking problem: regulation and therapeutic potential of macropinocytosis. Trends Cancer.
15. Qin, S., Jiang, J., Lu, Y., Nice, E.C., Huang, C., Zhang, J., and He, W. (2020). Emerging role of tumor cell plasticity in modifying therapeutic response. Signal Transduct Target Ther *5*, 228.
16. Rehman, S.K., Haynes, J., Collignon, E., Brown, K.R., Wang, Y., Nixon, A.M.L., Bruce, J.P., Wintersinger, J.A., Singh Mer, A., Lo, E.B.L., *et al.* (2021). Colorectal Cancer Cells Enter a Diapause-like DTP State to Survive Chemotherapy. Cell *184*, 226-242 e221.
17. Russo, M., Crisafulli, G., Sogari, A., Reilly, N.M., Arena, S., Lamba, S., Bartolini, A., Amodio, V., Magri, A., Novara, L., *et al.* (2019). Adaptive mutability of colorectal cancers in response to targeted therapies. Science *366*, 1473-1480.
18. Shaffer, S.M., Dunagin, M.C., Torborg, S.R., Torre, E.A., Emert, B., Krepler, C., Beqiri, M., Sproesser, K., Brafford, P.A., Xiao, M., *et al.* (2017). Rare cell variability and drug-induced reprogramming as a mode of cancer drug resistance. Nature *546*, 431-435.
19. Shen, S., Faouzi, S., Bastide, A., Martineau, S., Malka-Mahieu, H., Fu, Y., Sun, X., Mateus, C., Routier, E., Roy, S., *et al.* (2019). An epitranscriptomic mechanism underlies selective mRNA translation remodelling in melanoma persister cells. Nat Commun *10*, 5713.

20. Shen, S., Faouzi, S., Souquere, S., Roy, S., Routier, E., Libenciuc, C., Andre, F., Pierron, G., Scoazec, J.Y., and Robert, C. (2020). Melanoma Persister Cells Are Tolerant to BRAF/MEK Inhibitors via ACOX1-Mediated Fatty Acid Oxidation. Cell Rep *33*, 108421.
21. Shen, S., Vagner, S., and Robert, C. (2020). Persistent Cancer Cells: The Deadly Survivors. Cell *183*, 860-874.
22. Shibue, T., and Weinberg, R.A. (2017). EMT, CSCs, and drug resistance: the mechanistic link and clinical implications. Nat Rev Clin Oncol *14*, 611-629.
23. Su, Y., Cockerill, I., Wang, Y., Qin, Y.X., Chang, L., Zheng, Y., and Zhu, D. (2019). Zinc-Based Biomaterials for Regeneration and Therapy. Trends Biotechnol *37*, 428-441.
24. You, J.H., Lee, J., and Roh, J.L. (2021). Mitochondrial pyruvate carrier 1 regulates ferroptosis in drug-tolerant persister head and neck cancer cells via epithelial-mesenchymal transition. Cancer Lett *507*, 40-54.
25. Zha, Q.B., Zhang, X.Y., Lin, Q.R., Xu, L.H., Zhao, G.X., Pan, H., Zhou, D., Ouyang, D.Y., Liu, Z.H., and He, X.H. (2015). Cucurbitacin E Induces Autophagy via Downregulating mTORC1 Signaling and Upregulating AMPK Activity. PLoS One *10*, e0124355.
26. Zhang, H., Jiang, W., Liu, R., Zhang, J., Zhang, D., Li, Z., and Luan, Y. (2017). Rational Design of Metal Organic Framework Nanocarrier-Based Codelivery System of Doxorubicin Hydrochloride/Verapamil Hydrochloride for Overcoming Multidrug Resistance with Efficient Targeted Cancer Therapy. ACS Appl Mater Interfaces *9*, 19687-19697.

7th Dec 2021

Dear Prof. Wei,

Thank you for the submission of your revised manuscript to EMBO Molecular Medicine. We have now received the enclosed reports from the two referees who re-reviewed your manuscript. As you will see, they are supportive of publication, and I am therefore pleased to inform you that we will be able to accept your manuscript, once the following minor points will be addressed:

1/ Referees' reports: Please address the minor comments from referee #2. As the referee mentions, the manuscript would benefit from text editing, and we would recommend that you have the manuscript edited by a native English speaker. In case this is not an option for you, you might be interested in considering one of the available language editing services e.g. .

2/ Main manuscript text
- Please address the queries from our data editors in track changes mode in the main manuscript file labelled 'Data edited MS file'. Please use this file for any further modification (in track changes mode).
- Please remove the blue text.
- Material and methods:
o Cells: please indicate whether the cells were authenticated (when applicable) and tested for mycoplasma contamination.
o Antibodies: please provide the dilutions used in your study for all experiments.
o Animal studies: please indicate the housing and husbandry conditions.
o Please move the tables to the end of the manuscript, after the main figure legends.
- Acknowledgements: Guangdong Basic and Applied Basic Research Foundation (2019B030302012) and Guangdong Provincial Key Laboratory of Cell Microenvironment and Disease Research (2017B030301018) are missing in the submission system, please update.
- Please rename "Declaration of interest" "Conflicts of interest".
- Please remove the paragraph "Supporting information".

3/ Figures:
- Kindly provide higher quality microscopy images.
- Please upload your movie in a different format, zipped with its legend.
- Please compile the appendix figures and legends in one PDF file with a table of content.

4/ Thank you for providing Source Data. Please upload them as one file per figure.

5/ Thank you for providing The Paper Explained. I added minor modifications, please let me know if you agree with the following, or amend as you see fit:
Problem
The failure of chemotherapy treatment is mostly due to the occurrence of multi-drug resistance (MDR). A subset of residual cells exhibits a transient adaptive resistance mechanism by entering a drug-tolerant persister (DTP) state after treatment. This emerging concept explains the generation of resistant clones with clinically relevant MDR. Accordingly, exploring the specific role and behavior of MDR cancer cells with DTP state in response to current therapy is important for delaying recurrence or even eradicating cancer by targeting specific vulnerabilities.
Results
We identified the acquisition of DTP phenotype in MDR1-mediated MDR cancer cells as a tolerance response to the routine combination of chemotherapeutic agents and MDR1 inhibitor verapamil. MDR cancer cells with DTP state survived chemotherapy-induced oxidative stress primarily by scavenging lipid ROS through NRF2-NPC1L1 axis-regulated vitamin E uptake. Based on nanoparticle-related drug delivery systems to alleviate verapamil side effects in vivo, the combination of chemotherapeutic agents, verapamil, and NPC1L1 inhibitor ezetimibe demonstrated a significant anti-tumor effect and prevented tumor recurrence in mice.
Impact
Our study provides key bearings on the relationship between DTP state and NPC1L1-modulated oxidative stress defense by using MDR1-mediated MDR cancer cells, which establishes a novel therapeutic strategy for treating MDR cancer cells and preventing tumor recurrence.

6/ Thank you for providing a synopsis text. I added minor modifications, please let me know if you agree with the following:
Drug tolerant persister (DTP) state is a driver of therapy failure and cancer relapse. This study identified a key role for NPC1L1 in multi-drug resistant (MDR) cancer cells with the DTP state, where NPC1L1 orchestrated a redox signaling against the harsh environment caused by cancer therapy.
- MDR cancer cells possess a stronger capacity to enter the DTP state than the non-resistant cancer cells.
- NPC1L1 is a key supporter of the DTP state to counter therapy-induced oxidative stress in MDR cancer cells.

- NPC1L1 inhibition interrupts vitamin E uptake, triggering lipid ROS accumulation and consequent lipotoxicity during chemotherapeutic agents/verapamil treatment in MDR cancer cells.
- A combination of chemotherapeutic agents, verapamil, and NPC1L1 inhibitor ezetimibe exerts a significant anti-tumor effect and prevents tumor recurrence in mice.

Please resize your nice synopsis picture to 550 px wide x 300-600 px high. The text should remain legible.

7/ As part of the EMBO Publications transparent editorial process initiative (see our Editorial at http://embomolmed.embopress.org/content/2/9/329), EMBO Molecular Medicine will publish online a Review Process File (RPF) to accompany accepted manuscripts.
This file will be published in conjunction with your paper and will include the anonymous referee reports, your point-by-point response and all pertinent correspondence relating to the manuscript. Let us know whether you agree with the publication of the RPF and as here, IF YOU WANT TO REMOVE OR NOT any figures from it prior to publication.
Please note that the Authors checklist will be published at the end of the RPF.

I look forward to receiving your revised manuscript.

Yours sincerely,

Lise Roth

Lise Roth, PhD
Editor
EMBO Molecular Medicine

***** Reviewer's comments *****

Referee #1 (Comments on Novelty/Model System for Author):

two models used yielding similar results

Referee #1 (Remarks for Author):

I have evaluated the responses of the authors to reviewer 3. I find these responses to be satisfactory. My own comments were also well-addressed but minor in nature.

Referee #2 (Remarks for Author):

Thank you, all points were addressed. However, the manuscript has still information missing here and there, e.g. drug concentrations used for Baf1a, 3-MA etc. Please carefully got through the manuscript and ensure all details are in place. Also, an editorial review regarding grammar needs to be done.

The authors performed the requested editorial changes.

14th Dec 2021

Dear Prof. Wei,

Thank you for submitting your revised files. I have looked at everything, and I am pleased to inform you that your manuscript is now accepted for publication in EMBO Molecular Medicine!

I have added minor text modification (doc attached), please amend as you see fit and send us your manuscript (with accepted changes) via email. It will then be sent to our publisher to be included in the next available issue of EMBO Molecular Medicine.

Please read below for additional IMPORTANT information regarding your article, its publication and the production process.

Congratulations on your interesting work,

With kind regards

Lise Roth

Lise Roth, Ph.D
Scientific Editor
EMBO Molecular Medicine

EMBO PRESS

YOU MUST COMPLETE ALL CELLS WITH A PINK BACKGROUND

PLEASE NOTE THAT THIS CHECKLIST WILL BE PUBLISHED ALONGSIDE YOUR PAPER

Corresponding Author Name: Xiawei Wei
Journal Submitted to: EMBO Molecular Medicine
Manuscript Number: EMM-2021-14903

USEFUL LINKS FOR COMPLETING THIS FORM

http://www.antibodypedia.com
http://1degreebio.org
http://www.equator-network.org/reporting-guidelines/improving-bioscience-research-repor
http://grants.nih.gov/grants/olaw/olaw.htm
http://www.mrc.ac.uk/Ourresearch/Ethicsresearchguidance/Useofanimals/index.htm
http://ClinicalTrials.gov
http://www.consort-statement.org
http://www.consort-statement.org/checklists/view/32-consort/66-title
http://www.equator-network.org/reporting-guidelines/reporting-recommendations-for-tumo
http://datadryad.org
http://figshare.com
http://www.ncbi.nlm.nih.gov/gap
http://www.ebi.ac.uk/ega
http://biomodels.net/
http://biomodels.net/miriam/
http://jjj.biochem.sun.ac.za
https://osp.od.nih.gov/biosafety-biosecurity-and-emerging-biotechnology/
http://www.selectagents.gov/

Reporting Checklist For Life Sciences Articles (Rev. June 2017)

This checklist is used to ensure good reporting standards and to improve the reproducibility of published results. These guidelines are consistent with the Principles and Guidelines for Reporting Preclinical Research issued by the NIH in 2014. Please follow the journal's authorship guidelines in preparing your manuscript.

A- Figures
1. Data
The data shown in figures should satisfy the following conditions:

- the data were obtained and processed according to the field's best practice and are presented to reflect the results of the experiments in an accurate and unbiased manner.
- figure panels include only data points, measurements or observations that can be compared to each other in a scientifically meaningful way.
- graphs include clearly labeled error bars for independent experiments and sample sizes. Unless justified, error bars should not be shown for technical replicates.
- if n< 5, the individual data points from each experiment should be plotted and any statistical test employed should be justified
- Source Data should be included to report the data underlying graphs. Please follow the guidelines set out in the author ship guidelines on Data Presentation.

2. Captions

Each figure caption should contain the following information, for each panel where they are relevant:

- a specification of the experimental system investigated (eg cell line, species name).
- the assay(s) and method(s) used to carry out the reported observations and measurements
- an explicit mention of the biological and chemical entity(ies) that are being measured.
- an explicit mention of the biological and chemical entity(ies) that are altered/varied/perturbed in a controlled manner.
- the exact sample size (n) for each experimental group/condition, given as a number, not a range;
- a description of the sample collection allowing the reader to understand whether the samples represent technical or biological replicates (including how many animals, litters, cultures, etc.).
- a statement of how many times the experiment shown was independently replicated in the laboratory.
- definitions of statistical methods and measures:
  - common tests, such as t-test (please specify whether paired vs. unpaired), simple χ2 tests, Wilcoxon and Mann-Whitney tests, can be unambiguously identified by name only, but more complex techniques should be described in the methods section;
  - are tests one-sided or two-sided?
  - are there adjustments for multiple comparisons?
  - exact statistical test results, e.g., P values = x but not P values < x;
  - definition of 'center values' as median or average;
  - definition of error bars as s.d. or s.e.m.

Any descriptions too long for the figure legend should be included in the methods section and/or with the source data.

In the pink boxes below, please ensure that the answers to the following questions are reported in the manuscript itself. Every question should be answered. If the question is not relevant to your research, please write NA (non applicable). We encourage you to include a specific subsection in the methods section for statistics, reagents, animal models and human subjects.

B- Statistics and general methods

Please fill out these boxes (Do not worry if you cannot see all your text once you press return)

1.a. How was the sample size chosen to ensure adequate power to detect a pre-specified effect size?	Sample size was determined to be adequate based on the magnitude and consistency of measurable differences between groups in all experiments in this study.
1.b. For animal studies, include a statement about sample size estimate even if no statistical methods were used.	We described in Material and Methods sections. At least 5 independent mice per experimental set.
2. Describe inclusion/exclusion criteria if samples or animals were excluded from the analysis. Were the criteria pre-established?	No animals were excluded from the study.
3. Were any steps taken to minimize the effects of subjective bias when allocating animals/samples to treatment (e.g. randomization procedure)? If yes, please describe.	The treatment protocols were compared to Placebo control in tumor transplanted mice. The isolated tumor samples were randomized and blinded after euthanasia of the mice for further histopathological investigations.
For animal studies, include a statement about randomization even if no randomization was used.	Randomization was used.
4.a. Were any steps taken to minimize the effects of subjective bias during group allocation or/and when assessing results (e.g. blinding of the investigator)? If yes please describe.	Yes, when performing histopathological analysis, slides were examined independently by different members of the laboratory.
4.b. For animal studies, include a statement about blinding even if no blinding was done	No blinding was done, yet mice were supervised by animal care takers not involved in the actual experiment.
5. For every figure, are statistical tests justified as appropriate?	Yes, statistical tests are descibed in "Meterials and Methods-Statistics" section and individual figure legends
Do the data meet the assumptions of the tests (e.g., normal distribution)? Describe any methods used to assess it.	Yes
Is there an estimate of variation within each group of data?	Yes, variances are displayed in the individual figures by presenting all data points or relevant error bars.

Is the variance similar between the groups that are being statistically compared?	Yes, classical statistical analysis was run using the GraphPad Prism software 7 (GraphPad 7). Depending on experiments, two-group data were analyzed using Student t-test. Multi-group data were analyzed using one-way ANOVA. All values are mean SD unless otherwise indicated. Differences were considered statistically significant when P< 0.05 (*P<0.05, **P<0.01, ***P<0.001).

C- Reagents

6. To show that antibodies were profiled for use in the system under study (assay and species), provide a citation, catalog number and/or clone number, supplementary information or reference to an antibody validation profile. e.g., Antibodypedia (see link list at top right), 1DegreeBio (see link list at top right).	Company and catalog number shave been provided wherever appropriate. See Materials and Methods section.
7. Identify the source of cell lines and report if they were recently authenticated (e.g., by STR profiling) and tested for mycoplasma contamination.	All cell lines were obtained from a neighboring laboratory. All cell lines were recently authenticated by STR profiling. In addition, only non-contaminated cell lines were used for experiments.

* for all hyperlinks, please see the table at the top right of the document

D- Animal Models

8. Report species, strain, gender, age of animals and genetic modification status where applicable. Please detail housing and husbandry conditions and the source of animals.	All animal experiments were approved by the Institutional Animal Care and Use Committee of Sichuan University. All male BALB/c nude (CAnN.Cg-Foxn1nu/Crl) mice (seven-week old) were obtained from HFK Bioscience Co., Ltd (Beijing, China). All mice were housed in cages contained aspen chip bedding in rooms under standard condition (temperature: 20-22°C, relative humidity: 61-65%, 12 h light/dark cycle, 5 mice per cage) in accordance with institutional guidelines from Animal Care and Use Committee of Sichuan University. Mice had free access to tap water and standard commercial mouse chow (HFK Bioscience Co., Ltd, Beijing, China).
9. For experiments involving live vertebrates, include a statement of compliance with ethical regulations and identify the committee(s) approving the experiments.	All animal experiments were reviewed and approved by the Institutional Animal Care and Treatment Committee of Sichuan University.
10. We recommend consulting the ARRIVE guidelines (see link list at top right) (PLoS Biol. 8(6), e1000412, 2010) to ensure that other relevant aspects of animal studies are adequately reported. See author guidelines, under 'Reporting Guidelines'. See also: NIH (see link list at top right) and MRC (see link list at top right) recommendations. Please confirm compliance.	Confirmed

E- Human Subjects

11. Identify the committee(s) approving the study protocol.	N/A
12. Include a statement confirming that informed consent was obtained from all subjects and that the experiments conformed to the principles set out in the WMA Declaration of Helsinki and the Department of Health and Human Services Belmont Report.	N/A
13. For publication of patient photos, include a statement confirming that consent to publish was obtained.	N/A
14. Report any restrictions on the availability (and/or on the use) of human data or samples.	N/A
15. Report the clinical trial registration number (at ClinicalTrials.gov or equivalent), where applicable.	N/A
16. For phase II and III randomized controlled trials, please refer to the CONSORT flow diagram (see link list at top right) and submit the CONSORT checklist (see link list at top right) with your submission. See author guidelines, under 'Reporting Guidelines'. Please confirm you have submitted this list.	N/A
17. For tumor marker prognostic studies, we recommend that you follow the REMARK reporting guidelines (see link list at top right). See author guidelines, under 'Reporting Guidelines'. Please confirm you have followed these guidelines.	N/A

F- Data Accessibility

18: Provide a "Data Availability" section at the end of the Materials & Methods, listing the accession codes for data generated in this study and deposited in a public database (e.g. RNA-Seq data: Gene Expression Omnibus GSE39462, Proteomics data: PRIDE PXD000208 etc.) Please refer to our author guidelines for 'Data Deposition'.<br><br>Data deposition in a public repository is mandatory for:<br>a. Protein, DNA and RNA sequences<br>b. Macromolecular structures<br>c. Crystallographic data for small molecules<br>d. Functional genomics data<br>e. Proteomics and molecular interactions	This study generated data (RNA sequencing of MCs and MPCs) deposited in the SRA database (https://www.ncbi.nlm.nih.gov/sra) with the accession numbers PRJNA777049 and in the GEO database (https://www.ncbi.nlm.nih.gov/geo) with the accession numbers GSE187441.
19. Deposition is strongly recommended for any datasets that are central and integral to the study; please consider the journal's data policy. If no structured public repository exists for a given data type, we encourage the provision of datasets in the manuscript as a Supplementary Document (see author guidelines under 'Expanded View' or in unstructured repositories such as Dryad (see link list at top right) or Figshare (see link list at top right).	The data has been released in SRA (PRJNA777049) and GEO (GSE187441) dataset.
20. Access to human clinical and genomic datasets should be provided with as few restrictions as possible while respecting ethical obligations to the patients and relevant medical and legal issues. If practically possible and compatible with the individual consent agreement used in the study, such data should be deposited in one of the major public access-controlled repositories such as dbGAP (see link list at top right) or EGA (see link list at top right).	N/A
21. Computational models that are central and integral to a study should be shared without restrictions and provided in a machine-readable form. The relevant accession numbers or links should be provided. When possible, standardized format (SBML, CellML) should be used instead of scripts (e.g. MATLAB). Authors are strongly encouraged to follow the MIRIAM guidelines (see link list at top right) and deposit their model in a public database such as Biomodels (see link list at top right) or JWS Online (see link list at top right). If computer source code is provided with the paper, it should be deposited in a public repository or included in supplementary information.	N/A

G- Dual use research of concern

22. Could your study fall under dual use research restrictions? Please check biosecurity documents (see link list at top right) and list of select agents and toxins (APHIS/CDC) (see link list at top right). According to our biosecurity guidelines, provide a statement only if it could.	No
